# Multi-step biocatalytic strategy to produce a library of original xylosides with various ester functions for cosmetic applications

**DOI:** 10.1039/d5ra09500j

**Published:** 2026-01-26

**Authors:** Emelyne Jolly, Murielle Muzard, Richard Plantier-Royon, Caroline Rémond

**Affiliations:** a Université de Reims Champagne-Ardenne, CNRS, ICMR Reims France richard.plantier-royon@univ-reims.fr; b Université de Reims Champagne-Ardenne, INRAE, FARE, UMR A 614, AFERE Reims France caroline.remond@univ-reims.fr

## Abstract

A biocatalytic strategy was developed to prepare a series of β-xylopyranosides with one or two ester functions using a multistep enzymatic transformation from biobased xylans. Transglycosylation reactions with α,ω-diols were first performed using a commercially available xylanase to produce β-xylosides in good yields. In a second step, a selective esterification of the primary hydroxyl group of the aglycone moiety was carried out by reaction with carboxylic acids of cosmetic interest catalyzed by an immobilized commercial lipase. Finally a transesterification step led to the formation of xylosides esterified with two different acyl chains.

## Abstract 2

A biocatalytic strategy was developed to prepare a series of β-xylopyranosides with one or two ester functions using a multistep enzymatic transformation from biobased xylans.

## Graphical abstract

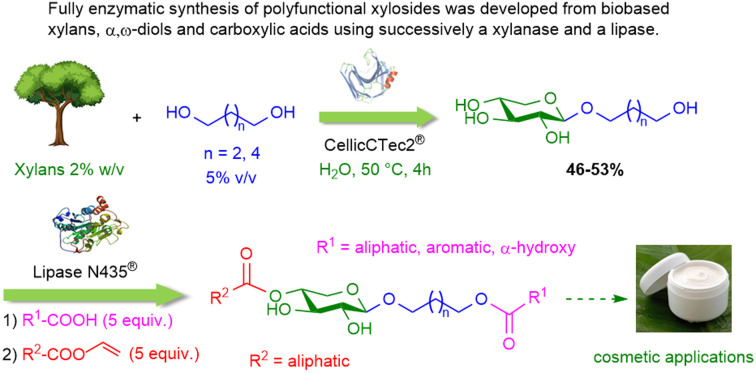

## 1. Introduction

For several decades now, the global cosmetics market has been booming with rapid change and strong worldwide demand. More recently, as a result of the current international regulations and, above all, consumer demand, the cosmetics industry is now faced with the challenge of maintaining a high capacity for innovation and developing active ingredients that are both healthier and more natural to replace petroleum-based compounds.^1–3^ Therefore, over the last 20 years, the cosmetic industry has increasingly used active ingredients derived from biobased compounds to prepare cleaner formulations, giving them an undeniable marketing advantage. Multifunctional molecules can be defined as molecules providing two or more benefits within a cosmetic formulation. These effects may include moisturizing, anti-aging, antimicrobial, anti-inflammatory, UV protection, skin brightening, or texture-enhancing functions. Multifunctional molecules offer many advantages, in particular formulations with a reduced list of active ingredients, which are cheaper and easier to manufacture.^4,5^ Some molecules are naturally multifunctional, as it is the case for resveratrol, and display several properties such as antioxidant, anti-microbial and skin-lightening through tyrosinase inhibition.^6,7^

Some multifunctional molecules can be synthesized by combining different molecules possessing two or more distinct functional groups in their chemical structure. The development of such polyfunctional compounds prepared from several molecules known to be active ingredients of interest in a single chemical structure represents an emerging area of research for cosmetic formulations. In this context, green chemistry can greatly contribute to the emergence of new molecules for cosmetics through the use of sustainable raw materials, the development of eco-respectful processes and the synthesis of molecules with low-environmental impact.^8^

For more than 40 years, it has been shown that β-d-xylopyranosides featuring hydrophobic aglycone moieties (aliphatic, aromatic, polyaromatic) can act as primers for an exogenous synthesis of glycosaminoglycans (GAGs).^9–11^ In humans, these linear sulfated polysaccharide GAG side chains displayed various biological functions such as modulating signal transduction, cell proliferation, angiogenesis, adhesion and migration.^12,13^ For example, naphthyl xylosides have been extensively studied for their applications as antiproliferative agents,^14,15^ or ‘click’ xylosides also showed selective inhibition of cancer cell growth by inducing GAG synthesis.^16^ In cosmetic skincare applications, a *C*-xyloside, developed by L’Oréal in the early 21st century and known as Pro-Xylane™, exhibited a contribution to the renewal of GAGs in skin and permitted the restoration of matrix architecture and skin hydration.^8,17,18^

In addition, many carboxylic acids are widely used in cosmetic formulations. Fatty acids (FAs) can be included in almost any skincare formulation and make an excellent hydration booster in moisturizers and cleansers. Hydroxy acids and especially α-hydroxy acids (AHAs) such as glycolic acid, lactic acid or mandelic acid, are a class of organic acid commonly used in skin care products. AHAs are usually applied to treat acne, scars, hyperpigmentation, age spots, they can also improve wrinkled skin by increasing the synthesis of glycosaminoglycans and thickening skin.^19,20^ However, these compounds have shown limited applicability due to their irritant effects attributed to their acidities. Phenolic acids (PAs) such as hydroxybenzoic acid derivatives or hydroxycinnamic acids derivatives are well-known as strong antioxidant agents and free radical scavengers but also for their photoprotective, antibacterial and anti-inflammatory properties.^21,22^.

**Fig. 1:**
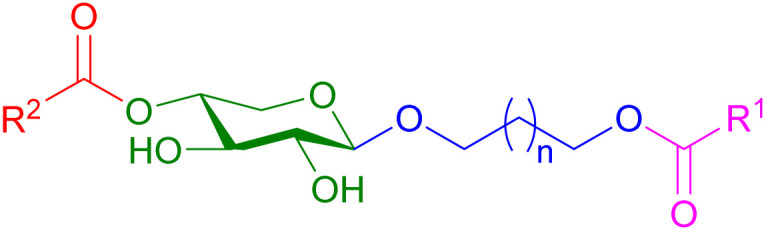
General structure for the target polyfunctional xylosides.

In this work, the synthesis of xylosides containing ester functions of different types was investigated (Fig. 1). To prepare these molecules, a biocatalytic route using commercial enzymes was used for both the glycosylation and esterification reactions to limit the environmental impact of the synthesis. Compared to conventional synthesis routes, biocatalytic approaches occur under moderate temperature and pH conditions. Several studies have shown that the addition of aliphatic chains or phenolic groups to hydrophilic molecules through glycosylation or esterification reactions alters their hydrophilic/lipophilic balance and could improve their penetration into animal cells and their absorption through the skin barrier.^23,24^ This was observed for xylosides harboring a hydrophobic aglycone moiety which could be absorbed by cells and prime GAGs biosynthesis.^25^ Esterases and lipases, widely produced and distributed in human and animal tissues forming the skin as well as commensal bacteria in the human skin, play various biological roles such as the hydrolysis of ester bonds during drug metabolism.^23,26–28^ These enzymes could thus participate to the *in vivo* hydrolysis of the xyloside esters targeted in this study, thereby gradually releasing each constituent of interest, xyloside and carboxylic acids, for cosmetic applications.

In previous studies, the preparation of various xylosides by transglycosylation reactions from xylans or directly from a lignocellulosic biomass co-product such as wheat bran has been described for applications in the fields of surfactants,^29,30^ ionic liquids^31^ or the activation of GAG biosynthesis.^25,32,33^ The enzymatic synthesis of esters derived from xylose and xylo-oligosaccharides using an immobilized commercial lipase has also been reported for applications as surfactants.^30,34,35^ In the present study, this biocatalytic strategy combining cascade reactions through glycosylation and acylation reactions was conducted in presence of biobased molecules as well as xylans from lignocellulosic biomass leading to the synthesis of polyfunctional xylosides.

## 2. Results and discussion

### 2.1. Synthesis of diol-based xylosides

Glycoside hydrolases naturally cleave glycosidic bonds by acid–base catalysis. These enzymes can also catalyze the transglycosylation of glycosyl residues to alcohols, producing alkyl glycosides.^36,37^ Preparation of glycosides using an enzymatic pathway *via* reverse hydrolysis or transglycosylation reactions represents an interesting and promising approach due to the use of unprotected carbohydrates, the high selectivity of the anomeric linkage and the mild reaction conditions. Xylanases can therefore produce alkyl xylosides and alkyl oligoxylosides, in a single step, without the formation of unwanted by-products and the generation of non-recyclable waste, in opposition to the chemical route.

In a previous study, the ability of the enzyme CellicCtec2® to synthesize alkyl xylosides from wheat bran was demonstrated.^30^ The xylanase (EC 3.2.1.8) present within the enzymatic cocktail was able to catalyze transglycosylation reactions in the presence of xylans and primary alcohols such as pentan-1-ol.

**Scheme 1:**
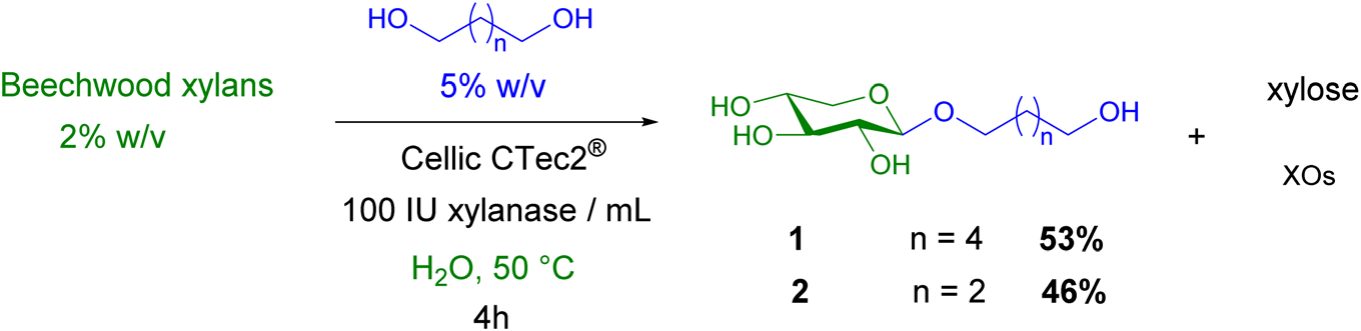
Enzymatic tranglycosylation reactions from xylans and α,ω-diols.

Here, the ability of the CellicCtec2® enzyme to catalyze transglycosylation in the presence of hexane-1,6-diol or butane-1,4-diol as acceptors and commercially available beechwood xylans as donors was evaluated. Firstly, the reactions tested on small volumes (2 mL) indicated that xylosides 1-2 were detected after 2 hours of reaction and remained present along the 24 hours of reaction (TLC analysis; SI). In parallel to the transglycosylation reaction, a competitive hydrolysis reaction occurred leading to the release of xylose and xylo-oligosaccharides from the xylans (Scheme 1).

Higher scale reactions were conducted to prepare some grams of xylosides. Reactions were performed in the presence of xylans (2% w/v) as donors. After 4 hours of reaction, the crude material was centrifugated, water was removed under reduced pressure and the crude solid was extracted with EtOAc/MeOH 90 : 10. Solvents were removed under reduced pressure and the residue was purified by silica gel chromatography to obtain pure xylosides. Transglycosylation yields were 53% and 46% for reactions with hexane-1,6-diol and butane-1,4-diol respectively. The efficiency of the CellicCtec2® to catalyze transglycosylation reactions was quite similar for both tested diols indicating that the acceptor chain length was not a limiting parameter. Previous studies have demonstrated that when primary alcohols are used as acceptors, there is no negative impact on the efficiency of transglycosylation catalyzed by some xylanases when alcohols chain lengths are lower to 8 carbon atoms.^29^

### 2.2. Synthesis of a library of esterified xylosides

Lipases (EC 3.1.1.3) are versatile biocatalysts displaying high potential for the synthesis of esters as demonstrated by the abundant reported literature.^38–40^ The well-known lipase N435® used in the present study corresponds to the lipase B from *Candida antarctica*, heterologously expressed with *Aspergillus oryzae* and immobilized on a solid macroporous acrylic support.^41^

**Scheme 2:**
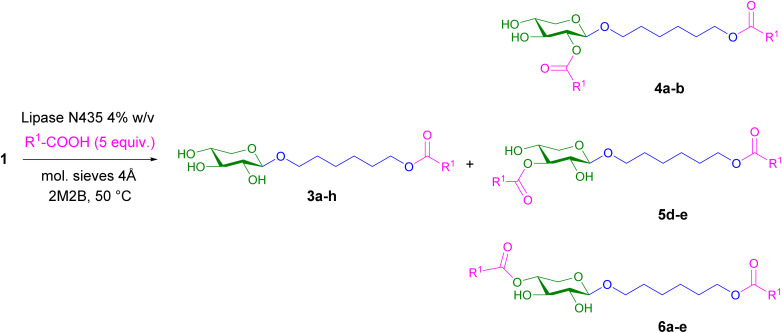
Enzymatic acylation of xyloside 1.

Xyloside 1 was selected as a template to get access to a library of esterified xylosides. The acylation reactions were catalyzed by lipase N435® and various carboxylic acids were tested as acyl donors (Scheme 2 and Table 1). The best reaction conditions were determined using an excess of carboxylic acids, the 1 : 5 ratio being selected. Xyloside 1 (1 eq.) and carboxylic acid (5 eq.) were solubilized in anhydrous 2-methylbutan-2-ol (2M2B). After addition of molecular sieves and immobilized lipase, the reaction mixture was stirred at 50 °C during 72 h. The liquid phase was removed, centrifugated and solvent was eliminated under reduced pressure. The residue was purified by silica gel chromatography. It is important to note that both the glycone and aglycone moieties of the xyloside can act as acyl acceptor during the biocatalytic reaction.

**Table 1:** Enzymatic acylation of xyloside 1^a^

	RCOOH	Yields (%)			
		Monoester 3	Diester *O*-2 4	Diester *O*-3 5	Diester *O*-4 6
a	Octanoic acid	40	11	nd	21
b	Lauric acid	45	9	nd	17
c	Glycolic acid	28	Traces	Traces	Traces
d	(*S*)-Lactic acid	50	nd	33.8^b^	
e	Capryloyl glycine	34	nd	20	9
f	*p*-Coumaric acid	36	nd	nd	nd
g	Dihydroferulic acid	75	nd	nd	nd
h	(*R*,*S*)-Mandelic acid	72	nd	nd	nd

a nd: not detected.

b Mixture of diesters.

In the case of aliphatic acids, octanoic and lauric acids (Table 1, entries a and b), both monoester 3 and diesters 4–6 were produced indicating that the lipase was able to esterify both the primary hydroxyl group of the hexanediol part and some secondary hydroxyl groups from the xylose moiety (Table 1). Several studies into the synthesis of sugar esters have reported that, as with most lipases, lipase N435® preferentially catalyzes the acylation of primary hydroxyl groups, although acylation can also occur on secondary hydroxyl groups.^40^ For example, in the case of the transesterification of pentoses (d-xylose and l-arabinose), with lipase N435® in the presence of vinyl laurate, furanose series esters are mainly synthesized thanks to the priority reaction of the primary alcohol function of the monosaccharide.^34^ Using 5 equivalents of carboxylic acids, in both cases, esterification reactions led to the monoester 3a-b as the major product (40–45% yield, Table 1). For diesters 4 and 6, in both cases, the second acylation reaction occurred preferentially on the hydroxyl group in position 4 of the xyloside (6a, 21%; 6b, 17%) compared to the hydroxyl group in position 2 (4a, 11%; 4b, 9%). Diesters 4–6 are supposed to be formed from monoester 3.

Then, to evaluate the selectivity and regioselectivity of the esterification reaction with aliphatic carboxylic acids, the role of the acyl donor/acceptor ratio was investigated for the reaction of xyloside 1 with octanoic acid (Table 2). Results indicated that a slight excess of acyl donor promotes the synthesis of monoesters whereas an increased concentration drives the synthesis towards diesters. With the 1 : 2 ratio, monoester 3a was isolated in 54% yield. The diesters 6a and 4a represented 24% of the mixture obtained with a slight excess for the second esterification on the *O*-4 of the xyloside (6a, 14%) compared to *O*-2 (4a, 10%). When the amount of donor increases (ratio 1 : 5), the percentage of diesters increases significantly (32%), with a clear majority for 6a (21%) compared to 4a (11%), even if monoester 3a (40%) remains the major product. With the 1 : 10 ratio, the yield of monoester 3a continues to fall (33%) and the 4a–6a diesters become the main products of this esterification reaction (41%). The ratio of diesters 4a–6a increases while the percentage of diester 4a remains almost constant indicating a clear regioselectivity for the hydroxyl group in position 4 of the xyloside. Such regioselectivity for an enzymatic esterification or transesterification using a polymer-supported lipase at the 4–OH group was previously described from β-xylopyranosides, especially in polar solvents. This OH–4 group does not have the highest chemical reactivity, but studies have suggested a better availability for a lipase-catalyzed reaction due to steric effects. A few years ago, the enzymatic synthesis of XOs esters (xylobiose to xylotetraose) was described under similar reaction conditions with complete regioselectivity on the hydroxyl group in the 4′-position of the non-reducing end of the xylo-oligosaccharide.^35^ The positive effect of decreasing the acceptor/donor ratio was also observed during the esterification of chlorogenic acid by the lipase N435® in anhydrous 2M2B solvent.^42^ The regioselectivity of the reaction was affected by the acceptor/donor molar ratio as increasing the amount of palmitic acid reduced the proportion of 4-*O*-acylation from 90% to 68%. Increasing the amount of palmitic acid may enhance the hydrophobicity of the medium that would alter the regioselectivity of the reaction.^42^

**Table 2:** Effect of the acyl acceptor/donor ratio on the regioselectivity of the esterification reaction of xyloside 1 with octanoic acid using N435® lipase

	Yields (%)		
	Ratio 1 : 2	Ratio 1 : 5	Ratio 1 : 10
Monoester 3a	54	40	33
Diester *O*-2 4a	10	11	12
Diester *O*-4 6a	14	21	29

With the aliphatic α-hydroxy acids, glycolic and lactic acids (Table 1, entries c and d), the results were somewhat different. Esterification of AHAs is generally difficult because these compounds can act as both acyl donor and nucleophile in competitive reactions and can undergo self-esterification to form polyesters and lactones at high temperature and poorly hydrated media.^43^ Only few studies on the enzymatic acylation of glycolic acid and lactic acid have been reported in the literature, mainly using primary aliphatic alcohols. From *et al.* reported that lactic acid was unable to act as a nucleophile in a reaction catalyzed by *Candida antarctica* lipase due to the low reactivity of secondary alcohols in the presence of this enzyme.^44^ Several articles have described the important role of the organic solvent used for this enzymatic acylation reaction in overcoming two crucial problems: the high acidity and polarity of lactic acid.^45–47^ Lipase-catalyzed esterification of lactic acid was also performed in supercritical fluids such as scCO_2_ ^48^ or scCF_3_H^49^ and ionic liquids.^50,51^ With a carbohydrate-based nucleophile, two examples were reported using a propyl glycoside^43^ and a methyl^52^ or a butyl α-glucoside.^53,54^ In the latter case, transesterification between butyl α-glucoside and butyl lactate was carried out in a solvent-free medium under reduced pressure to eliminate butanol and shift the equilibrium position of the reaction towards the synthesis of butyl α-glucoside lactate. The esterification reaction was shown to take place only on the primary hydroxy function of the glucoside without the formation of diesters. The product α-glucoside lactate was proved to be far less irritant than the free lactic acid and could act as a lactic acid carrier in cosmetic applications.

In our case, in 2M2B, using glycolic acid with a primary hydroxy group, as the acyl donor, monoester 3c was obtained with a lower yield (28%), minor diesters being also detected in the reaction mixture. Esterification reaction with l-lactic acid with a less reactive secondary hydroxy group, led mainly to the 3d monoester in 50% yield and to two non-separable diesters in 34% yield. Nevertheless, the structure of these two diesters was assigned unambiguously with a major second esterification in *O*-3 position and a minor one in *O*-4 position. These results suggest that when the acyl donor has a substitution in α-position of the carboxylic acid function, a second esterification is also possible, but with a different orientation of the substrates in the active site of the lipase compared to the aliphatic acids described above, leading to subsequent esterification at a different position of the xylose moiety of monoester 3d.

Interestingly, with a protected amino acid, capryloyl glycine, results obtained were similar with the major formation of the monoester (33.6%) and *O*-3 and *O*-4 diesters (20.0% and 9.1% respectively). This result seems to confirm that when carboxylic acid has a substituent at the α-position with a heteroatom, the orientation of substrates in the active site is modified by steric or stereoelectronic effects compared to aliphatic carboxylic acids.

For aromatic carboxylic acids, *p*-coumaric, dihydroferulic and mandelic acids, the acylation reaction was highly regioselective as only monoesters 3f–h were synthesized through acylation of the primary hydroxyl group from the hexanediol part. This result clearly indicates that these monoesters 3 could not be used as acyl acceptors by the lipase in the reaction conditions. The regioselective synthesis of monoesters was previously reported in the case of esterification catalyzed by *C. Antarctica* lipase with cinnamic acid or *p*-hydroxyphenylacetic acid as acyl donors and commercial *n*-octyl glucoside as acceptor.^55^

Whereas the yields of esterification were high for both mandelic and dihydroferulic acids, the synthesis occurred less efficiently in the case of *p*-coumaric acid. Using racemic mandelic acid, the acylation reaction of xyloside 1 led to monoester 3h with a 72% yield as a mixture of two diastereoisomers. Surprisingly, the ^1^H and ^13^C NMR spectra of the mixture of the two diastereoisomers of monoester 3h were completely superimposable. However, a few examples of diastereoisomers with identical ^1^H and ^13^C NMR data have already been reported in the literature when these diastereoisomers have sufficiently distant stereoclusters within the molecule.^56^ To confirm the presence of two diastereomers in the reaction with the racemic mixture of mandelic acid, esterification of xyloside 1 was carried out with each enantiomer of mandelic acid. The two diastereoisomers of monoester 3h were obtained separately and their ^1^H and ^13^C NMR spectra were found to be identical. Finally, the optical rotations of the two pure diastereoisomers were measured and compared to that of the mixture of 3h ([α]_D_^25^ = −31), [α]_D_^25^ = +13 from the *S*-(+) enantiomer and [α]_D_^25^ = −67 from the *R*-(−) enantiomer, confirming the presence of two diastereomers in the reaction with the racemic mixture.

Previous data from literature indicated that the esterification yield is negatively impacted in the presence of phenolic acids possessing a double bond within their aliphatic chain.^55,57,58^ With unsaturated phenyl propanoic acids, the electrophilic character of the carboxyl group decreases by resonance due the presence of the electron-donating substituents on the aromatic ring.^58^ When comparing the esterification of *p*-coumaric acid and *p*-hydroxyphenylacetic acid, catalyzed by the lipase from *C. antarctica* in the presence of butan-1-ol, Stamatis *et al.* observed that the esterification rate was lower for *p*-coumaric acid indicating that the presence of unsaturation within the aliphatic chain combined with the ring possessing a *p*-hydroxyl group could affect the lipase catalytic efficiency.^55^ In a recent work, the substrate specificity of lipase B from *C. antarctica* was investigated with *in vitro* and *in silico* approaches in the presence of 3-phenylpropionate and cinnamic acid as acyl donors to esterify butanol.^59^ The esterification yield was 90% for butyl 3-phenyl propionate and only 13% for butyl cinnamate. The authors concluded that the rigidity of the carboxylic acids containing a double bond conjugated with the aromatic ring reduced their mobility and rotation within the narrow active site of the lipase and thus hindered the use of these acids as acyl donors compared with acids displaying saturated aliphatic chains.

Finally, enzymatic acylation was carried out under the same conditions with dihydroferulic acid and xyloside 2 containing the hydroxyl chain with 4 carbon atoms. Monoester 3′g was obtained in similar conditions to those with xyloside 1, indicating that the length of the aglycone chain does not influence the outcome of the esterification reaction.

### 2.3. (Trans) esterification reaction of monoesters 3

In a first series of experiments, the enzymatic acylation of monoesters 3a and 3g was investigated. Starting with the octanoic monoester 3a, the esterification reaction with dihydroferulic acid, under the same experimental conditions as above, led to the monoester 3g*via* an acidolysis reaction. Conversely, esterification of monoester 3g with octanoic acid led to the production of the monoester 3a as the major compound, the diesters 4a and 6a also being detected by TLC and MS.

**Scheme 3:**
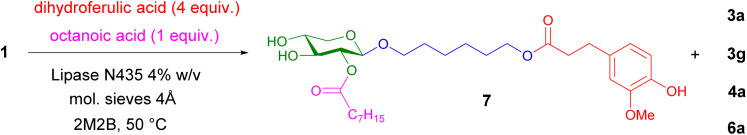
Synthesis of a mixed diester from xyloside 1.

Xyloside 1 was then subjected to an enzymatic esterification reaction with a mixture of octanoic acid (1 equivalent) and dihydroferulic acid (4 equivalent). The reaction gave a mixture of compounds detected by TLC, identified as monoesters 3a and 3g and diesters 4a and 6a. However, a desired diester 7 (Scheme 3) resulting from esterification or acidolysis of the primary hydroxyl group of the xyloside by dihydroferulic acid and esterification by octanoic acid in *O*-2 position of the xylose moiety was isolated in a 5% yield and fully characterized by NMR. Other reactions with different ratios of carboxylic acids did not improve this low yield.

**Scheme 4:**
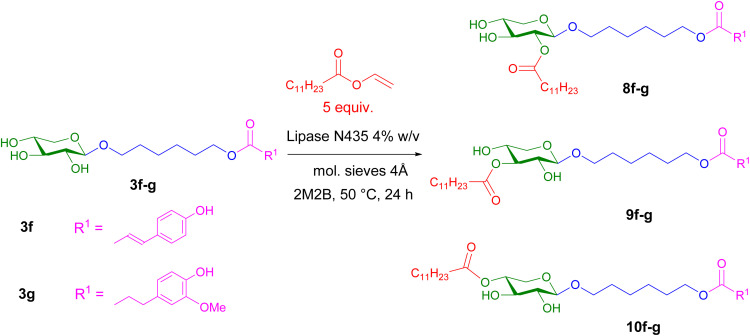
Enzymatic transesterification of xyloside monoesters 3f–g with vinyl laurate.

Finally, the second enzymatic acylation of monoesters was undertaken by a transesterification reaction using vinyl laurate to prevent the acidolysis previously observed. Under modified experimental conditions (monoester/vinyl laurate ratio 1 : 5 solubilized in 2M2B, addition of molecular sieves and immobilized lipase, 24 h, 50 °C), from monoesters 3f–g, diesters 8f–g–10f–g were obtained in about 50% yield after purification on silica gel chromatography (Scheme 4 and Table 3). It is interesting to note that, despite a large excess of vinyl laurate (5 equivalents), approximately 20% of monoesters 3f–g remained unreacted. In both cases, as previously observed when obtaining diesters 4–6, most of the acylation occurred on the OH function in position 4 of the xylosides 3f–g to yield compounds 10f–g, confirming this regioselectivity for esterification reactions with fatty alkyl chains. Small amounts of esterification products 9f–g on OH–3 were also isolated in these cases.

**Table 3:** Enzymatic transesterification of xyloside monoesters 3f–g with vinyl laurate

		Yields (%)		
Entry	Monoester 3	Diester *O*-2 8	Diester *O*-3 9	Diester *O*-4 10
1	3f	8f: 14	9f: 6	10f: 34
2	3g	8g: 10	9g: 6	10g: 33

**Scheme 5:**
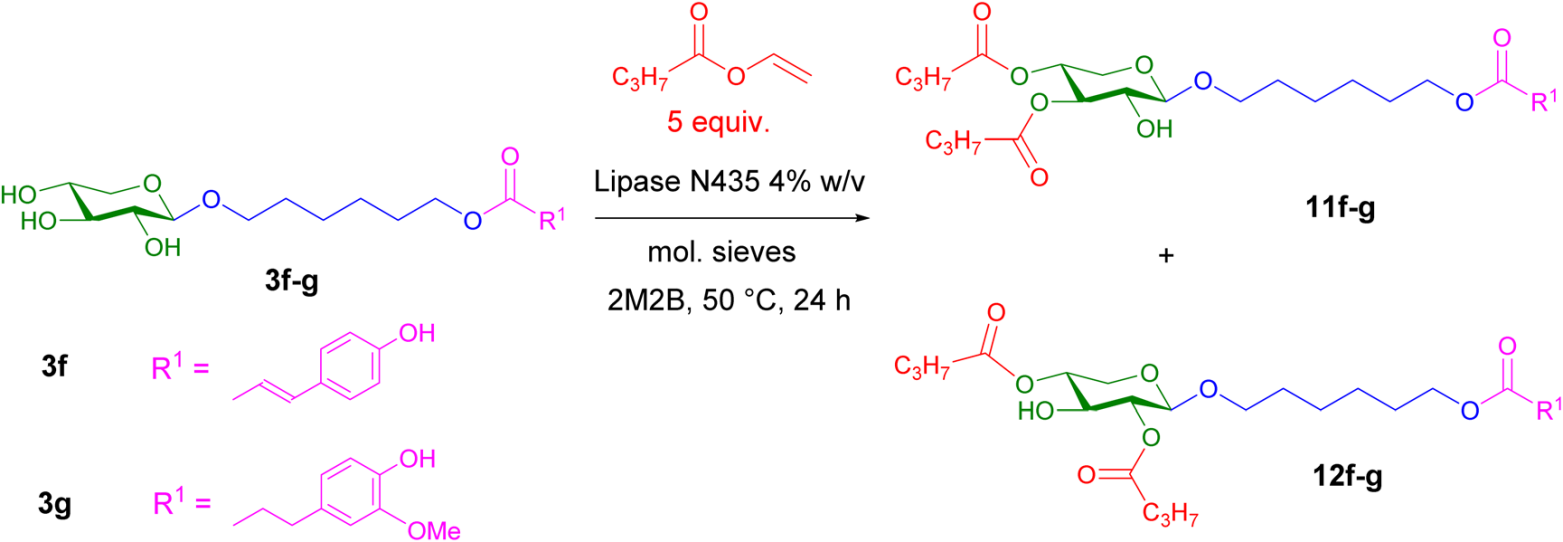
Enzymatic esterification of xyloside monoesters 3f–g with vinyl butyrate.

Using a vinyl ester with a shorter alkyl chain, this esterification reaction of xylosides 3f–g yielded significantly different results (Scheme 5). Under the same experimental conditions as before, the enzymatic acylation of esterified xylosides 3f–g with vinyl butyrate led predominantly to a mixture of triesters 11f–g and 12f–g that could not be separated by chromatography, with yields of 70% and 52%, respectively. An in-depth study of the ^1^H NMR spectra showed that the major triester was attributed to the triester at *O*-3 and *O*-4 11f–g and the minor triester 12f–g to esterification at *O*-2 and *O*-4. The monoesters 3f–g were completely consumed, and only traces of diesters were detected by MS. Other experimental conditions were tested by reducing either the reaction time (6 hours) or the monoester/vinyl butyrate ratio (1 : 1). In both cases, a mixture of remaining monoester, diesters and triesters was observed by TLC and MS.

These results clearly indicate greater reactivity of vinyl butyrate compared to vinyl laurate in these enzymatic acylation reactions catalyzed by immobilized lipase N435®. Similar results have been obtained during the acylation of dihydromyricetin catalyzed by the lipase Lipozyme from *Thermomyces lanuginosus* in the presence of various fatty acid vinyl esters as donors. When the carbon chain length of the fatty acid vinyl ester was increased from C6 to C12, the conversion rate decreased. The conversion was maximal in the case of vinyl butyrate (95.7% after 24 h) and decreased significantly to 10.3% in the case of vinyl laurate after 24 h of reaction.^60^ This could be explained by a generally higher solubility of shorter acyl chains (*e.g*. butyrate) in organic solvents and by less steric hindrance and better accommodation within the active site of the lipase.

## 3. Conclusion

In summary, a three-step enzymatic sequence comprising glycosylation/esterification reactions was developed towards the synthesis of several xyloside esters. In the first step, enzymatic transglycosylation with hexane-1,6-diol from xylans using a xylanase yielded the corresponding β-xyloside with high efficiency. A first acylation reaction of the xyloside using the lipase N435® and various carboxylic acids was then investigated. The esterification reaction proved to be highly regioselective on the primary hydroxyl group of xyloside. However, the outcome of the reaction was found to depend on the nature of the carboxylic acid used. For aromatic carboxylic acids, the acylation reaction was highly regioselective as only monoesters were synthesized through acylation of the primary hydroxyl group. With aliphatic carboxylic acids, mixtures of monoester and diesters with a second esterification on a secondary hydroxyl group of the xylose moiety, mainly at *O*-4 position, were observed. Starting from carboxylic acids with a heteroatom in α-position (α-hydroxy acids or amino acid), mixtures of a major monoester and diesters, mainly at *O*-3 position, was obtained. Finally, starting from *p*-coumaric or dihydroferulic monoesters, a second enzymatic transesterification with vinyl esters (laurate and butyrate) led to diesters or triesters according to the length of the alkyl chain of the vinyl ester.

Molecules comprising various combinations of building blocks were synthesized in order to obtain multifunctional molecules displaying anti-aging property (xyloside, AHA), antioxidant and antimicrobial properties (PA), and moisturizing and cleansing properties (FA). When introduced into cosmetic formulations, the ester bonds within these molecules could be hydrolyzed by enzymes (esterases, lipases) from the skin cells or microbiome, thereby releasing the properties of each of the building blocks. In order to validate their multifunctional nature, the properties of the molecules will be evaluated in a next future.

## Author contributions

C. R., M. M., R. P. R.: conceptualization, funding acquisition, resources, supervision, validation, writing – review & editing; E. J., M. M.: investigation; C. R.: project administration; C. R., M. M., R. P. R., E. J.: writing – original draft.

## Conflicts of interest

There are no conflicts to declare.

## Supplementary Material

RA-016-D5RA09500J-s001

## Data Availability

The data supporting this article have been included as part of the supporting information (SI). Supplementary information: experimental data, materials and methods, NMR and MS spectra can be found in the SI file. See DOI: https://doi.org/10.1039/d5ra09500j.
