# Supplementary material for: Multi-step biocatalytic strategy to produce a library of original xylosides with various ester functions for cosmetic applications
Source: RSC Adv. 2026 Jan 26;16(6):5632–9. doi: 10.1039/d5ra09500j (PMC12833815; doi:10.1039/d5ra09500j)
Supplement: RA-016-D5RA09500J-s001 [file RA-016-D5RA09500J-s001.pdf]

Supplementary Information (SI) for RSC Advances.
This journal is © The Royal Society of Chemistry 2026

Supporting Information

Multi-step biocatalytic strategy to produce a library of original xylosides with various ester functions for cosmetic applications

Emelyne Jolly,[a,b] Murielle Muzard,[a] Richard Plantier-Royon,[a*] Caroline Rémond[b*]

General information ... 2

TLC of transglycosylation reaction of xylans in the presence of hexane-1,6-diol ... 3

TLC of transglycosylation reaction of xylans in the presence of butane-1,4-diol ... 4

Transglycosylation reactions ... 5

Esterification reactions of xyloside **1** ... 7

Transesterification reactions of xyloside monoesters **3f-g** ... 17

^{1}H and ^{13}C NMR spectra of compounds **1/2**, **3a-3h**, **3'g**, **4a/4b**, **6a/6b**, **5e/6e**, **5-6d**, **7**, **8f/8g**, **9f/9g**, **10f/10g**, **11-12f** and **11-12g.** ... 22

HMBC NMR spectra of compounds **1/2**, **3a-3h**, **3'g**, **4a/4b**, **6a/6b**, **5e/6e**, **5-6d**, **7**, **8f/8g**, **9f/9g**, **10f/10g**, **11-12f** and **11-12g**. ... 22

General information

Beechwood xylans were purchased from Roth and consist of approximately 80% xylose units as determined by conventional sugar analysis. CellicCtec® was purchased from Novozyme. Immobilized N435 lipase was purchased from StremChemicals. Hexane-1,6-diol and butane-1,4-diol were obtained from Janssen and Alfa Aesar respectively. Mandelic acid (±), vinyl laurate and butyrate were purchased from Aldrich, glycolic acid and dihydroferulic acid from Fisher Scientific. L-(+)-Lactic, *p*-coumaric, lauric, octanoic acids, molecular sieves 4Å and anhydrous 2M2B were purchased from Sigma.

The reactions were monitored by TLC (silica gel 60 F254) and the plates were revealed by UV (λ = 254 nm) and/or by a stain (orcinol 20% solution in 20% H_2SO_4 or a 3 % *p*-anisaldehyde solution in EtOH). Purifications by chromatography were performed over silica gel (40-63 μm) Kieselgel 60 M.

NMR spectra were recorded on a Bruker Avance neo 500 MHz NMR spectrometer. Chemical shifts δ are given in parts per million (ppm) and coupling constants J in Hertz (Hz). Signal multiplicity is expressed as follows: singlet (s), doublet (d), triplet (t), doublet of doublet (dd), doublet of triplet (dt), triplet of doublet (td), doublet of doublet of doublet (ddd), multiplet (m).

Direct infusion MS analyses were performed on a Waters Acquity UPLC system coupled with a Waters SYNAPT G2-Si High Resolution Mass Spectrometry equipped with electrospray ionization (ESI) source (Waters Corp., Manchester, UK). Mass detections were conducted in positive mode, with the source temperature at 100 °C, capillary voltage and cone voltage were set at 3 kV. Elemental analyses were performed on CHNS Flash EA1112 series Thermo Electron. The specific rotations were measured with an Anton Paar MCP 5100 polarimeter device at 25 °C or 20 °C.

TLC of transglyco[illegible] of hexane-1,6-diol

Eluent: Ethyl acetate/[illegible]

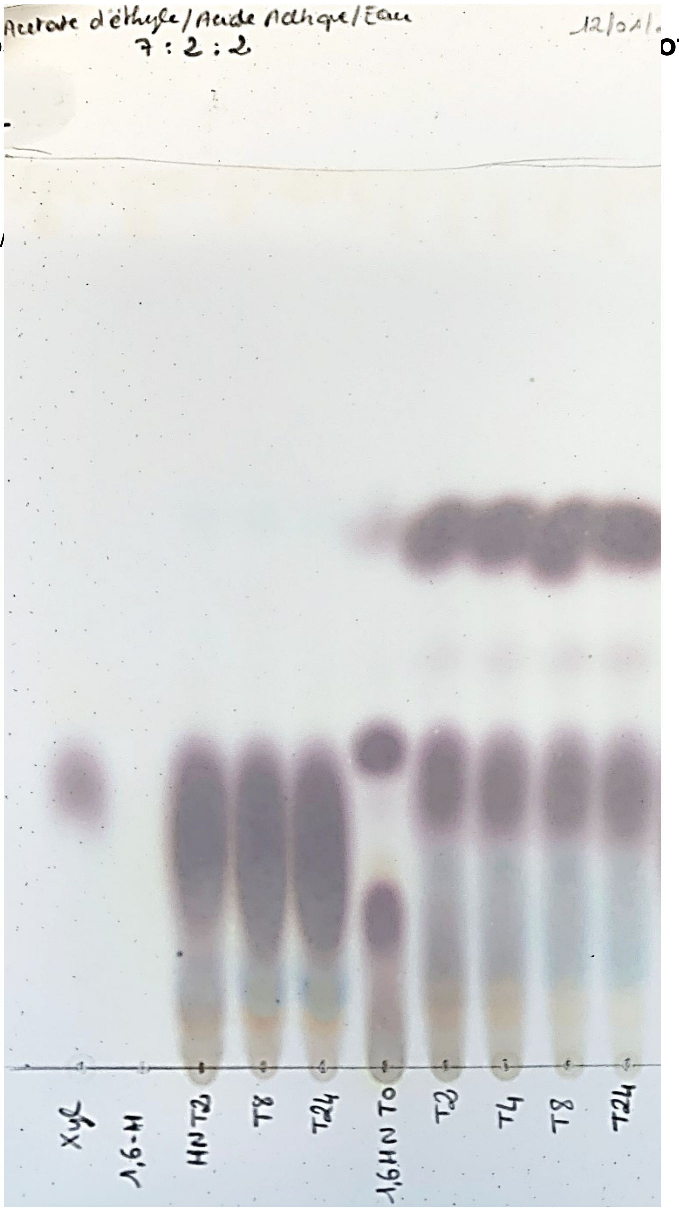

Xylose

T0h T2h T8h

Hydrolyse

T0h T2h T4h T8h T24h

Transglycosylation with hexane 1,6 diol

TLC of transglycosyl[illegible]e of butane-1,4-diol

Eluent: Ethyl acetate/ac[illegible]

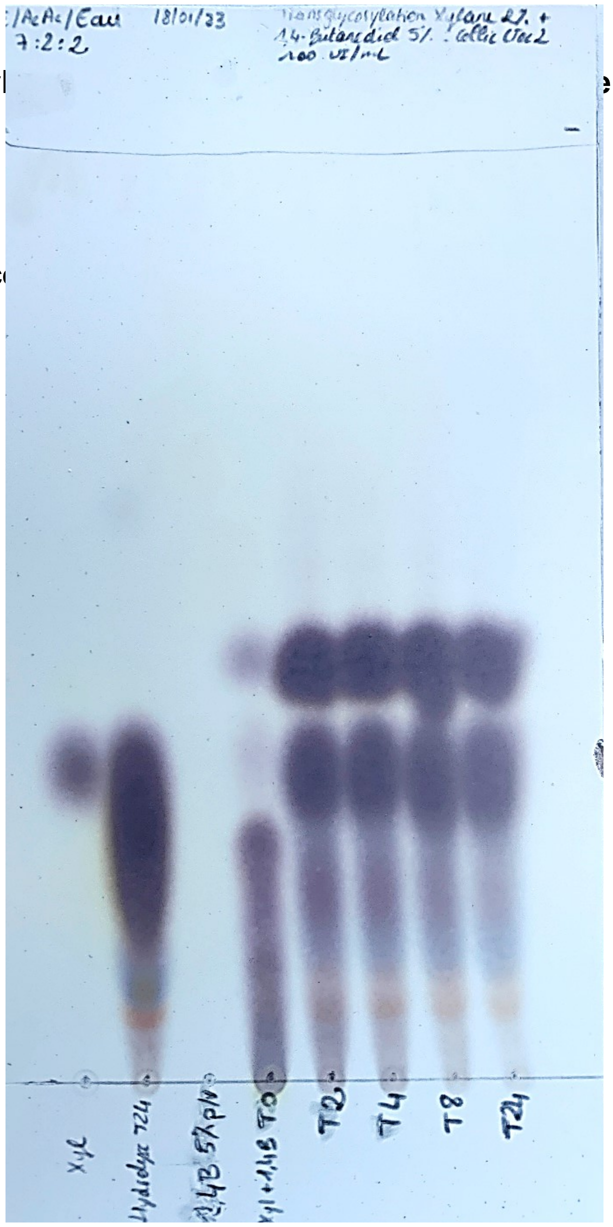

Xylose

Hydrolyse T24h

T0h

T2h

T4h

T8h

T24h

Transglycosylation with butane 1,4 diol

Transglycosylation reactions

Beechwood xylans (2 % w/v) were suspended in water. Diol (5 % w/v) was added, and after homogenization with stirring, CellicCtec® cocktail (100 xylanase UI/mL) was added. The mixture was placed in an oven at 50°C under stirring for 4 h. Reactions were first conducted with 2 mL as final volume. After TLC analysis, scaled-up production was performed with 100 mL volume reaction. At the end of the reaction, the enzyme was denatured by heating (5 min at 100 °C) and the crude material was centrifugated (10 min at 8000 rpm, 6°C). Water was removed under reduced pressure. The crude solid was extracted with 4 x 100 mL of EtOAc/MeOH 90:10. The organic phase was filtered, solvents were removed under reduced pressure and the residue was purified by silica gel chromatography (CH_2Cl_2/MeOH from 100:0 to 80:20).

6-hydroxyhexyl β-D-xylopyranoside ***1***.

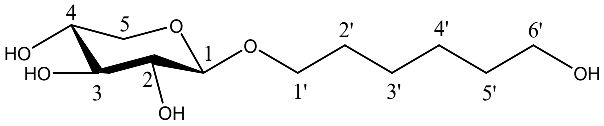

White solid, 2.49 g (53% yield), mp = 105 °C, $[\alpha]_D^{25}$ = -44 (*c* 1.71, MeOH), ^{1}H NMR (CD_3OD, 500 MHz) : δ = 4.21 (d, *J* = 7.6 Hz, 1H, H_1), 3.86 (dd, *J* = 11.5 Hz, *J* = 5.3 Hz, 1H, H_5), 3.83 (dt, *J* = 9.5 Hz, *J* = 6.8 Hz, 1H, $H_{1'}$), 3.52-3.58 (m, 3H, $H_{1'}$ and $2H_{6'}$), 3.49 (ddd, *J* = 10.2 Hz, *J* = 8.9 Hz, *J* = 5.3 Hz, 1H, H_4), 3.31 (t, *J* = 8.9 Hz, 1H, H_3), 3.21 (dd, *J* = 11.5 Hz, *J* = 10.2 Hz, 1H, H_5), 3.17 (dd, *J* = 8.9 Hz, *J* = 7.6 Hz, 1H, H_2), 1.60-1.68 (m, 2H, $H_{2'}$), 1.52-1.60 (m, 2H, $H_{5'}$), 1.36-1.47 (m, 4H, $H_{3'}$ and $H_{4'}$); ^{13}C NMR (CD_3OD, 125 MHz) : δ = 103.7 (C_1), 76.5 (C_3), 73.6 (C_2), 69.8 (C_4), 69.4 ($C_{1'}$), 65.5 (C_5), 61.5 ($C_{6'}$), 32.2 ($C_{5'}$), 29.4 ($C_{2'}$), 25.5 and 25.3 ($C_{3'}$ and $C_{4'}$); HRMS m/z calcd for $C_{11}H_{22}O_6Na$ $[M+Na]^+$ 273.1314, found 273.1317; Elemental analysis calcd (%) for $C_{11}H_{22}O_6$: C 52.79, H 8.86, found : C 52.65, H 9.23.

4-hydroxybutyl β-D-xylopyranoside ***2***.

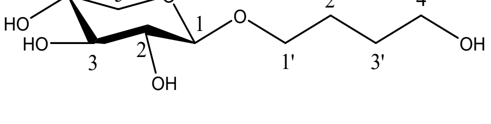

White solid, 1.95 g (46% yield), mp = 112 °C, $[\alpha]_D^{25}$= -49 (*c* 1.92, MeOH), ^{1}H NMR (CD_3OD, 500 MHz) : δ = 4.21 (d, *J* = 7.6 Hz, 1H, H_1), 3.83-3.89 (m, 2H, H_5 and $H_{1'}$), 3.55-3.62 (m, 3H, $H_{1'}$ and $H_{4'}$), 3.49 (ddd, *J* = 10.4 Hz, *J* = 8.9 Hz, *J* = 4.4 Hz, 1H, H_4) ; 3.31 (t, *J* = 8.9 Hz, 1H, H_3), 3.21 (dd, *J* = 11.3 Hz, *J* = 10.4 Hz,1H, H_5), 3.17 (dd, *J* = 8.9 Hz, *J* = 7.6 Hz, 1H, H_2), 1.60-1.73 (m, 4H, $H_{2'}$ and $H_{3'}$); ^{13}C NMR (CD_3OD, 125 MHz) : δ = 103.7 (C_1), 76.5 (C_3), 73.6 (C_2),

69.8 (C_4), 69.2 ($C_{1'}$), 65.5 (C_5), 61.3 ($C_{4'}$), 28.8 and 25.8 ($C_{3'}$ and $C_{2'}$); HRMS m/z calcd for $C_9H_{18}O_6Na$ $[M+Na]^+$ 245.1001, found 245.1000; Elemental analysis calcd (%) for $C_9H_{18}O_6$: C 48.64, H 8.16, found C 48.42, H 7.97.

Esterification reactions of xyloside 1

Xyloside **1** (1 eq) and carboxylic acid (5 eq) were solubilized in 50 mL of anhydrous 2M2B (40 mM for xyloside). Molecular sieves 4 Å (10 % w/v) and immobilized lipase N435 (4% w/v) were added. The reaction mixture was stirred at 50 °C during 72 h.

The liquid phase was removed, centrifugated and solvent was eliminated under reduced pressure. The residue was purified by silica gel chromatography.

*6-O-(octanoyl)hexyl β-D-xylopyranoside **3a**.*

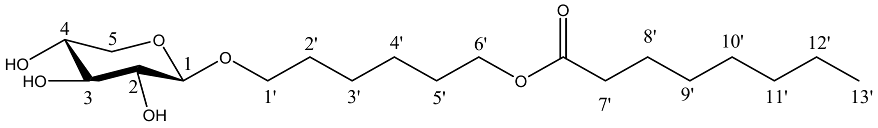

Purified by silica gel chromatography (EtOAc/Petroleum ether 30:70 to 100:0 and EtAOc/MeOH 90:10).

White solid, 300 mg (40% yield), mp = 52 °C, $[\alpha]_D^{25}$ = -34 (*c* 1.76, CH_2Cl_2), ^1H NMR ($CDCl_3$, 500 MHz) : δ = 4.31 (d, *J* = 7.4 Hz, 1H, H_1), 4.07 (t, *J* = 6.7 Hz, 2H, $H_{6'}$), 3.99 (dd, *J* = 12.5 Hz, *J* = 4.8 Hz, 1H, H_5), 3.85 (dt, *J* = 13.6 Hz, *J* = 6.8 Hz, 1H, $H_{1'}$), 3.67-3.75 (m, 1H, H_4), 3.50-3.58 (m, 2H, H_3 and $H_{1'}$), 3.41 (t, *J* = 7.4 Hz, 1H, H_2), 3.31 (dd, *J* = 11.7 Hz, *J* = 9.1 Hz,1H, H_5), 2.30 (t, *J* = 7.5 Hz, 2H, $H_{7'}$), 1.61-1.67 (m, 6H, $H_{2'}$, $H_{5'}$, $H_{8'}$), 1.37-1.41 (m, 4H, $H_{3'}$ and $H_{4'}$), 1.27-1.34 (m, 8H, $H_{9'}$ to $H_{12'}$), 0.90 (t, *J* = 6.8 Hz, 3H, $H_{13'}$); ^{13}C NMR ($CDCl_3$, 125 MHz) : δ = 174.2 (C=O), 102.8 (C_1), 75.4 (C_3), 72.7 (C_2), 69.75 ($C_{1'}$), 69.7 (C_4), 64.8 (C_5), 64.2 ($C_{6'}$), 34.4 ($C_{7'}$), 31.7 ($C_{9'}$), 29.5 ($C_{2'}$ or $C_{5'}$), 29.1 ($C_{10'}$), 28.9 ($C_{11'}$), 28.5 ($C_{2'}$ or $C_{5'}$), 25.6 and 25.6 ($C_{3'}$ and $C_{4'}$), 25.0 ($C_{8'}$), 22.61 ($C_{12'}$), 14.1 ($C_{13'}$); HRMS m/z calcd for $C_{19}H_{36}O_7Na$ $[M+Na]^+$ 399.2359, found 399.2358.

*6-O-(lauroyl)hexyl β-D-xylopyranoside **3b**.*

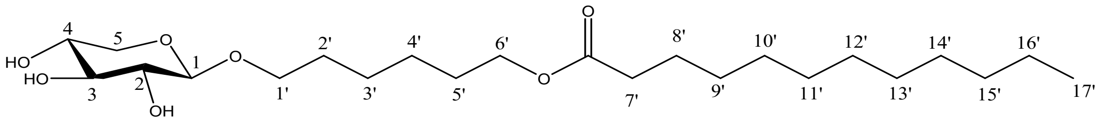

Purified by silica gel chromatography (EtOAc/Petroleum ether 30:70 to 100:0 and EtOAc/MeOH 95:5).

White solid, 380 mg (45% yield), mp = 66 °C, $[\alpha]_D^{25}$ = -27 (*c* 1.85, CH_2Cl_2), ^1H NMR ($CDCl_3$, 500 MHz) : δ = 4.40 (d, *J* = 6.0, 1H, H_1), 4.08 (m, 3H, $1H_5 + 2H_{6'}$), 3.88 (dt, *J* = 9.6 Hz, *J* = 6.6 Hz, 1H, $H_{1'}$), 3.79 (td, *J* = 7.4 Hz, *J* = 4.4 Hz, 1H, H_4), 3.63 (t, *J* = 7.4 Hz, 1H, H_3), 3.53 (dt, *J* = 9.6 Hz, *J* = 6.6 Hz, 1H, $H_{1'}$), 3.47 (dd, *J* = 7.4 Hz, *J* = 6.1 Hz, 1H, H_2), 3.40 (dd, *J* = 11.9 Hz, *J* = 8.0 Hz, 1H, H_5), 2,31 (t, *J* = 7.5 Hz, 2H, $H_{7'}$), 1.60-1.70 (m, 6H : $H_{2'}$, $H_{5'}$, $H_{8'}$), 1.39-1.43 (m, 4H, $H_{3'}$ and $H_{4'}$), 1.26-1.33 (m, 16H, $H_{9'}$ to $H_{16'}$), 0.91 (t, *J* = 6.8 Hz, 3H, $H_{17'}$); ^{13}C NMR ($CDCl_3$,

125 MHz) : δ = 174.1 (C=O), 102.4 (C_1), 74.3 (C_3), 72.2 (C_2), 69.7 (C_4), 69.53 ($C_{1'}$), 64.1 ($C_{6'}$), 64.01 (C_5), 34.4 ($C_{7'}$), 31.9 ($C_{9'}$), 29.6 ($C_{10'}$), 29.5 ($C_{11'}$), 29.4 ($C_{2'}$ or $C_{5'}$), 29.4 ($C_{12'}$), 29.3 ($C_{13'}$), 29.3 ($C_{14'}$), 29.2 ($C_{15'}$), 28.5 ($C_{2'}$ or $C_{5'}$), 25.7 ($C_{3'}$ and $C_{4'}$), 25.0 ($C_{8'}$), 22.7 ($C_{16'}$), 14.1 ($C_{17'}$); HRMS m/z calcd for $C_{23}H_{44}O_7Na$ $[M+Na]^+$ 455.2985, found 455.2982.

6-O-(2-hydroxyacetyl)hexyl β-D-xylopyranoside ***3c.***

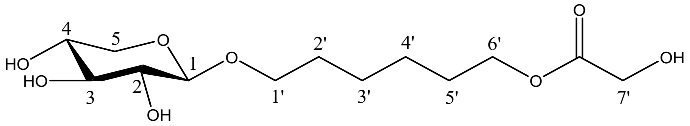

Purified by silica gel chromatography (EtOAc/MeOH 95:5 to 90:10).
White solid, 169 mg (28% yield), mp = 63 °C, $[\alpha]_D^{25}$ = -33 (*c* 1.82, H_2O), ^1H NMR (D_2O, 500 MHz) : δ = 4.31 (d, *J* = 7.9 Hz,1H, H_1), 4.13 (s, 1H, $H_{7'}$), 4.12 (t, *J* = 4.7 Hz, 2H, $H_{6'}$), 3,85 (dd, *J* = 11.4 Hz, *J* = 5.5 Hz, 1H, H_5), 3.77 (dt, *J* = 13.6 Hz, *J* = 6.8 Hz, 1H, $H_{1'}$), 3.58 (dt, *J* = 13.4 Hz, *J* = 6.7 Hz, 1H, $H_{1'}$), 3.49-3.57 (m, 1H, H_4), 3.34 (t, *J* = 9.3 Hz, 1H, H_3), 3.22 (t, *J* = 11.4 Hz, 1H, H_5), 3.15 (dd, *J* = 9.3 Hz, *J* = 7.9 Hz, 1H, H_2), 1.50-1.64 (m, 4H, $H_{2'}$ and $H_{5'}$), 1.27-1.33 (m, 4H, $H_{3'}$ and $H_{4'}$); ^{13}C NMR (D_2O, 125 MHz) : δ = 174.6 (C=O), 102.9 (C_1), 75.8 (C_3), 73.0 (C_2), 70.6 ($C_{1'}$), 69.2 (C_4), 65.9 ($C_{6'}$), 65.8 (C_5), 59.6 ($C_{7'}$), 28.6 ($C_{2'}$), 27.6 ($C_{5'}$), 24.7 ($C_{3'}$), 24.6 ($C_{4'}$); HRMS m/z calcd for $C_{13}H_{24}O_8Na$ $[M+Na]^+$ 331.1369, found 331.1367.

6-O-(2-hydroxypropanoyl)hexyl β-D-xylopyranoside ***3d.***

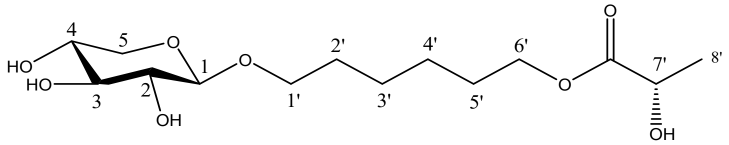

Purified by silica gel chromatography (EtOAc/MeOH 95:5 to 90:10).
Liquid, 323 mg (50% yield), $[\alpha]_D^{25}$ = -38 (*c* 2.19, MeOH), ^1H NMR (CD_3OD, 500 MHz) : δ = 4.26 (q, *J* = 6,9 Hz, 1H, $H_{7'}$), 4.20 (d, *J* = 7.6 Hz, 1H, H_1), 4.12-4.19 (m, 2H, $H_{6'}$), 3.80-3.88 (m, 2H, H_5 and $H_{1'}$), 3.55 (dt, *J* = 13.6 Hz, *J* = 6.5 Hz, 1H, $H_{1'}$), 3.49 (ddd, *J* = 14.2 Hz, *J* = 8.9 Hz, *J* = 5.6 Hz, 1H, H_4), 3.29-3.34 (m, 1H, H_3), 3.14-3.23 (m, 2H, H_2 and H_5), 1.61-1.72 (m, 4H, $H_{2'}$ and $H_{5'}$), 1.40-1.46 (m, 4H, $H_{3'}$ and $H_{4'}$), 1.39 (d, *J* = 6.9 Hz, 3H, $H_{8'}$); ^{13}C NMR (CD_3OD, 125 MHz) : δ = 175.1 (C=O), 103.7 (C_1), 76.52 (C_3), 73.6 (C_2), 69.8 (C_4), 69.3 ($C_{1'}$), 66.5 ($C_{7'}$), 65.5 ($C_{6'}$), 29.25 ($C_{2'}$), 28.3 ($C_{5'}$), 25.3 and 25.3 ($C_{3'}$ and $C_{4'}$), 19.2 ($C_{8'}$); HRMS m/z calcd for $C_{14}H_{26}O_8Na$ $[M+Na]^+$ 345.1525, found 345.1525.

6-O-((N-octanoyl)-2-aminoacetyl)hexyl β-D-xylopyranoside ***3e.***

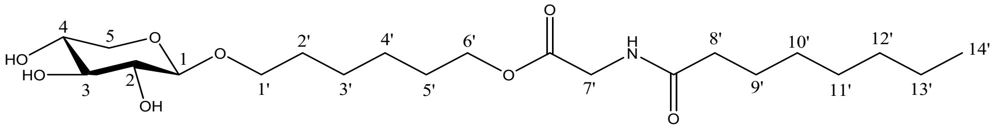

Purified by silica gel chromatography (CH_2Cl_2/MeOH 95:5 to 90:10).

White solid, 291 mg (34% yield), mp = 96 °C, $[\alpha]_D^{25}$ = -22 (c 1.86, MeOH), ^{1}H NMR (CD_3OD, 500 MHz) : δ = 4.20 (d, *J* = 7.6 Hz, 1H, H_1), 4.15 (t, *J* = 6.6 Hz, 2H, $H_{6'}$), 3.95 (s, 2H, $H_{7'}$), 3.80-3.90 (m, 2H, H_5 and $H_{1'}$), 3.55 (dt, *J* = 13.1 Hz, *J* = 6.5 Hz, 1H, $H_{1'}$), 3.48 (ddd, *J* = 14.2 Hz, *J* = 8.9 Hz, *J* = 5.4 Hz, 1H, H_4), 3.31 (t, *J* = 8.9 Hz, 1H, H_3), 3.14-3.23 (m, 2H, H_2 and H_5), 2.26 (t, *J* = 7.4 Hz, 2H, $H_{8'}$), 1.61-1.71 (m, 6H, $H_{2'}$, $H_{5'}$ and $H_{9'}$), 1.40-1.46 (m, 4H, $H_{3'}$ and $H_{4'}$), 1.30-1.39 (m, 8H, $H_{10'}$ to $H_{13'}$), 0.92 (m, 3H, $H_{14'}$); ^{13}C NMR (CD_3OD, 125 MHz) : δ = 175.4 (C=O), 170.1 (C=O), 103.8 (C_1), 76.5 (C_3), 73.6 (C_2), 69.8 (C_4), 69.3 ($C_{1'}$), 65.6 ($C_{6'}$), 64.9 (C_5), 40.6 ($C_{7'}$), 35.4 ($C_{8'}$), 31.5, 28.8, 28.8 and 22.3 ($C_{10'}$ to $C_{13'}$), 29.3 ($C_{2'}$), 28.3 ($C_{5'}$), 25.5 ($C_{9'}$), 25.3 and 25.3 ($C_{3'}$ and $C_{4'}$), 13.0 ($C_{14'}$); HRMS m/z calcd for $C_{21}H_{39}NO_8Na$ $[M+Na]^+$ 456.2573, found 456.2574.

6-O-((E)-3-(p-hydroxyphenyl)prop-2-enoyl)hexyl β-D-xylopyranoside ***3f.***

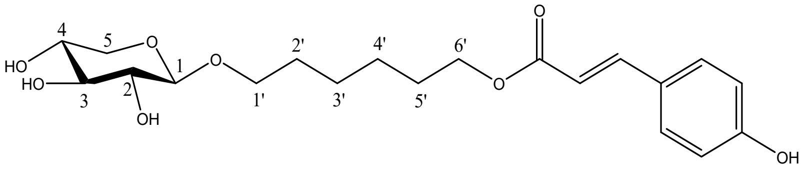

Purified by silica gel chromatography (EtOAc/MeOH 95:5 to 90:10).

White solid, 283 mg (36% yield), mp = 89 °C, $[\alpha]_D^{25}$ = -27 (*c* 1.76, MeOH), ^{1}H NMR (CD_3OD, 500 MHz) : δ = 7.62 (d, *J* = 15.9 Hz, 1H, $H_{7'}$), 7.48 (d, *J* = 8.6 Hz, 2H, ArH), 6.83 (d, *J* = 8.6 Hz, 2H, ArH), 6.35 (d, *J* = 15.9 Hz, 1H, $H_{8'}$), 4.21 (d, *J* = 7.6 Hz, 1H, H_1), 4.20 (t, *J* = 6.6 Hz, 2H, $H_{6'}$), 3.81-3.88 (m, 2H, H_5 and $H_{1'}$), 3.56 (dt, *J* = 13.1 Hz, *J* = 6.5 Hz, 1H, $H_{1'}$), 3.49 (ddd, *J* = 14.2 Hz, *J* = 8.9 Hz, *J* = 5.4 Hz, 1H, H_4), 3.29-3.33 (m, 1H, H_3), 3.15-3.23 (m, 2H, H_2 and H_5), 1.63-1.77 (m, 4H, $H_{2'}$ and $H_{5'}$), 1.40-1.50 (m, 4H, $H_{3'}$ and $H_{4'}$), ^{13}C NMR (CD_3OD, 125 MHz) : δ = 168.0 (C=O), 159.9 (Ar), 145.1 ($C_{7'}$), 129.7 (Ar), 129.6 (Ar), 125.8 (Ar), 115.4 (Ar), 115.4 (Ar), 113.9 ($C_{8'}$), 103.8 (C_1), 76.5 (C_3), 73.6 (C_2), 69.9 (C_4), 69.3 ($C_{1'}$), 65.6 (C_5), 64.1 ($C_{6'}$), 29.3 ($C_{5'}$), 28.4 ($C_{2'}$), 25.5 ($C_{3'}$), 25.4 ($C_{4'}$); HRMS m/z calcd for $C_{20}H_{28}O_8Na$ $[M+Na]^+$ 419.1682, found 419.1682.

6-O-(3-(p-hydroxyl-m-methoxyphenyl)propanoyl)hexyl β-D-xylopyranoside ***3g*.**

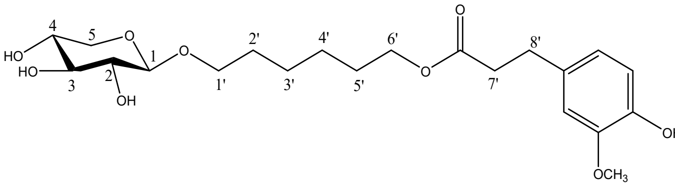

Purified by silica gel chromatography (CH_2Cl_2/MeOH 90:10).

Liquid, 642 mg (75% yield), $[\alpha]_D^{25}$ = -25 (*c* 2.29, $CHCl_3$), ^{1}H NMR ($CDCl_3$, 500 MHz) : δ = 6.84 (d, *J* = 7.9 Hz, 1H, ArH), 6.69-6.74 (m, 2H, ArH), 4.31 (d, *J* = 6.6 Hz, 1H, H_1), 4.06 (t, *J* = 6.6 Hz, 2H, $H_{6'}$), 4.00 (dd, *J* = 11.7 Hz, *J* = 4.7 Hz, 1H, H_5), 3.88 (s, 3H, OCH_3), 3.81 (dt, *J* = 13.7 Hz, *J* = 6.8 Hz, 1H, $H_{1'}$), 3.72 (dt, *J* = 13.1 Hz, *J* = 8.2 Hz, 1H, H_4), 3.58 (t, *J* = 8,2 Hz, 1H, H_3), 3.50 (dt, *J* = 13.7 Hz, *J* = 6.8 Hz, 1H, $H_{1'}$), 3.43 (m, 1H, H_2), 3.32 (dd, *J* = 11.7 Hz, *J* = 9.0 Hz, 1H, H_5), 2.90 (t, *J* = 7.5 Hz, 2H, HC=CH), 2.61 (t, *J* = 7.5 Hz, 2H, HC=CH), 1.59 (hex, *J* = 7.3 Hz, 4H, $H_{2'}$ and $H_{5'}$), 1.23-1.37 (m, 4H, $H_{3'}$ and $H_{4'}$); ^{13}C NMR ($CDCl_3$, 125 MHz) : δ = 173.3 (C═O), 146.6 (Ar), 143.7 (Ar), 132.5 (Ar), 120.9 (Ar), 114.6 (Ar), 111.2 (Ar), 102.8 (C_1), 75.5 (C_3), 72.8 (C_2), 69.8 (C_4), 69.7 ($C_{1'}$), 64.8 (C_5), 64.4 ($C_{6'}$), 55.9 (OCH_3), 36.3 ($C_{7'}$), 30.8 ($C_{8'}$), 29.4 ($C_{5'}$), 28.5 ($C_{2'}$), 25.6 ($C_{3'}$), 25.6 ($C_{4'}$); HRMS m/z calcd for $C_{21}H_{32}O_9Na$ $[M+Na]^+$ 451.1944, found 451.1945.

6-O-((2-hydroxy-2-phenyl)acetyl)hexyl β-D-xylopyranoside ***3h*.**

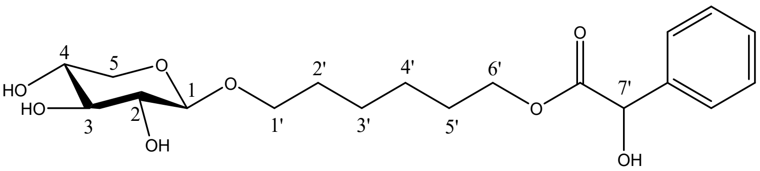

Purified by silica gel chromatography (CH_2Cl_2/MeOH 95:5 to 90:10).

Liquid, 557 mg (72% yield), $[\alpha]_D^{25}$ = -31 (*c* 2.12, MeOH), ^{1}H NMR (CD_3OD, 500 MHz) : δ = 7.44-7.48 (m, 2H, ArH), 7.31-7.40 (m, 3H, ArH), 5.19 (s, 1H, $H_{7'}$), 4.07-4.20 (m, 3H, H_1 and $H_{6'}$), 3.86 (dd, *J* = 11.4 Hz, *J* = 5.3 Hz, 1H, H_5), 3.74-3.80 (m, 1H, $H_{1'}$), 3.46-3.53 (m, 2H, $H_{1'}$ and H_4), 3.29-3.34 (m, 1H, H_3), 3.14-3.23 (m, H_2 and H_5), 1.51-1.61 (m, 4H, $H_{2'}$ and $H_{5'}$), 1.19-1.36 (m, 4H, $H_{3'}$ and $H_{4'}$) ; ^{13}C NMR (CD_3OD, 125 MHz) : δ = 173.2 (C═O), 139.2 (Ar), 128.1 (2 Ar), 128.0 (Ar), 126,5 (2 Ar), 103.7 (C_1), 76.5 (C_3), 73.6 (C_2), 73.0 ($C_{7'}$), 69.9 (C_4), 69.3 ($C_{1'}$), 65.5 (C_5), 64.8 ($C_{6'}$), 29.2 and 28.1 ($C_{2'}$ and $C_{5'}$), 25.2 ($C_{3'}$ and $C_{4'}$) ; HRMS m/z calcd for $C_{19}H_{28}O_8Na$ $[M+Na]^+$ 407.1682, found 407.1685.

6-O-(3-(p-hydroxy-m-methoxyphenyl)propanoyl)butyl β-D-xylopyranoside ***3'g***.

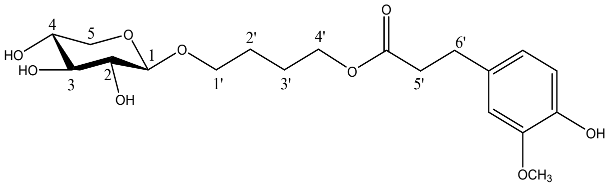

Purified by silica gel chromatography (CH_2Cl_2/MeOH 90:10).

Liquid, 563 mg (70% yield), $[\alpha]_D^{25}$ = -23 (*c* 1.84, $CHCl_3$), ^1H NMR ($CDCl_3$, 500 MHz) : δ = 6.84 (d, *J* = 7.9 Hz, 1H, ArH), 6.67-6.74 (m, 2H, ArH), 4.28 (d, *J* = 6.9 Hz, 1H, H_1), 4.07-4.12 (m, 2H, $H_{4'}$), 3.98 (dd, *J* = 11.7 Hz, *J* = 5.0 Hz, 1H, H_5), 3.81-3.89 (m, 4H, OCH_3 and $H_{1'}$), 3.71 (m, 1H, H_4), 3.56 (m, 1H, H_3), 3.48 (dt, *J* = 12.8 Hz, *J* = 6.3 Hz, 1H, $H_{1'}$), 3.40 (dd, *J* = 8.3 Hz, *J* = 6.9 Hz, 1H, H_2), 3.29 (dd, *J* = 11.7 Hz, *J* = 9.3 Hz, 1H, H_5), 2.88 (t, *J* = 7.6 Hz, 2H, $H_{6'}$), 2.61 (t, *J* = 7.6 Hz, 2H, $H_{5'}$), 1.53-1.73 (m, 4H, $H_{2'}$ and $H_{3'}$); ^{13}C NMR ($CDCl_3$, 125 MHz) : δ = 173.4 (C═O), 146.6 (Ar), 144.0 (Ar), 132.4 (Ar), 120.9 (Ar), 114.6 (Ar), 111.2 (Ar), 102.9 (C_1), 75.6 (C_3), 72.9 (C_2), 69.7 (C_4), 69.3 ($C_{1'}$), 65.0 (C_5), 64.2 ($C_{4'}$), 55.9 (OCH_3), 36.3 ($C_{5'}$), 30.8 ($C_{6'}$), 29.4 ($C_{5'}$), 25,8 and 25,4 ($C_{2'}$ and $C_{3'}$); HRMS m/z calcd for $C_{19}H_{28}O_9Na$ $[M+Na]^+$ 423.1631, found 423.1628.

6-O-(octanoyl)hexyl 2-O-octanoyl-β-D-xylopyranoside ***4a***.

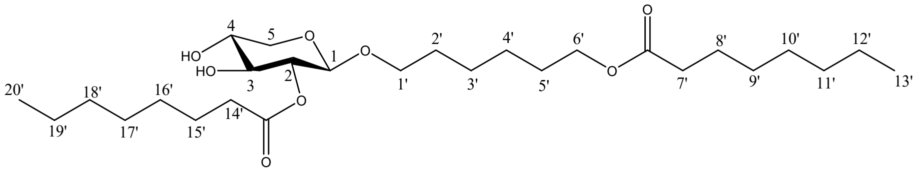

Purified by silica gel chromatography (EtOAc/Petroleum ether 30:70 to 100:0 and EtAOc/MeOH 90:10).

Liquid, 115 mg (11% yield), $[\alpha]_D^{25}$ = -25 (*c* 1.61, $CHCl_3$), ^1H NMR ($CDCl_3$, 500 MHz) : δ = 4.73 (dd, *J* = 8,2 Hz, *J* = 6.7 Hz, 1H, H_2), 4.40 (d, *J* = 6.6 Hz, 1H, H_1), 3.98-4.06 (m, 3H, H_5 and $H_{6'}$), 3.79 (dt, *J* = 13.1 Hz, *J* = 6.6 Hz, 1H, $H_{1'}$), 3.69 (dt, *J* = 12.8 Hz, *J* = 4.6 Hz, 1H, H_4), 3.55 (t, *J* = 8.1 Hz, 1H, H_3), 3.43 (dt, *J* = 13.4 Hz, *J* = 6.6 Hz, 1H, $H_{1'}$), 3.29 (dd, *J* = 11.8 Hz, *J* = 8.8 Hz, 1H, H_5), 2.34 (td, *J* = 11.0 Hz, *J* = 3.3 Hz, 2H, $H_{14'}$), 2.27 (t, *J* = 7.5 Hz, 2H, $H_{7'}$), 1.52-1.65 (m, 8H, $H_{2'}$, $H_{5'}$, $H_{8'}$, $H_{15'}$), 1.22-1.36 (m, 20H, $H_{3'}$, $H_{4'}$, $H_{9'}$ to $H_{12'}$ and $H_{16'}$ to $H_{19'}$), 0.87 (t, *J* = 6.7 Hz, 6H, $H_{13'}$ and $H_{20'}$); ^{13}C NMR ($CDCl_3$, 125 MHz) : δ = 174.1 (C═O), 173.5 (C═O), 100.7 (C_1), 74.2 (C_3), 72.7 (C_2), 69.9 (C_4), 69.4 ($C_{1'}$), 64.4 ($C_{6'}$), 64.2 (C_5), 34.6 ($C_{14'}$), 34.3 ($C_{7'}$), 31.6 ($C_{9'}$ and $C_{16'}$), 29.4, 28.6, 25.0 and 24.9 ($C_{2'}$, $C_{5'}$, $C_{8'}$ and $C_{15'}$), 29.1, 29.0, 28.9, 28.9, 22.6 and 22.6 ($C_{10'}$ to $C_{12'}$ and $C_{17'}$ to $C_{19'}$), 25.7 and 25.6 ($C_{3'}$ and $C_{4'}$), 14.0 ($C_{13'}$ and $C_{20'}$) ; 2D experiment (HMBC): correlations between C═O 174.1 and $H_{6'}$, C═O 173,5 and H_2; HRMS m/z calcd for

$C_{27}H_{50}O_8Na$ [M+Na]$^+$ 525.3403, found 525.3405; Elemental analysis calcd (%) for $C_{27}H_{50}O_8$: C 64.51, H 10.03, found : C 64.15, H 9.72.

6-O-(lauroyl)hexyl 2-O-lauroyl-β-D-xylopyranoside ***4b*.**

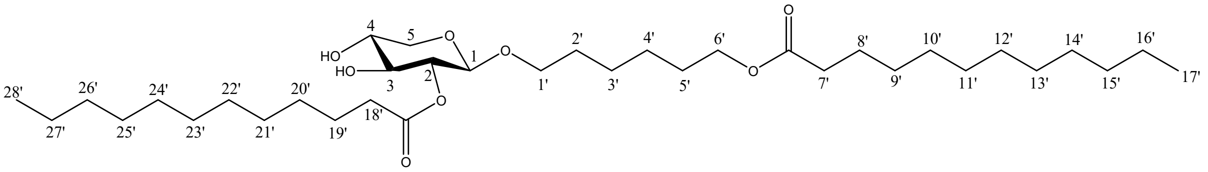

Purified by silica gel chromatography (EtOAc/Petroleum ether 30:70 to 100:0 and EtAOc/MeOH 95:5).

White solid, 116 mg (9% yield), mp = 59 °C, $[\alpha]_D^{25}$ = -19 (*c* 1.87, MeOH), ^{1}H NMR (CD_3OD, 500 MHz) : δ = 4.71 (dd, *J* = 9.5 Hz, *J* = 8.0 Hz, 1H, H_2), 4.36 (d, *J* = 8.0 Hz, 1H, H_1), 4.08 (t, *J* = 6,6 Hz, 2H, $H_{6'}$), 3.90 (dd, *J* = 11.5 Hz, *J* = 5.4 Hz, 1H, H_5), 3.83 (dt, *J* = 12.4 Hz, *J* = 6.2 Hz, 1H, $H_{1'}$), 3.53-3.60 (m, 1H, H_4), 3.43-3.49 (m, 2H, $H_{1'}$ and H_3), 3.23 (dd, *J* = 11.4 Hz, *J* = 10.5 Hz, 1H, H_5), 2.38 (q, *J* = 7.3 Hz, 2H, $H_{18'}$), 2.33 (t, *J* = 7.4 Hz, 2H, $H_{7'}$), 1.54-1.69 (m, 8H, $H_{2'}$, $H_{5'}$, $H_{8'}$ and $H_{19'}$), 1.29-1.42 (m, 36H, $H_{3'}$,$H_{4'}$, $H_{9'}$ to $H_{16'}$ and $H_{20'}$ to $H_{27'}$), 0.93 (t, *J* = 6.8 Hz, 6H, $H_{17'}$ and $H_{28'}$); ^{13}C NMR (CD_3OD, 125 MHz) : δ = 174.2 (C=O), 173.1 (C=O), 101.6 (C_1), 74.7 (C_3), 73.6 (C_2), 69.9 (C_4), 69.0 ($C_{1'}$), 65.7 ($C_{5'}$), 64.0 ($C_{6'}$), 33.8 and 33.8 ($C_{18'}$ and $C_{7'}$), 31.7 and 31.7 ($C_{9'}$ and $C_{20'}$), 29.4, 29.4, 29.3, 29.2, 29.2, 29.1, 29.1, 29.0, 28.8, 24.7 and 22.4 ($C_{10'}$ to $C_{16'}$ and $C_{21'}$ to $C_{27'}$), 25.4 ($C_{3'}$ and $C_{4'}$), 13.1 ($C_{17'}$ and $C_{28'}$) ; 2D experiment (HMBC): correlations between C=O 174.2 and $H_{6'}$, C=O 173.1 and H_2; HRMS m/z calcd for $C_{35}H_{66}O_8Na$ [M+Na]$^+$ 637.4655, found 637.4658; Elemental analysis calcd (%) for $C_{35}H_{66}O_8$: C 68.37, H 10.82, found C 68.51, H 11.26.

6-O-(octanoyl)hexyl 4-O-octanoyl-β-D-xylopyranoside ***6a*.**

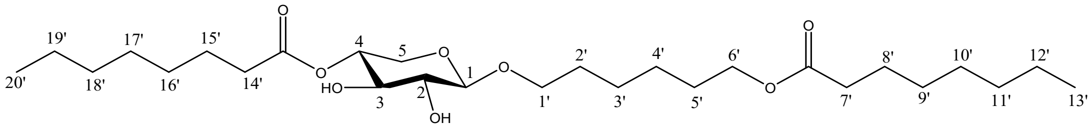

Purified by silica gel chromatography (EtOAc/Petroleum ether 30:70 to 100:0 and EtAOc/MeOH 90:10).

Liquid, 208 mg (21% yield), $[\alpha]_D^{25}$ = -31 (*c* 2.05, MeOH), ^{1}H NMR ($CDCl_3$, 500 MHz) : δ = 4.81 (td, *J* = 7,9 Hz, *J* = 4.7 Hz, 1H, H_4), 4.34 (d, *J* = 6.3 Hz, 1H, H_1), 4.02-4.07 (m, 3H, H_5 and $H_{6'}$), 3.81 (dt, *J* = 13.4 Hz, *J* = 6.7 Hz, 1H, $H_{1'}$), 3.68 (t, *J* = 7.9 Hz, 1H, H_3), 3.50 (dt, *J* = 13.4 Hz, *J* = 6.7 Hz, 1H, $H_{1'}$), 3.45 (dd, *J* = 7.9 Hz, *J* = 6.3 Hz, 1H, H_2), 3.30 (dd, *J* = 11.9 Hz, *J* = 7.9 Hz, 1H, H_5), 2.33 (td, *J* = 11.2 Hz, *J* = 3.6 Hz, 2H, $H_{14'}$), 2.27 (t, *J* = 7.5 Hz, 2H, $H_{7'}$), 1.56-1.66 (m, 8H, H_2, $H_{5'}$, $H_{8'}$, $H_{15'}$), 1.32-1.40 (m, 4H, $H_{3'}$ and $H_{4'}$), 1.20-1.32 (m, 16H, $H_{9'}$ to $H_{12'}$ and $H_{16'}$ to $H_{19'}$), 0.86 (t, *J* = 6.8 Hz, 6H, $H_{13'}$ and $H_{20'}$); ^{13}C NMR ($CDCl_3$, 125 MHz) : δ = 174.1 (C=O),

173.2 (C═O), 102.4 (C_1), 72.6 (C_2), 72.5 (C_3), 71.1 (C_4), 69.6 ($C_{1'}$), 64.1 ($C_{6'}$), 61.7 (C_5), 34.4 and 34.2 ($C_{7'}$ and $C_{14'}$), 31.6 and 31.6 ($C_{9'}$ and $C_{16'}$), 29.4-28.5-25.0 and 24.8 ($C_{2'}$, $C_{5'}$, $C_{8'}$ and $C_{15'}$), 29.1, 29.0, 28.9, 28.9 and 22.6 ($C_{10'}$ to $C_{12'}$ and $C_{17'}$ to $C_{19'}$), 25.6 and 25.6 ($C_{3'}$ and $C_{4'}$), 14.0 ($C_{13'}$ and $C_{20'}$); 2D experiment (HMBC): correlations between C═O 174.1 and $H_{6'}$, C═O 173.2 and H_4; HRMS m/z calcd for $C_{27}H_{50}O_8Na$ $[M+Na]^+$ 525.3403, found 525.3407; Elemental analysis calcd (%) for $C_{27}H_{50}O_8$: C 64.51, H 10.03, found : C 64.66, H 10.42.

6-O-(lauroyl)hexyl 4-O-lauroyl-β-D-xylopyranoside ***6b*.**

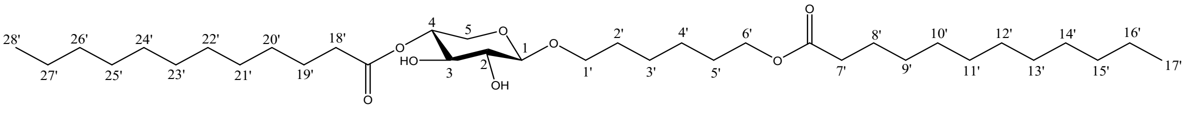

Purified by silica gel chromatography (EtOAc/Petroleum ether 30:70 to 100:0 and EtAOc/MeOH 95:5).

White solid, 205 mg (17% yield), mp = 51 °C, $[\alpha]_D^{25}$ = -26.5 (*c* 1.86, MeOH), ^1H NMR (CD_3OD, 500 MHz) : δ = 4.71 (td, *J* = 9.9 Hz, *J* = 5.6 Hz, 1H, H_4), 4.25 (d, *J* = 7.6 Hz, 1H, H_1), 4.09 (t, *J* = 6.6 Hz, 2H, $H_{6'}$), 3.96 (dd, *J* = 11.4 Hz, *J* = 5.4 Hz, 1H, H_5), 3.83 (dt, *J* = 13.4 Hz, *J* = 6.7 Hz, 1H, $H_{1'}$), 3.53-3.59 (m, 2H, H_3 and $H_{1'}$), 3.22-3.28 (m, 2H, H_2 and H_5), 2.37 (td, *J* = 7.5 Hz, *J* = 4.4 Hz, 2H, $H_{18'}$), 2.33 (t, *J* = 7.4 Hz, 2H, $H_{7'}$), 1.60-1.70 (m, 8H, $H_{2'}$, $H_{5'}$, $H_{8'}$ and $H_{19'}$), 1.40-1.49 (m, 4H, $H_{3'}$ and $H_{4'}$), 1.28-1.37 (m, 32H, $H_{9'}$ to $H_{16'}$ and $H_{20'}$ to $H_{27'}$), 0.92 (t, *J* = 6.7 Hz, 6H, $H_{17'}$ and $H_{28'}$); ^{13}C NMR (CD_3OD, 125 MHz) : δ = 174.3 (C═O), 173.5 (C═O), 103.6 (C_1), 73.7 (C_2), 73.6 (C_3), 71.6 (C_4), 69.3 ($C_{1'}$), 64.1 ($C_{6'}$), 62.3 (C_5), 33.8 and 33.5 ($C_{7'}$ and $C_{18'}$), 31.7 ($C_{9'}$ and $C_{20'}$), 29.3, 29.3, 29.2, 29.1, 29.0, 28.8, 28.8 and 22.4 ($C_{10'}$ to $C_{16'}$ and $C_{21'}$ to $C_{27'}$), 28.3, 24.7 and 24.5 ($C_{2'}$, C_5', $C_{8'}$ and $C_{19'}$), 25.4 and 25.3 ($C_{3'}$ and $C_{4'}$), 13.1 ($C_{17'}$ and $C_{28'}$) ; 2D experiment (HMBC): correlations between C═O 174.3 and $H_{6'}$, C═O 173.5 and H_4; HRMS m/z calcd for $C_{35}H_{66}O_8Na$ $[M+Na]^+$ 637.4655, found 637.4657; Elemental analysis calcd (%) for $C_{35}H_{66}O_8$: C 68.37, H 10.82, found : C 68.23, H 11.26.

6-O-(2-hydroxypropanoyl)hexyl 3-O-(2-hydroxypropanoyl)-β-D-xylopyranoside **5d** (major) and *6-O-(2-hydroxypropanoyl)hexyl 3-O-(2-hydroxypropanoyl)-β-D-xylopyranoside* **6d** (minor).

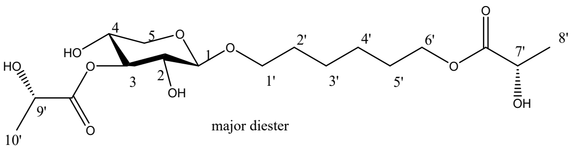

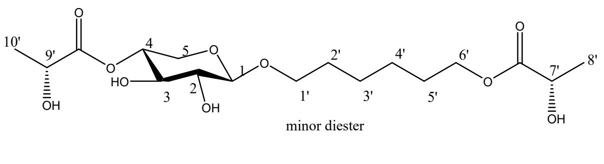

Purified by silica gel chromatography (EtOAc/MeOH 95:5 to 90:10)

Liquid, 266 mg (34% yield for 5d/6d).

5d. ^1H NMR (CD_3OD, 500 MHz) : δ = 4.91 (t, *J* = 9.1 Hz, 1H, H_3), 4.31-4.37 (m, 1H, $H_{9'}$), 4.30 (d, *J* = 7.6 Hz, 1H, H_1) ; 4.20-4.28 (m, 1H, $H_{7'}$) ; 4.11-4.20 (m, 2H, $H_{6'}$) ; 3.92 (dd, *J* = 11.5 Hz,

J = 5.4 Hz, 1H, H_5), 3.81-3.88 (m, 1H, $H_{1'}$), 3.63-3.69 (m, 1H, H_4), 3.54-3.62 (m, 1H, $H_{1'}$), 3.32-3.40 (m, 3H, H_2 and H_5), 1.61-1.73 (m, 4H, $H_{2'}$ and $H_{5'}$), 1.37-1.46 (m, 10H, $H_{3'}$, $H_{4'}$, $H_{8'}$ and $H_{10'}$); ^{13}C NMR (CD_3OD, 125 MHz) : δ = 175.2 (C=O), 174,8 (C=O), 103.6 (C_1), 77.7 (C_3), 71.6 (C_2), 69.4 ($C_{1'}$), 68.0 (C_4), 66.6 and 66.5 ($C_{7'}$ and $C_{9'}$), 65.3 (C_5), 64.6 ($C_{6'}$), 29.2 ($C_{2'}$), 28.3 ($C_{5'}$), 25.3 ($C_{3'}$ and $C_{4'}$), 19.3 ($C_{8'}$ and $C_{10'}$); 2D experiment (HMBC): correlations between C=O 175.2 and $H_{6'}$, C=O 174.8 and H_3.

6d.^1H NMR (CD_3OD, 500 MHz) : δ = 4.73 (td, J = 7.8 Hz, J = 5.5 Hz , 1H, H_4), 4.31-4.37 (m, 1H, $H_{9'}$), 4.30 (d, J = 7.6 Hz, 1H, H_1), 4.20-4.28 (m, 1H, $H_{7'}$), 4.11-4.20 (m, 2H, $H_{6'}$), 3.97 (dd, J = 11.4 Hz, J = 5.4 Hz, 1H, H_5), 3.81-3.88 (m, 1H, $H_{1'}$), 3.54-3.62 (m, 2H, H_3 and $H_{1'}$), 3.24-3.32 (m, 3H, H_2 and H_5), 1.61-1.73 (m, 4H, $H_{2'}$ and $H_{5'}$), 1.37-1.46 (m, 10H, $H_{3'}$, $H_{4'}$, $H_{8'}$ and $H_{10'}$)**;** ^{13}C NMR (CD_3OD, 125 MHz) : δ = 177.1 (C=O), 174,5 (C=O), 103.6 (C_1), 73.6 and 73.5 (C_2 and C_3), 72.2 (C_4), 69.4 ($C_{1'}$), 66.4 and 66.2 ($C_{7'}$ and $C_{9'}$), 64.6 ($C_{6'}$), 62.0 (C_5), 29.2 ($C_{2'}$), 28.3 ($C_{5'}$), 25.3 ($C_{3'}$ and $C_{4'}$), 19.2 ($C_{8'}$ and $C_{10'}$); 2D experiment (HMBC): correlations between C=O 177.1 and $H_{6'}$, C=O 174.5 and H_4.

HRMS m/z calcd for $C_{17}H_{30}O_{10}Na$ $[M+Na]^+$ 417.1737, found 417.1738.

6-O-((N-octanoyl)-2-aminoacetyl)hexyl 3-O-((N-octanoyl)-2-aminoacetyl-β-D-xylopyranoside ***5e****.*

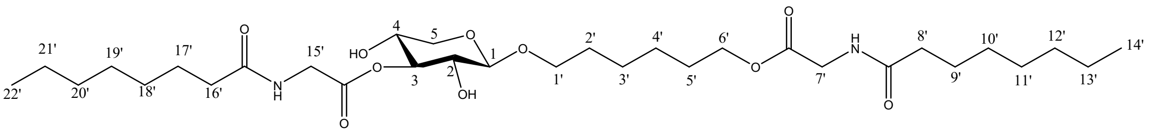

Purified by silica gel chromatography (CH_2Cl_2/MeOH 95:5 to 90:10).

White solid, 246 mg (20% yield), mp = 91 °C, $[\alpha]_D^{25}$ = -25 (*c* 2.05, MeOH), ^1H NMR (CD_3OD, 500 MHz) : δ = 4.90 (t, J = 9.2 Hz, 1H, H_3), 4.30 (d, J = 7.8 Hz, 1H, H_1), 4.15 (t, J = 6.6 Hz, 2H, $H_{6'}$), 4.04 (d, J = 5.6 Hz, 2H, $H_{15'}$), 3.87-3.96 (m, 3H, H_5 and $H_{7'}$), 3.85 (dt, J = 13.2 Hz, J = 6.5 Hz, 1H, $H_{1'}$), 3.62-3.69 (m, 1H, H_4), 3.57 (dt, J = 13.2 Hz, J = 6.5 Hz, 1H, $H_{1'}$), 3.27-3.35 (m, 2H, H_2 and H_5), 2.27 (q, J = 7.9 Hz, 4H, $H_{8'}$ and $H_{16'}$), 1.60-1.71 (m, 8H, $H_{2'}$, $H_{5'}$, $H_{9'}$ and $H_{17'}$), 1.41-1.45 (m, 4H, $H_{3'}$ and $H_{4'}$), 1.30-1.40 (m, 16H, $H_{10'}$ to $H_{13'}$ and $H_{18'}$ to $H_{21'}$), 0.93 (t, J = 6.7 Hz, 6H, $H_{14'}$ and $H_{22'}$); ^{13}C NMR (CD_3OD, 125 MHz) : δ = 175.5 (C=O), 175.4 (C=O), 170.1 (C=O), 169.7 (C=O), 103.4 (C_1), 78.6 (C_3), 71.6 (C_2), 69.4 ($C_{1'}$), 68.0 (C_4), 65.2 (C_5), 64.8 ($C_{6'}$), 40.7 and 40.6 ($C_{7'}$ and $C_{15'}$), 35.4 ($C_{8'}$ and $C_{16'}$), 31.5, 28.8, 28.8 and 22.3 ($C_{10'}$ to $C_{13'}$ and $C_{18'}$ to $C_{21'}$) 29.2, 28.8 and 25.5 ($C_{2'}$, $C_{5'}$, $C_{9'}$ and $C_{17'}$), 25.3 ($C_{3'}$ and $C_{4'}$), 13.0 ($C_{14'}$ and $C_{22'}$); 2D experiment (HMBC): correlations between C=O 170.1 and $H_{6'}$, C=O 169.7 and H_3; HRMS m/z calcd for $C_{31}H_{56}N_2O_{10}Na$ $[M+Na]^+$ 639.3833, found 639.3838; Elemental analysis calcd (%) for $C_{35}H_{56}N_2O_{10}$: C 60.37, H 9.15, N 4.54, found : C 60.25, H 8.97, N 4.65.

6-O-((N-octanoyl)-2-aminoacetyl)hexyl 4-O-((N-octanoyl)-2-aminoacetyl-β-D-xylopyranoside ***6e***.

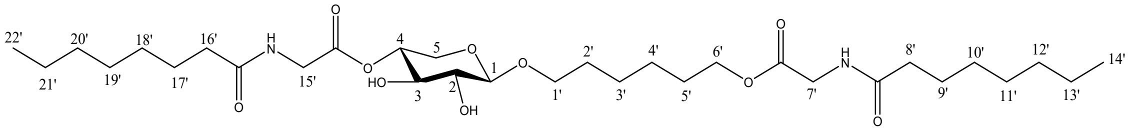

Purified by silica gel chromatography (CH_2Cl_2/MeOH 95:5 to 90:10).

White solid, 111 mg (9% yield), mp = 90 °C, $[\alpha]_D^{25}$ = -26 (*c* 1.58, MeOH), ^{1}H NMR (CD_3OD, 500 MHz) : δ = 4.75 (td, *J* = 9.9 Hz, *J* = 5.6 Hz 1H, H_4), 4.25 (d, *J* = 7.5 Hz, 1H, H_1), 4.15 (t, *J* = 6.6 Hz, 2H, $H_{6'}$), 3.95-4.01 (m, 3H, $H_{5'}$ and $H_{15'}$), 3.93 (s, 2H, $H_{7'}$), 3.83 (dt, *J* = 13.3 Hz, *J* = 6.6 Hz, 1H, $H_{1'}$), 3.53-3.60 (m, 2H, H_3 and $H_{1'}$), 3.24-3.32 (m, 2H, H_2 and H_5), 2.27 (td, *J* = 7.5 Hz, *J* = 1.8 Hz, 4H, $H_{8'}$ and $H_{16'}$), 1.60-1.71 (m, 8H, $H_{2'}$, $H_{5'}$, $H_{9'}$ and $H_{17'}$), 1.40-1.47 (m, 4H, $H_{3'}$ and $H_{4'}$), 1.30-1.40 (m, 16H, $H_{10'}$ to $H_{13'}$ and $H_{18'}$ to $H_{21'}$), 0.93 (t, *J* = 7.1 Hz, 6H, $H_{14'}$ and $H_{22'}$); ^{13}C NMR (CD_3OD, 125 MHz) : δ = 175.5 (C=O), 175.4 (C=O), 170.1 (C=O), 169.5 (C=O),103.6 (C_1), 73.5 (C_3 and C_2), 72.4 (C_4), 69.3 ($C_{1'}$), 64.8 ($C_{6'}$), 62.1 (C_5), 40.6 ($C_{15'}$), 40.6 ($C_{7'}$), 35.4 and 35.3 ($C_{8'}$ and $C_{16'}$), 31.5, 28.8, 28.8 and 22.3 ($C_{10'}$ to $C_{13'}$ and $C_{18'}$ to $C_{21'}$), 29.2, 28.2, 25.5 and 25.5 ($C_{2'}$, $C_{5'}$, $C_{9'}$ and $C_{17'}$), 25.3 and 25.3 ($C_{3'}$ and $C_{4'}$), 13.0 ($C_{14'}$ and $C_{22'}$); 2D experiment (HMBC): correlations between C=O 170.1 and $H_{6'}$, C=O 169.5 and H_4; HRMS m/z calcd for $C_{31}H_{57}N_2O_{10}$ $[M+H]^+$ 617.4013, found 617.4018; Elemental analysis calcd (%) for $C_{35}H_{56}N_2O_{10}$: C 60.37, H 9.15, N 4.54, found : C 60.08, H 9.03, N 4.57.

6-O-(3-(p-hydroxy-m-methoxyphenyl)propanoyl)hexyl 2-O-octanoyl-β-D-xylopyranoside ***7***.

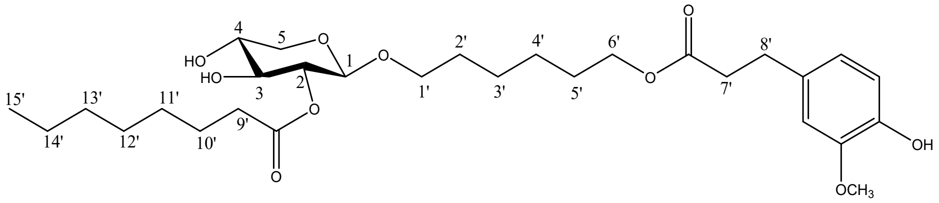

Purified by silica gel chromatography (EtOAc/Petroleum ether 7:3).

Liquid, 13 mg (3% yield), ^{1}H NMR ($CDCl_3$, 500 MHz) : δ = 6.84 (d, *J* = 8.0 Hz, 1H, ArH), 6.68-6.74 (m, 2H, ArH), 4.78 (t, *J* = 5.1 Hz, 1H, H_2), 4.56 (d, *J* = 5.1 Hz, 1H, H_1), 4.03-4.17 (m, 3H, H_5 and $H_{6'}$), 3.89 (s, 3H, OCH_3), 3.81 (dt, *J* = 9.5 Hz, *J* = 6.6 Hz, 1H, $H_{1'}$), 3.64-3.75 (m, 2H, H_4 and H_3), 3.40-3.52 (m, 2H, $H_{1'}$ and H_5), 2.90 (t, *J* = 7.7 Hz, 2H, $H_{8'}$), 2.61 (t, *J* = 7.7 Hz, 2H, $H_{7'}$), 2.39 (t, *J* = 7.5 Hz, 2H, $H_{9'}$), 1.55-1.76 (m, 8H, $H_{2'}$ to $H_{5'}$), 1.24-1.40 (m, 10H, $H_{10'}$ to $H_{14'}$), 0.90 (t, *J* = 6.6 Hz, 3H, $H_{15'}$); ^{13}C NMR ($CDCl_3$, 125 MHz) : δ =173.2 (C=O), 173.1 (C=O), 146.5 (Ar), 144.0 (Ar), 132.5 (Ar), 120.86 (Ar), 114.4 (Ar), 111.0 (Ar), 99.7 (C_1), 72.5 (C_3), 71.8 (C_2), 69.7 (C_4), 69.3 ($C_{1'}$), 64.4 ($C_{6'}$), 63.1 (C_5), 55.9 (OCH_3), 36.3 ($C_{7'}$), 34.3 ($C_{9'}$), 30.8 ($C_{8'}$), 31.6-29.0-28.9-28.6-25.7-24.9-22.6 ($C_{2'}$ to $C_{5'}$ and $C_{10'}$ to $C_{14'}$), 14.1 ($C_{15'}$); 2D experiment (HMBC):

correlations between C═O 173.2 and H_2, C=O 173.1 and $H_{6'}$; HRMS m/z calcd for $C_{29}H_{46}O_{10}$ $[M+Na]^+$ 577.2989, found 577.2990.

Transesterification reactions of xyloside monoesters 3f-g

Xyloside monoester (1 eq) **3f-g** and vinyl laurate (5 eq) were solubilized in 50 mL of anhydrous 2M2B (40 mM for xyloside monoester). Molecular sieves 4Å (10% w/v) and immobilized lipase N435 (4% w/v) were added. The reaction mixture was stirred at 50 °C during 24 h. The liquid phase was removed, centrifugated and solvent was eliminated under reduced pressure. The residue was purified by silica gel chromatography.

6-O-((E)-3-(p-hydroxyphenyl)prop-2-enoyl)hexyl 2-O-lauroyl-β-D-xylopyranoside ***8f***

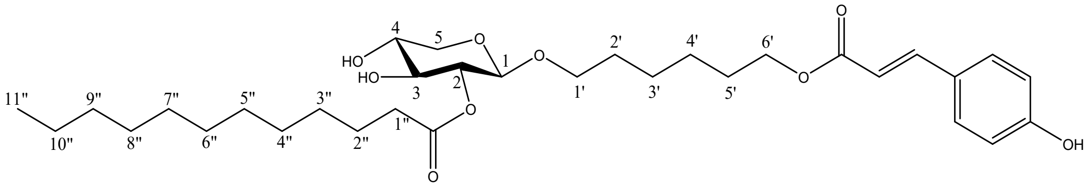

Purified by silica gel chromatography (EtOAc/Petroleum ether 30:70 to 100:0 and EtOAc/MeOH 90:10).

White solid, 64 mg (14% yield), ^{1}H NMR ($CDCl_3$, 500 MHz) : δ = 7.61 (d, *J* = 15.9 Hz, 1H, HC=CH), 7.41 (d, *J* = 8.6 Hz, 2H, ArH), 6.87 (d, *J* = 8.6 Hz, 2H, ArH), 6.28 (d, *J* = 15.9 Hz, 1H, HC=CH), 4.79 (dd, *J* = 6.7 Hz, *J* = 5.2 Hz, 1H, H_2), 4.55 (d, *J* = 5.2 Hz, 1H, H_1), 4.21 (t, *J* = 6.6 Hz, 2H, $H_{6'}$), 4.09 (dd, *J* = 12.0 Hz, *J* = 3.9 Hz, 1H, H_5), 3.83 (dt, *J* = 13.1 Hz, *J* = 6.5 Hz, 1H, $H_{1'}$), 3.73 (t, *J* = 6.7 Hz, 1H, H_4), 3.67 (t, *J* = 6.7 Hz, 1H, H_3), 3.49 (dt, *J* = 13.1 Hz, *J* = 6.5 Hz, 1H, $H_{1'}$), 3.43 (dd, *J* = 12.0 Hz, *J* = 6.7 Hz, 1H, H_5), 2.39 (t, *J* = 7.5 Hz, 2H, $H_{1''}$), 1.60-1.75 (m, 6H, $H_{2'}$, $H_{5'}$ and $H_{2''}$), 1.39-1.46 (m, 4H, $H_{3'}$ and $H_{4'}$), 1.25-1.35 (m, 16H, $H_{3''}$ to $H_{10''}$), 0.89 (t, *J* = 6,7 Hz, 3H, $H_{11''}$); ^{13}C NMR ($CDCl_3$, 125 MHz) : δ = 173.4 (C=O), 167.7 (C=O), 158.0 (Ar), 144.6 (C=C), 130.0 (2 Ar), 127.1 (Ar), 115.9 (2 Ar), 115.5 (C=C), 99.7 (C_1), 72.6 (C_3), 71.9 (C_2), 69.7 (C_4), 69.3 ($C_{1'}$), 64.46 ($C_{6'}$), 63.2 (C_5), 34.3 ($C_{1''}$), 31.9-29.6-29.5-29.4-29.3-29.1-28.6-24.9-22.70 ($C_{2'}$ and $C_{5'}$ and $C_{2''}$ to $C_{10''}$), 25.8 and 25.7 ($C_{3'}$ and $C_{4'}$), 14.1 ($C_{11''}$); 2D experiment (HMBC): correlations between C=O 173.4 and H_2, C=O 167.7 and $H_{6'}$; HRMS m/z calcd for $C_{32}H_{50}O_9Na$ $[M+Na]^+$ 601.3353, found 601.3351.

6-O-((E)-3-(p-hydroxyphenyl)prop-2-enoyl)hexyl 3-O-lauroyl-β-D-xylopyranoside ***9f***

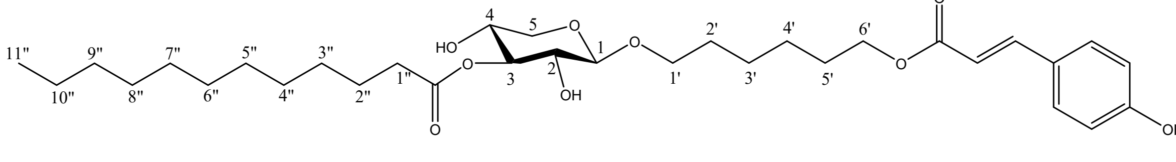

Purified by silica gel chromatography (EtOAc/Petroleum ether 30:70 to 100:0 and EtOAc/MeOH 90:10).

White solid, 30 mg (6% yield), ^{1}H NMR ($CDCl_3$, 500 MHz) : δ = 7.64 (d, J = 15.9 Hz, 1H, HC=CH), 7.44 (d, J = 8.6 Hz, 2H, ArH), 6.86 (d, J = 8.6 Hz, 2H, ArH), 6.31 (d, J = 15.9 Hz, 1H, HC=CH), 4.84 (t, J = 8.6 Hz, 1H, H_3), 4.34 (d, J = 6.9 Hz, 1H, H_1), 4.21 (t, J = 6.6 Hz, 2H, $H_{6'}$), 4.06 (dd, J = 11.7 Hz, J = 5.1 Hz, 1H, H_5), 3.88 (dt, J = 13.3 Hz, J = 6.6 Hz, 1H, $H_{1'}$), 3.79 (td, J = 8.9 Hz, J = 5.1 Hz, 1H, H_4), 3.51-3.58 (m, 2H, H_2 + $H_{1'}$), 3.34 (dd, J = 11.7 Hz, J = 9.4 Hz, 1H, H_5), 2.43 (t, J = 7.0 Hz, 2H, $H_{1''}$), 1.62-1.75 (m, 6H, $H_{2'}$, $H_{5'}$ and $H_{2''}$), 1.42-1.47 (m, 4H, $H_{3'}$ and $H_{4'}$), 1.25-1.36 (m, 16H, $H_{3''}$ to $H_{10''}$), 0.90 (t, J = 6,8 Hz, 3H, $H_{11''}$); ^{13}C NMR ($CDCl_3$, 125 MHz) : δ = 175.7 (C═O), 167.7 (C=O), 158.0 (Ar), 144.6 (C=C), 130.0 (2 Ar), 127.1 (Ar), 115.9 (2 Ar), 115.4 (C=C), 102.9 (C_1), 77.5 (C_3), 71.3 (C_2), 69.8 ($C_{1'}$), 69.0 (C_4), 65.3 (C_5), 64.4 ($C_{6'}$), 34.4 ($C_{1''}$), 31.9-29.6-29.5-29.4-29.3-29.1-28.6-25.7-24.9-22.7 ($C_{2'}$ and $C_{5'}$ and $C_{2''}$ to $C_{10''}$), 25.6 and 25.6 ($C_{3'}$ and $C_{4'}$), 14.1 ($C_{11''}$); 2D experiment (HMBC): correlations between C═O 175.7 and H_3, C=O 167.7 and $H_{6'}$; HRMS m/z calcd for $C_{32}H_{50}O_9Na$ $[M+Na]^+$ 601.3353, found 601.3351.

6-O-((E)-3-(p-hydroxyphenyl)prop-2-enoyl)hexyl 4-O-lauroyl-β-D-xylopyranoside ***10f***.

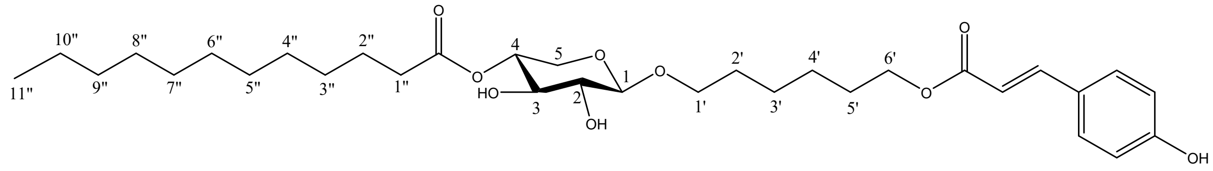

Purified by silica gel chromatography (EtOAc/Petroleum ether 30:70 to 100:0 and EtOAc/MeOH 90:10).

White solid, 161 mg (34% yield), mp = 96°C, $[\alpha]_D^{25}$ = -28.2 (c 1.98, $CHCl_3$), ^{1}H NMR ($CDCl_3$, 500 MHz) : δ = 7.61 (d, J = 15.9 Hz, 1H, C=C), 7.41 (d, J = 8.6 Hz, 2H, ArH), 6.84 (d, J = 8.6 Hz, 2H, ArH), 6.28 (d, J = 15.9 Hz, 1H, C=C), 4.85 (td, J = 7.4 Hz, J = 4.4Hz, 1H, H_4), 4.41 (d, J = 5.8 Hz, 1H, H_1), 4.19 (t, J = 6.6 Hz, 2H, $H_{6'}$), 4.08 (dd, J = 12.1 Hz, J = 4.4 Hz, 1H, H_5), 3.84 (dt, J = 9.6 Hz, J = 6.7 Hz, 1H, $H_{1'}$), 3.73 (t, J = 7.4 Hz, 1H, H_3), 3.47-3.55 (m, 2H, H_2 and $H_{1'}$), 3.36 (dd, J = 12.1 Hz, J = 7.4 Hz, 1H, H_5), 2.35 (td, J = 7.3 Hz, J = 1.4 Hz, 2H, $H_{1''}$), 1.57-1.74 (m, 6H, $H_{2'}$, $H_{5'}$ and $H_{2''}$), 1.38-1.46 (m, 4H, $H_{3'}$ and $H_{4'}$), 1.20-1.33 (m, 16H, $H_{3''}$ to $H_{10''}$), 0.87 (t, J = 6.9 Hz, 3H, $H_{11''}$); ^{13}C NMR ($CDCl_3$, 125 MHz) : δ = 173.4 (C═O), 167.9 (C=O), 158.2 (Ar), 144.7 (C=C), 130.1 (2 Ar), 127.1 (Ar), 116.0 (2 Ar), 115.5 (C=C), 102.3 (C_1), 72.2 (C_2), 72.1 (C_3), 71.2 (C_4), 69.7 ($C_{1'}$), 64.5 ($C_{6'}$), 61.4 (C_5), 34.5 ($C_{1''}$), 32.0-29.7-29.6-29.5-29.5-29.4-29.2-28.7-25.0-22.8 ($C_{2'}$ and $C_{5'}$ and $C_{2''}$ to $C_{10''}$), 25.8 and 25.8 ($C_{3'}$ and $C_{4'}$), 14.2 ($C_{11''}$); 2D experiment (HMBC): correlations between C═O 173.4 and H_4, C=O 167.9 and $H_{6'}$; HRMS m/z calcd for $C_{32}H_{50}O_9Na$ $[M+Na]^+$ 601.3353, found 601.3351.

6-O-(3-(p-hydroxy-m-methoxyphenyl)propanoyl)hexyl 2-O-lauroyl-β-D-xylopyranoside ***8g***.

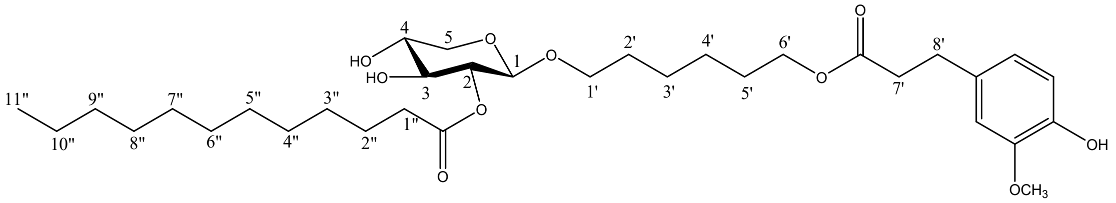

Purified by silica gel chromatography (EtOAc/Petroleum ether 30:70 to 100:0 and EtOAc/MeOH 90:10).

Liquid, 43 mg (10% yield), ^{1}H NMR ($CDCl_3$, 500 MHz) : δ = 6.84 (d, *J* = 8.0 Hz, 1H, ArH), 6.69-6.74 (m, 2H, ArH), 4.78 (dd, *J* = 6.6 Hz, *J* = 5.1 Hz, 1H, H_2), 4.55 (d, *J* = 5.1 Hz, 1H, H_1), 4.04-4.11 (m, 3H, H_5 and $H_{6'}$), 3.89 (s, 3H, OCH_3), 3.81 (dt, *J* = 13.2 Hz, *J* = 6.6 Hz, 1H, $H_{1'}$), 3.69-3.75 (m, 1H, H_4), 3.67 (t, *J* = 7.2 Hz, 1H, H_3), , 3.41-3.50 (m, 2H, $H_{1'}$ and H_5), 2.90 (t, *J* = 7.7 Hz, 2H, $H_{7'}$), 2.61 (t, *J* = 7.6 Hz, 1H, $H_{8'}$), 2.39 (t, *J* = 7.3 Hz, 2H, $H_{1''}$), 1.56-1.69 (m, 6H, $H_{2'}$, $H_{5'}$ and $H_{2''}$), 1.25-1.39 (m, 20H, $H_{3'}$ and $H_{4'}$ and $H_{3''}$ to $H_{10''}$), 0.90 (t, *J* = 6,8 Hz, 3H, $H_{11''}$); ^{13}C NMR ($CDCl_3$, 125 MHz) : δ = 173.3 (C=O), 173.1 (C=O), 146.5 (Ar), 144.0 (Ar), 132.5 (Ar), 120.9 (Ar), 114.4 (Ar), 111.0 (Ar), 99.7 (C_1), 72.5 (C_3), 71.8 (C_2), 69.7 (C_4), 69.3 ($C_{1'}$), 64.4 ($C_{6'}$), 63.1 (C_5), 55.9 (OCH_3), 36.3 ($C_{8'}$), 34.3 ($C_{1''}$), 30.7 ($C_{7'}$), 31.9-29.6-29.5-29.3-29.3-29.1-28.6-25.7-24.9-22.7 ($C_{2'}$ to $C_{5'}$ and $C_{2''}$ to $C_{10''}$), 14.1 ($C_{11''}$); 2D experiment (HMBC): correlations between C=O 173.3 and H_2, C=O 173.1 and $H_{6'}$; HRMS m/z calcd for $C_{33}H_{54}O_{10}Na$ $[M+Na]^+$ 633.3615, found 633.3612.

6-O-(3-(p-hydroxy-m-methoxyphenyl)propanoyl)hexyl 3-O-lauroyl-β-D-xylopyranoside ***9g***.

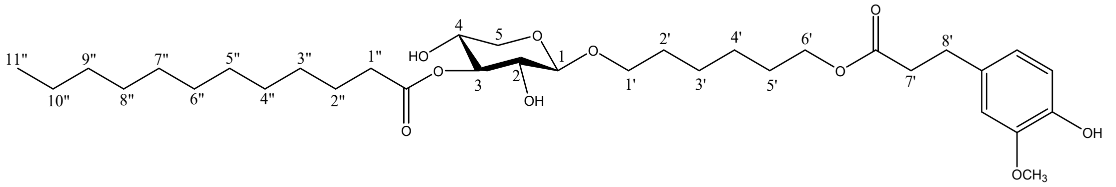

Purified by silica gel chromatography (EtOAc/Petroleum ether 30:70 to 100:0 and EtOAc/MeOH 90:10).

Liquid, 27 mg (6% yield), ^{1}H NMR ($CDCl_3$, 500 MHz) : δ = 6.84 (d, *J* = 7.9 Hz, 1H, ArH), 6.78-6.72 (m, 2H, ArH), 4.83 (t, *J* = 8.7 Hz, 1H, H_3), 4.33 (d, *J* = 7.1 Hz, 1H, H_1), 4.03-4.10 (m, 3H, H_5 and $H_{6'}$), 3.89 (s, 3H, OCH_3), 3.84-3.8 (m, 1H, $H_{1'}$), 3.75-3.83 (m, 1H, H_4), 3.53 (m, 2H, H_2 and $H_{1'}$), , 3.34 (dd, *J* = 11.8 Hz, *J* = 9.5 Hz, 1H, H_5), 2.90 (t, *J* = 7.6 Hz, 2H, $H_{8'}$), 2.61 (t, *J* = 7.6 Hz, 1H, $H_{7'}$), 2.41-2.45 (m, 2H, $H_{1''}$), 1.56-1.71 (m, 6H, $H_{2'}$, $H_{5'}$ and $H_{2''}$), 1.25-1.39 (m, 20H, $H_{3'}$ and $H_{4'}$ and $H_{3''}$ to $H_{10''}$), 0.88-0.92 (m, 3H, $H_{11''}$); ^{13}C NMR ($CDCl_3$, 125 MHz) : δ = 175.6 (C=O), 173.2 (C=O), 146.5 (Ar), 144.0 (Ar), 132.5 (Ar), 120.9 (Ar), 114.4 (Ar), 111.0 (Ar), 103.0 (C_1), 77.7 (C_3), 71.3 (C_2), 69.8 ($C_{1'}$), 69.0 (C_4), 65.4 (C_5), 64.4 ($C_{6'}$), 55.9 (OCH_3), 36.3 ($C_{7'}$), 34.4 ($C_{1''}$), 30.8 ($C_{8'}$), 31.9-29.3-29.3-29.1-28.7-25.6-25.6-22.7 ($C_{3'}$ and $C_{4'}$ and $C_{3''}$ to

$C_{10''}$), 29.5-28.6-24.9 ($C_{2'}$, $C_{5'}$ and $C_{2''}$), 14.1 ($C_{11''}$); 2D experiment (HMBC): correlations between C═O 175.6 and H_3, C═O 173.2 and $H_{6'}$; HRMS m/z calcd for $C_{33}H_{54}O_{10}Na$ $[M+Na]^+$ 633.3615, found 633.3611.

6-O-(3-(p-hydroxy-m-methoxyphenyl)propanoyl)hexyl 4-O-lauroyl-β-D-xylopyranoside ***10g***.

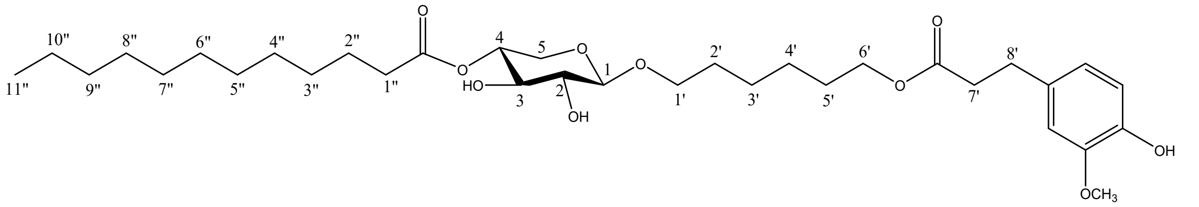

Purified by silica gel chromatography (EtOAc/Petroleum ether 30:70 to 100:0 and EtOAc/MeOH 90:10).

Liquid, 163 mg (34% yield), $[\alpha]_D^{25}$ = -30 (*c* 1.71, $CHCl_3$), ^{1}H NMR ($CDCl_3$, 500 MHz) : δ = 6.84 (d, *J* = 8.0 Hz, 1H, ArH), 6.68-6.74 (m, 2H, ArH), 4.87 (td, *J* = 7.2 Hz, *J* = 4.4Hz, 1H, H_4), 4.45 (d, *J* = 5.7 Hz, 1H, H_1), 4.11 (dd, *J* = 12.1 Hz, *J* = 4.4 Hz, 1H, H_5), 4.08 (t, *J* = 6.7 Hz, 2H, $H_{6'}$), 3.89 (s, 3H, OCH_3), 3.84 (dt, *J* = 9.6 Hz, *J* = 6.7 Hz, 1H, $H_{1'}$), 3.77 (t, *J* = 7.2 Hz, 1H, H_3), 3.49-3.55 (m, 2H, H_2 and $H_{1'}$), 3.39 (dd, *J* = 12.1 Hz, *J* = 7.2 Hz, 1H, H_5), 2.90 (t, *J* = 7.7 Hz, 2H, $H_{8'}$), 2.61 (t, *J* = 7.7 Hz, 2H, $H_{9'}$), 2.35-2.45 (m, 2H, $H_{1''}$), 1.57-1.66 (m, 6H, $H_{2'}$, $H_{5'}$ and $H_{2''}$), 1.22-1.42 (m, 20H, $H_{3'}$ and $H_{4'}$ and $H_{3''}$ to $H_{10''}$), 0.90 (t, *J* = 6,8 Hz, 3H, $H_{11''}$); ^{13}C NMR (CD_3OD, 125 MHz) : δ = 173.1 (2 C═O), 146.5 (Ar), 144.0 (Ar), 132.5 (Ar), 120.9 (Ar), 114.4 (Ar), 111.0 (Ar), 102.2 (C_1), 71.9 (C_2), 71.8 (C_3), 71.1 (C_4), 69.5 ($C_{1'}$), 64.3 ($C_{6'}$), 61.1 (C_5), 55.9 (OCH_3), 36.3 ($C_{7'}$), 34.3 ($C_{1''}$), 31.9 ($C_{3''}$), 30.8 ($C_{8'}$), 29.5 ($C_{5'}$), 29.6-29.4-29.3-29.2-29.1-22.7 ($C_{4''}$ to $C_{10''}$), 28.5 ($C_{2'}$), 25.6 ($C_{3'}$ and $C_{4'}$), 24.9 ($C_{2''}$), 14.1 ($C_{11''}$); 2D experiment (HMBC): correlations between 2 C═O 173.1 and $H_{6'}$ and H_4; HRMS m/z calcd for $C_{33}H_{54}O_{10}Na$ $[M+Na]^+$ 633.3615, found 633.3612.

6-O-((E)-3-(p-hydroxyphenyl)prop-2-enoyl)hexyl 3,4-di-O-butanoyl-β-D-xylopyranoside ***11f***.

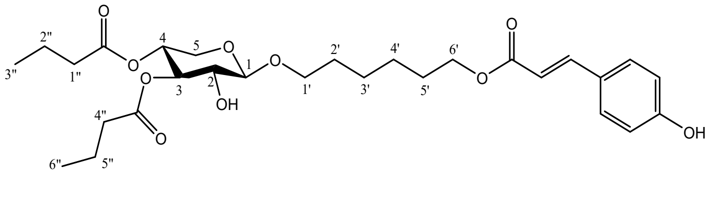

Purified by silica gel chromatography (EtOAc/Petroleum ether 30:70 to 70:30).

Liquid, 296 mg (41% yield for 11f/12f), $[\alpha]_D^{25}$ = -17 (*c* 1.67 $CHCl_3$), ^{1}H NMR ($CDCl_3$, 500 MHz) : δ = 7.64 (d, *J* = 16.0 Hz, 1H, CH=CH), 7.44 (d, *J* = 8.5 Hz, 2H, ArH), 6.87 (d, *J* = 8.5 Hz, 2H, ArH), 6.31 (d, *J* = 16.0 Hz, 1H, CH=CH), 5.14 (t, *J* = 9.0 Hz, 1H, H_3) 4.97 (td, *J* = 9.3 Hz, *J* = 5.3 Hz, 1H, H_4), 4.35 (d, *J* = 7.1 Hz, 1H, H_1), 4.21 (t, *J* = 6.6 Hz, 2H, $H_{6'}$), 4.09 (dd, *J* = 11.6 Hz,

J = 5.3 Hz, 1H, H_5), 3.89 (dt, J = 9.5 Hz, J = 6.6 Hz, 1H, $H_{1'}$), 3.52-3.58 (m, 2H, H_2 and $H_{1'}$), 3.32 (dd, J = 11.6 Hz, J = 5.3 Hz, 1H, H_5), 2.26-2.40 (m, 4H, $H_{1''}$ and $H_{4''}$), 1.57-1.78 (m, 8H, $H_{2'}$, $H_{5'}$,$H_{2''}$ and $H_{5''}$), 1.40-1.47 (m, 4H, $H_{3'}$ and $H_{4'}$), 0.95 (dt, J = 11.5 Hz, J = 7.4 Hz, 6H, $H_{3''}$ and $H_{6''}$); ^{13}C NMR ($CDCl_3$, 125 MHz) : δ = 173.5 (C═O), 172.7 (C=O), 167.7 (C=O), 158.0 (Ar), 144.6 (C=C), 130.0 (2 Ar), 127.1 (Ar), 115.9 (2 Ar), 115.5 (C=C), 103.1 (C_1), 73.2 (C_3), 72.0 (C_2), 70.0 ($C_{1'}$), 68.9 (C_4), 64.4 ($C_{6'}$), 62.5 (C_5), 36.2 and 36.0 ($C_{1''}$ and $C_{4''}$), 29.4 ($C_{2'}$), 28.6 ($C_{5'}$), 25.7 (C4'), 25.6 ($C_{3'}$), 18.4 and 18.3 ($C_{2''}$ and $C_{5''}$), 13.6 ($C_{3''}$ and $C_{6''}$); 2D experiment (HMBC): correlations between C═O 173.5 and H_3, C=O 172.7 and H_4, C=O 167.71 and $H_{6'}$; HRMS m/z calcd for $C_{28}H_{40}O_{10}Na$ $[M+Na]^+$ 559.2519 found 559.2516.

6-O-(3-(p-hydroxyl-m-methoxyphenyl)propanoyl)hexyl 3,4-di-O-butanoyl-β-D-xylopyranoside ***11g***.

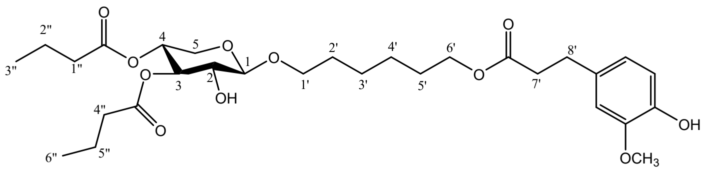

Purified by silica gel chromatography (EtOAc/Petroleum ether 30:70 to 50:50).

Liquid, 242 mg (52% yield for 11g/12g), ^{1}H NMR ($CDCl_3$, 500 MHz) : δ = 6.84 (d, J = 7.9 Hz, 1H, ArH), 4.67-6.75 (m, 2H, ArH), 5,14 (t, J = 9.0 Hz, 1H, H_3), 4.97 (td, J = 9.0 Hz, J = 5.3 Hz, 1H, H_4), 4.34 (d, J = 7.2 Hz, 1H, H_1), 4.05-4.16 (m, 3H, $H_{6'}$ and H_5), 3.84-3.91 (m, 4H, OCH_3 and $H_{1'}$), , 3.51-3.57 (m, 1H, H_2), 3.33 (dd, J = 11.6 Hz, J = 9.6 Hz, 1H, H_5), 2.90 (t, J = 7.7 Hz, 2H, $H_{8'}$), 2.61 (t, J = 7.7 Hz, 2H, $H_{7'}$), 2.25-2.40 (m, 4H, $H_{1''}$ and $H_{4''}$), 1.56-1.73 (m, 8H, $H_{2'}$, $H_{5'}$, $H_{2''}$ and $H_{5''}$), 1.30-1.41 (m, 4H, $H_{3'}$ and $H_{4'}$), 0.92-1.00 (m, 6H, $H_{3''}$ and $H_{6''}$); ^{13}C NMR ($CDCl_3$, 125 MHz) : δ = 173.3 (C═O), 173.2 (C=O), 172.6 (C=O), 146.5 (Ar), 144.0 (Ar), 132.5 (Ar), 120.9 (Ar), 114.4 (Ar), 111.0 (Ar), 103.1 (C_1), 73.2 (C_3), 72.0 (C_2), 69.9 ($C_{1'}$), 68.8 (C_4), 64.4 ($C_{6'}$), 62.6 (C_5), 55.9 (OCH_3), 36.3 ($C_{7'}$), 36.2 and 36.0 ($C_{1''}$ and $C_{4''}$), 30.8 ($C_{8'}$), 25.6 ($C_{3'}$ and $C_{4'}$), 18.4 and 18.3 ($C_{2''}$ and $C_{5''}$), 13.6 ($C_{3''}$ and $C_{6''}$); 2D experiment (HMBC): correlations between C═O 173.3 and H_3, C=O 173.2 and $H_{6'}$, C=O 172.6 and H_4; HRMS m/z calcd for $C_{29}H_{44}O_{11}Na$ $[M+Na]^+$ 591.2781, found 591,2780.

^{1}H and ^{13}C NMR spectra of compounds 1/2, 3a-3h, 3’g, 4a/4b, 6a/6b, 5e/6e, 5-6d, 7, 8f/8g, 9f/9g, 10f/10g, 11-12f and 11-12g.
HMBC NMR spectra of compounds 1/2, 3a-3h, 3’g, 4a/4b, 6a/6b, 5e/6e, 5-6d, 7, 8f/8g, 9f/9g, 10f/10g, 11-12f and 11-12g.

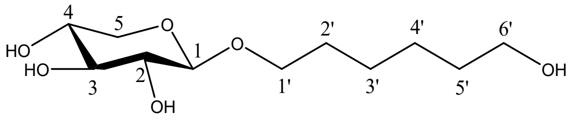

1

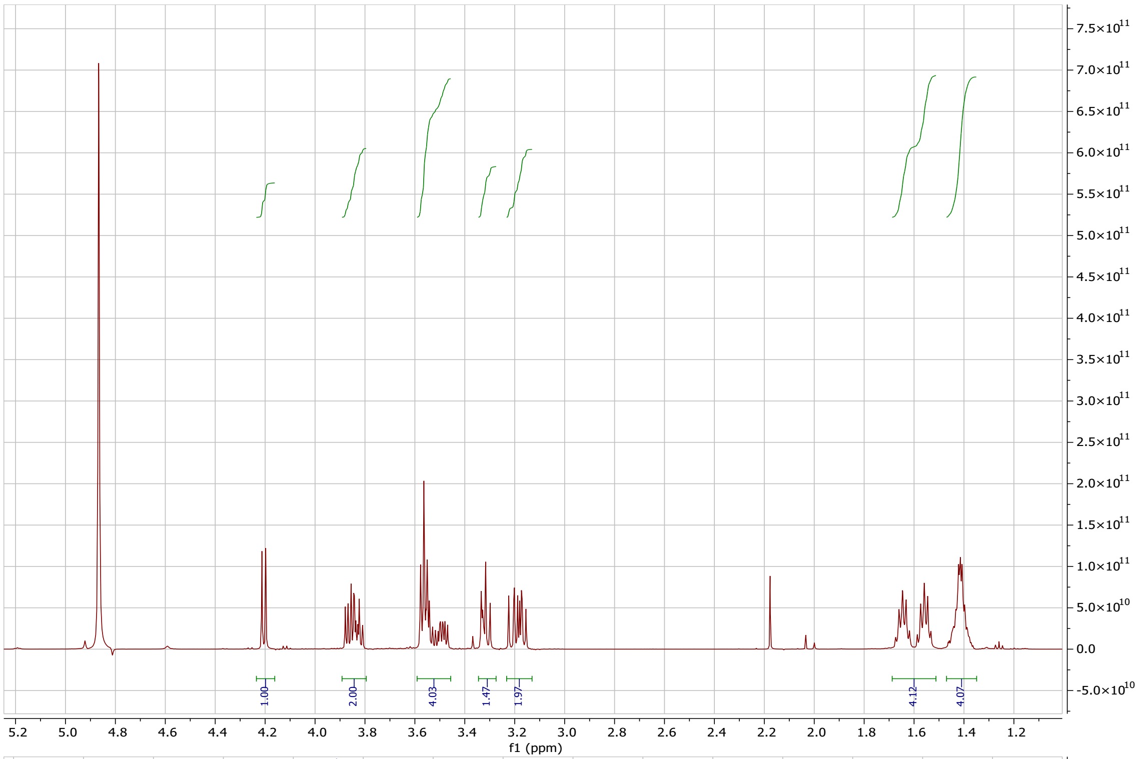

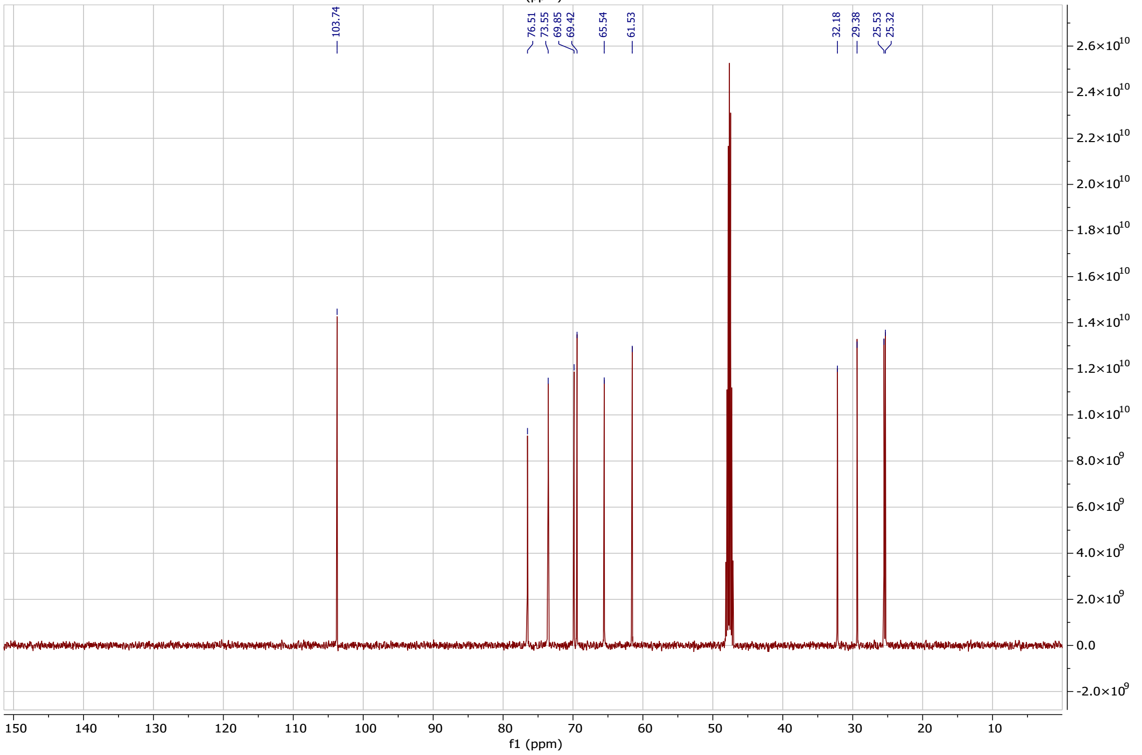

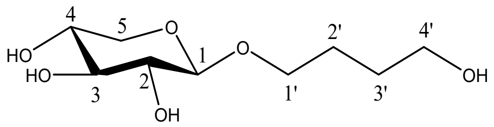
HO
HO
OH
O
O
OH
1
2
3
4
5
1'
2'
3'
4'

2

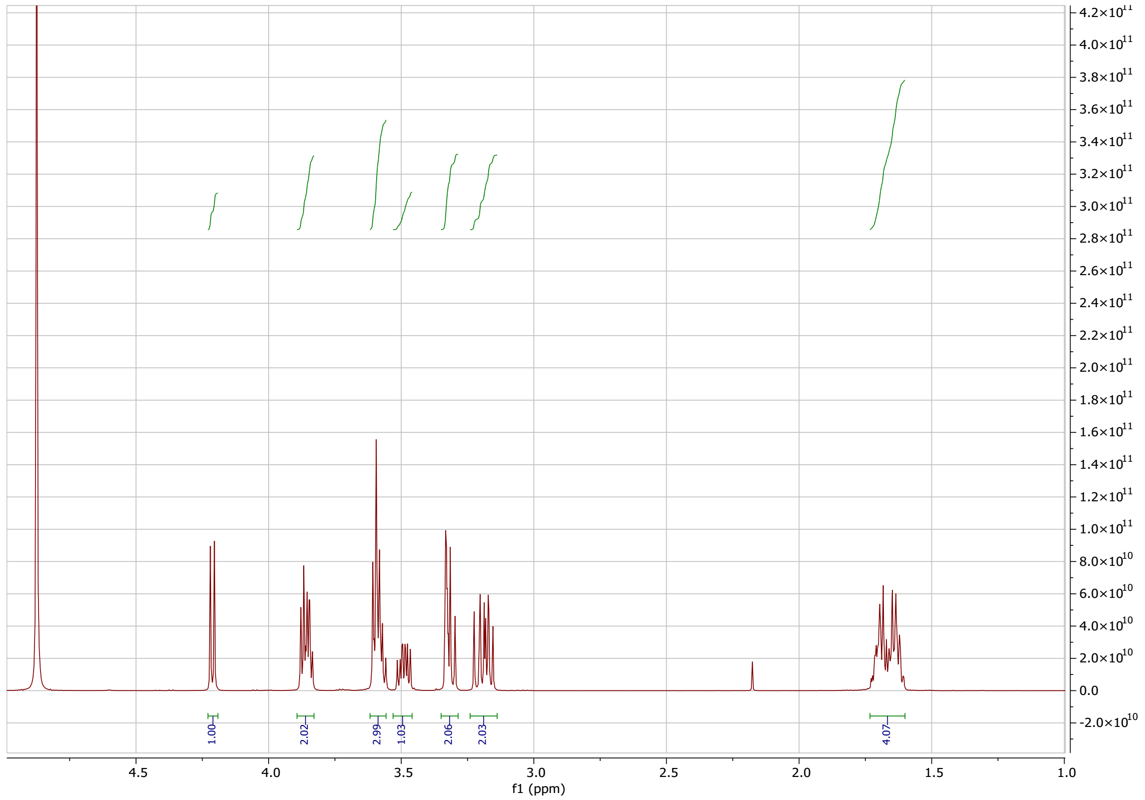
1.00
2.02
2.99
1.03
2.06
2.03
4.07
f1 (ppm)

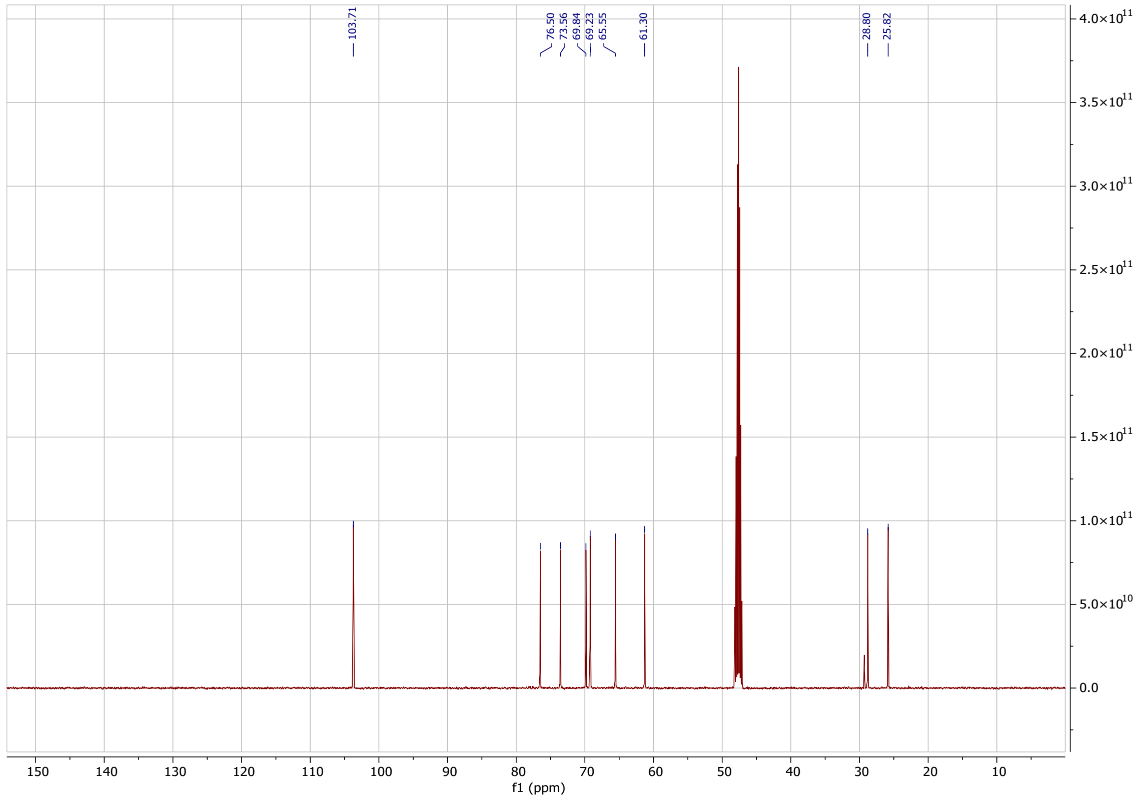
103.71
76.50
73.56
69.84
69.23
65.55
61.30
28.80
25.82
f1 (ppm)

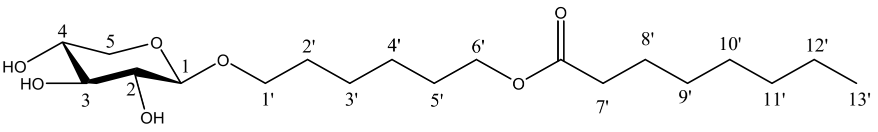
HO
HO
OH
O
1
2
3
4
5
1'
2'
3'
4'
5'
6'
7'
8'
9'
10'
11'
12'
13'

3a

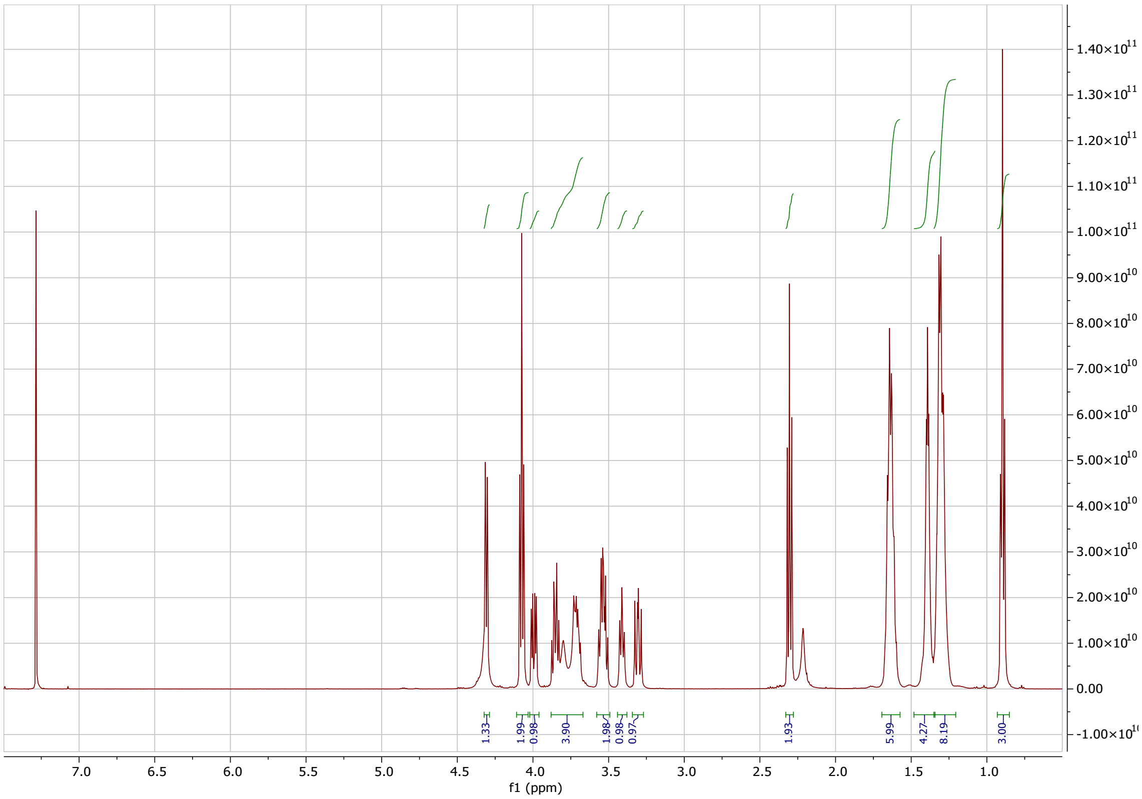
1.33
1.99
0.98
3.90
1.98
0.98
0.97
1.93
5.99
4.27
8.19
3.00
f1 (ppm)

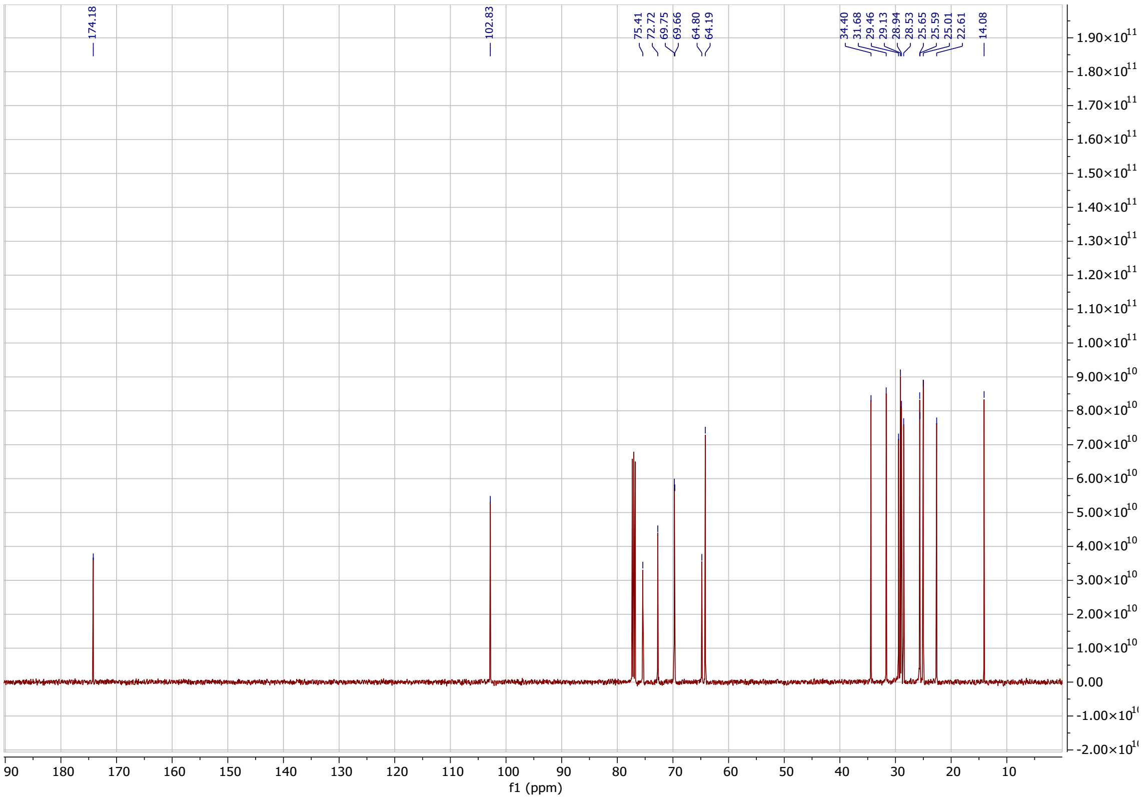
174.18
102.83
75.41
72.72
69.75
69.66
64.80
64.19
34.40
31.68
29.46
29.13
28.94
28.53
25.65
25.59
25.01
22.61
14.08
f1 (ppm)

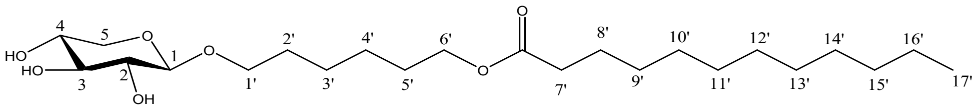

3b

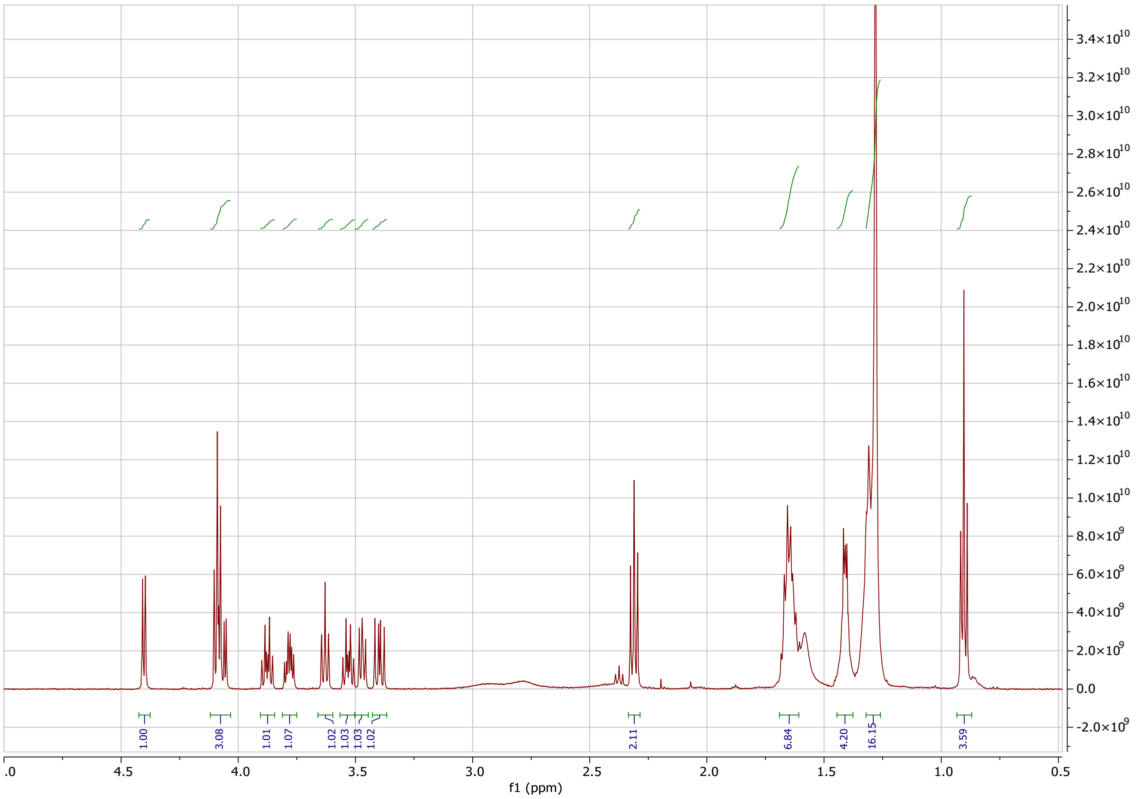

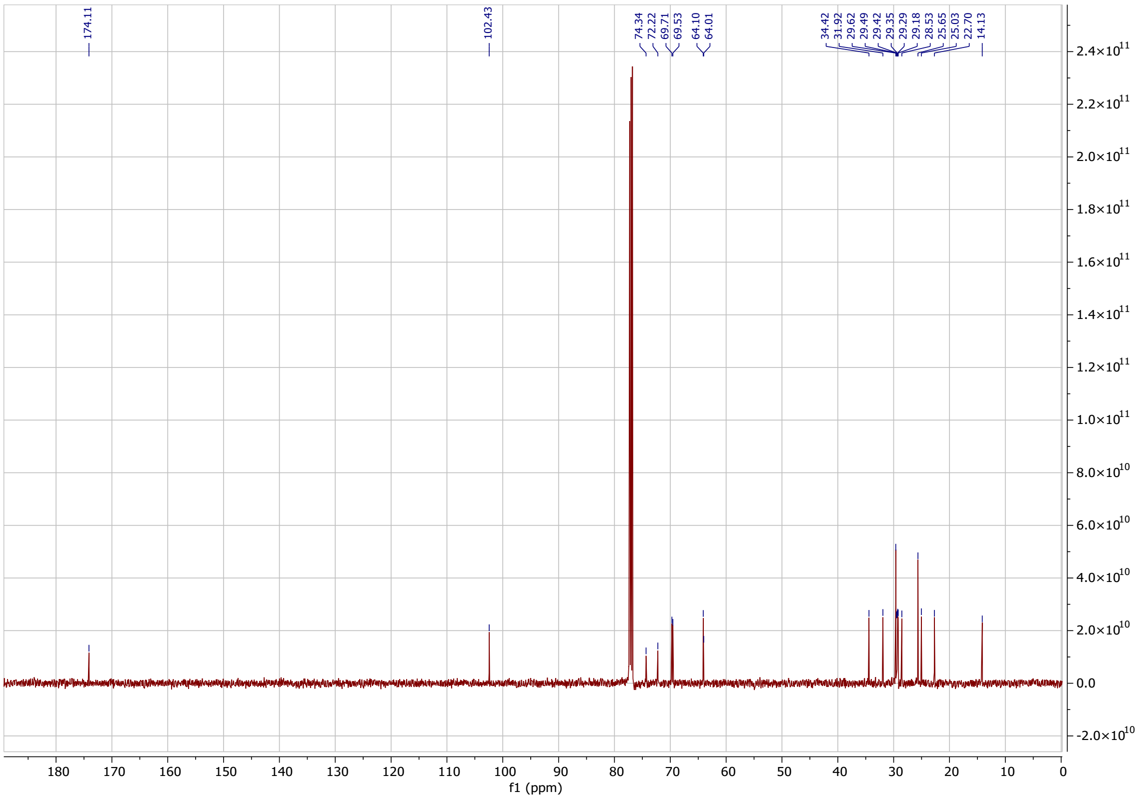

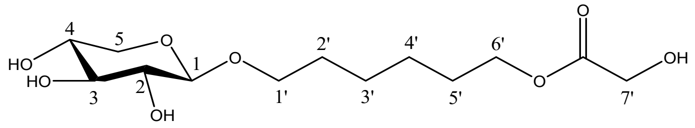
HO
HO
OH
O
OH
O
O
1
2
3
4
5
1'
2'
3'
4'
5'
6'
7'

3c

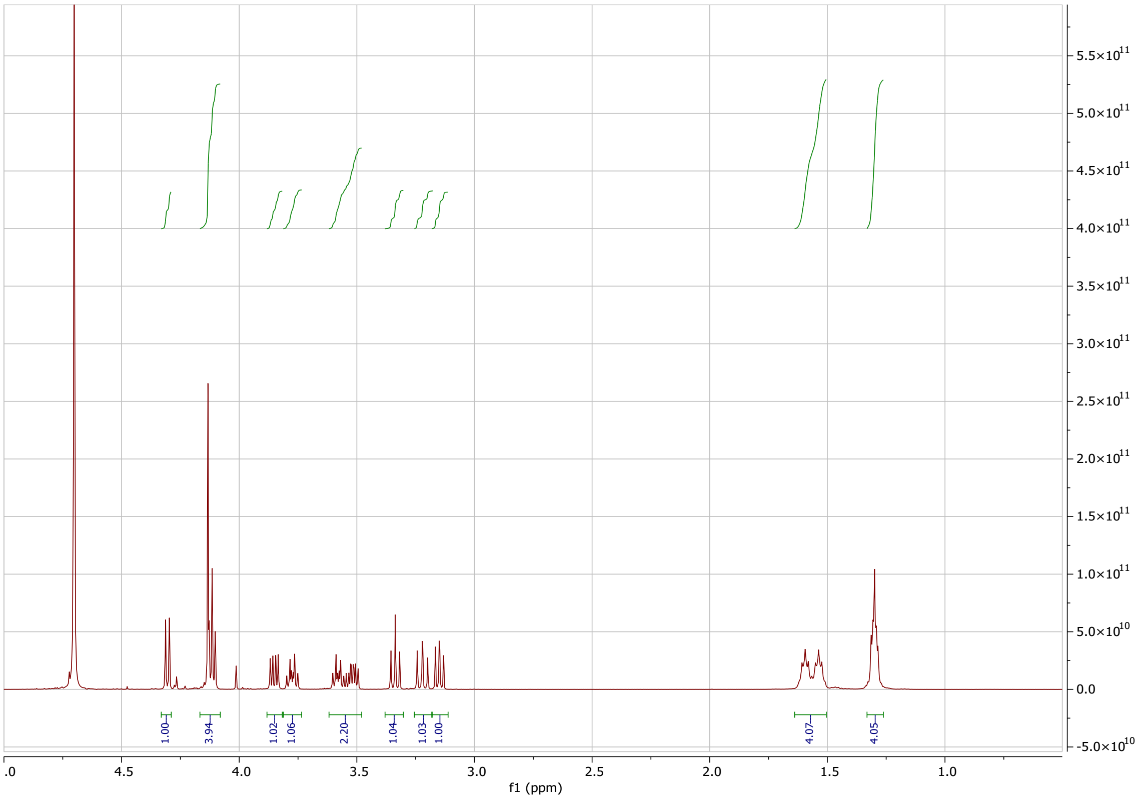
1.00
3.94
1.02
1.06
2.20
1.04
1.03
1.00
4.07
4.05
f1 (ppm)

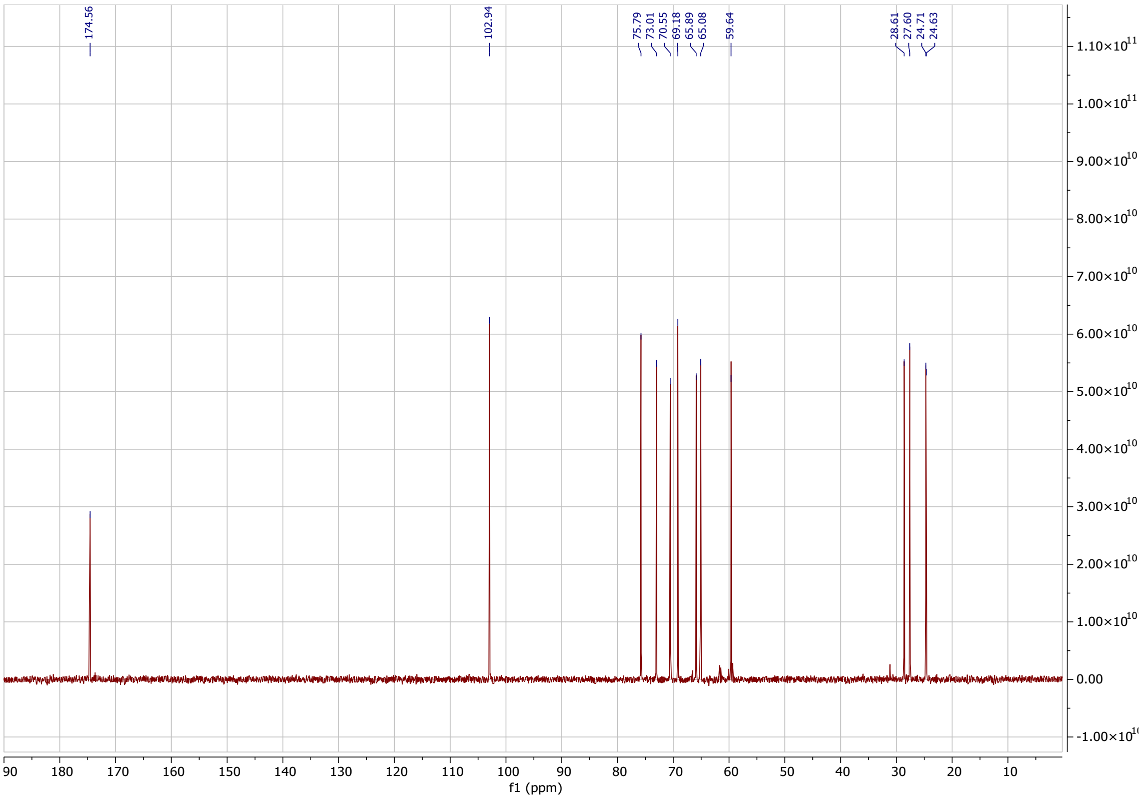
174.56
102.94
75.79
73.01
70.55
69.18
65.89
65.08
59.64
28.61
27.60
24.71
24.63
f1 (ppm)

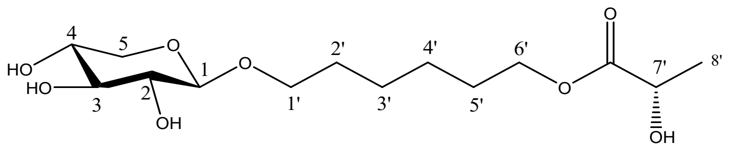
HO
HO
OH
O
OH
4
5
1
2
3
1'
2'
3'
4'
5'
6'
7'
8'

3d

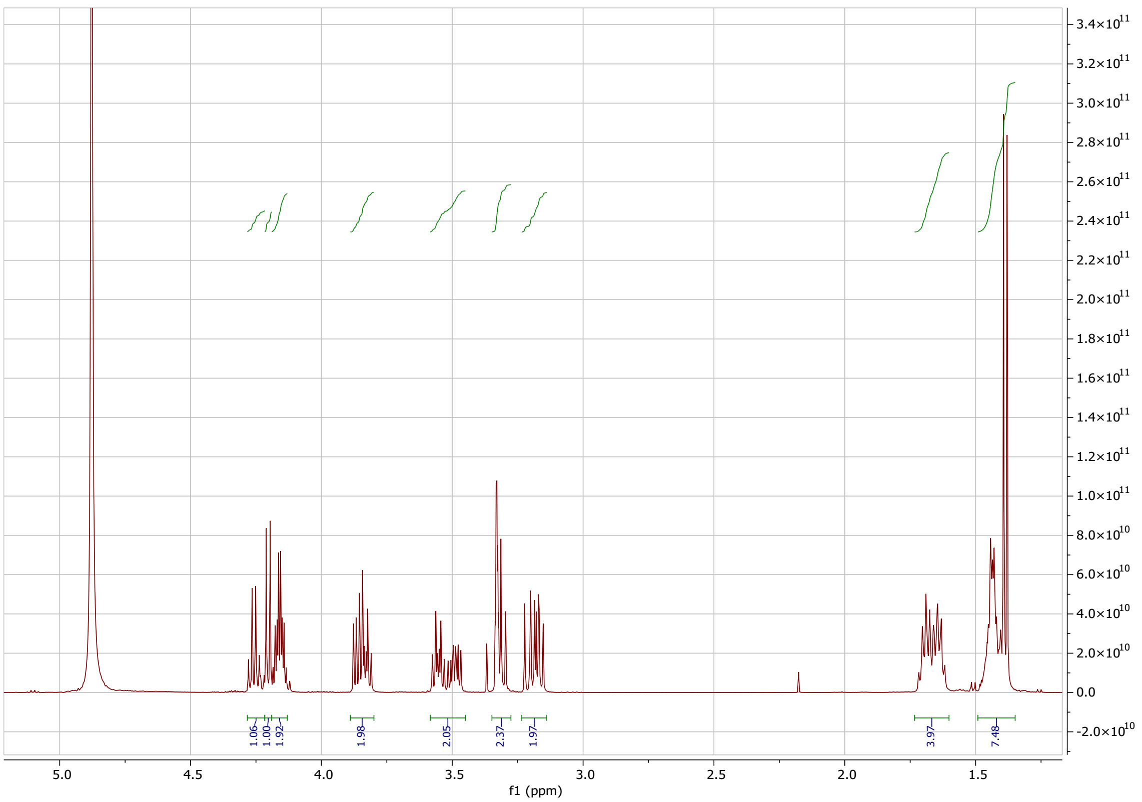
1.06
1.00
1.92
1.98
2.05
2.37
1.97
3.97
7.48
f1 (ppm)

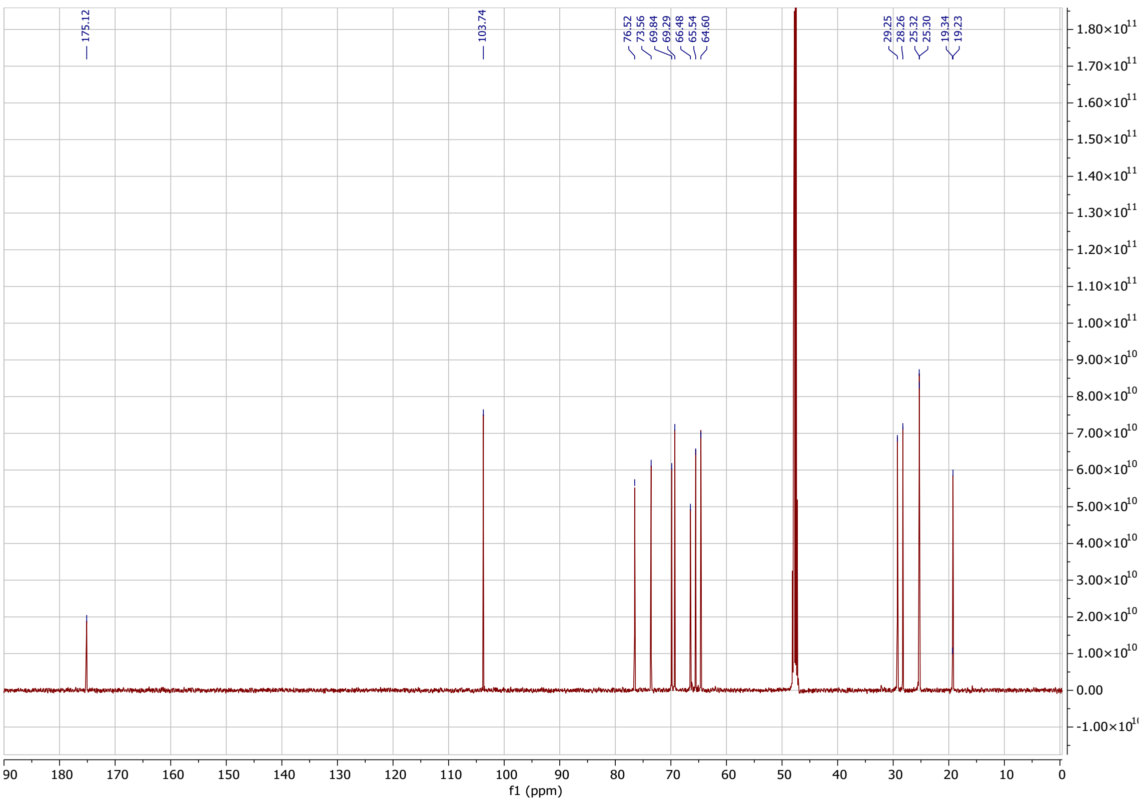
175.12
103.74
76.52
73.56
69.84
69.29
66.48
65.54
64.60
29.25
28.26
25.32
25.30
19.34
19.23
f1 (ppm)

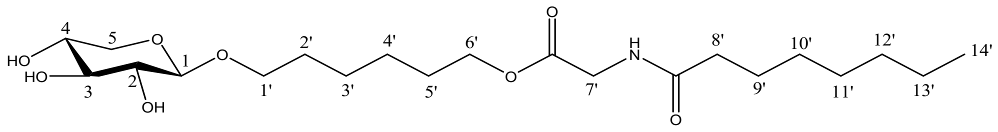
HO
HO
OH
O
1
2
3
4
5
O
1'
2'
3'
4'
5'
6'
O
O
7'
H
N
O
8'
9'
10'
11'
12'
13'
14'

3e

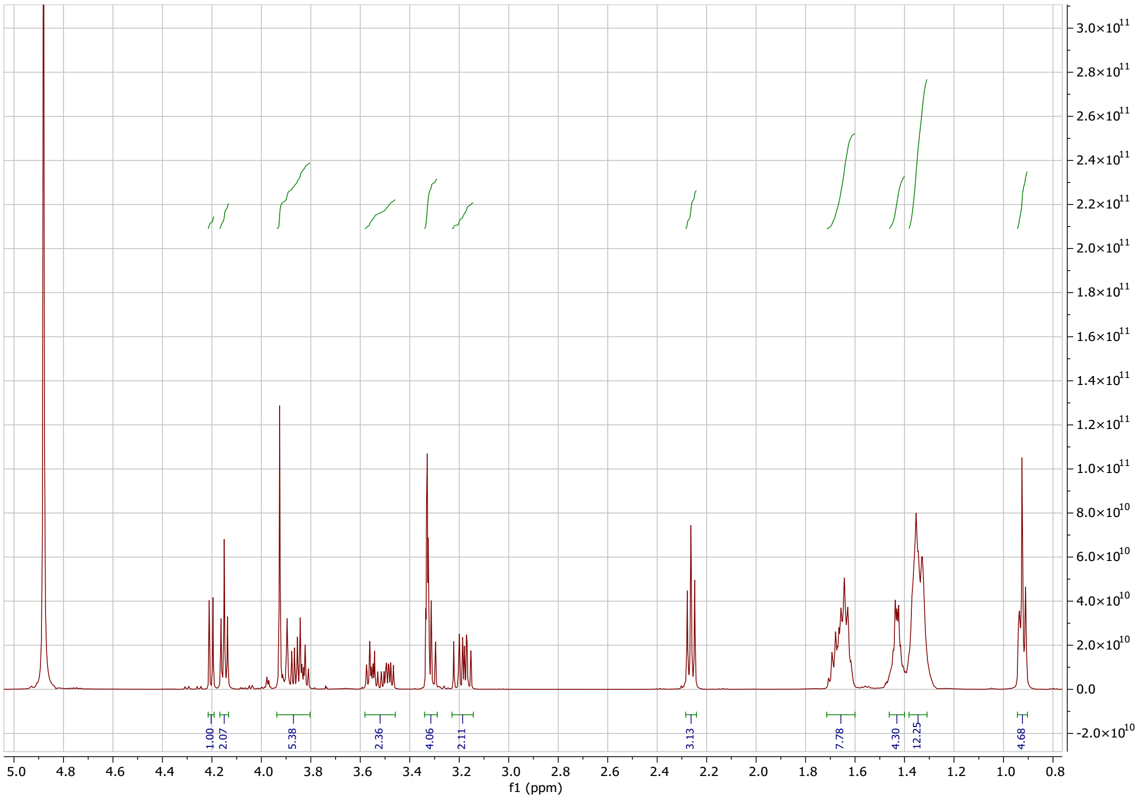
1.00
2.07
5.38
2.36
4.06
2.11
3.13
7.78
4.30
12.25
4.68
f1 (ppm)

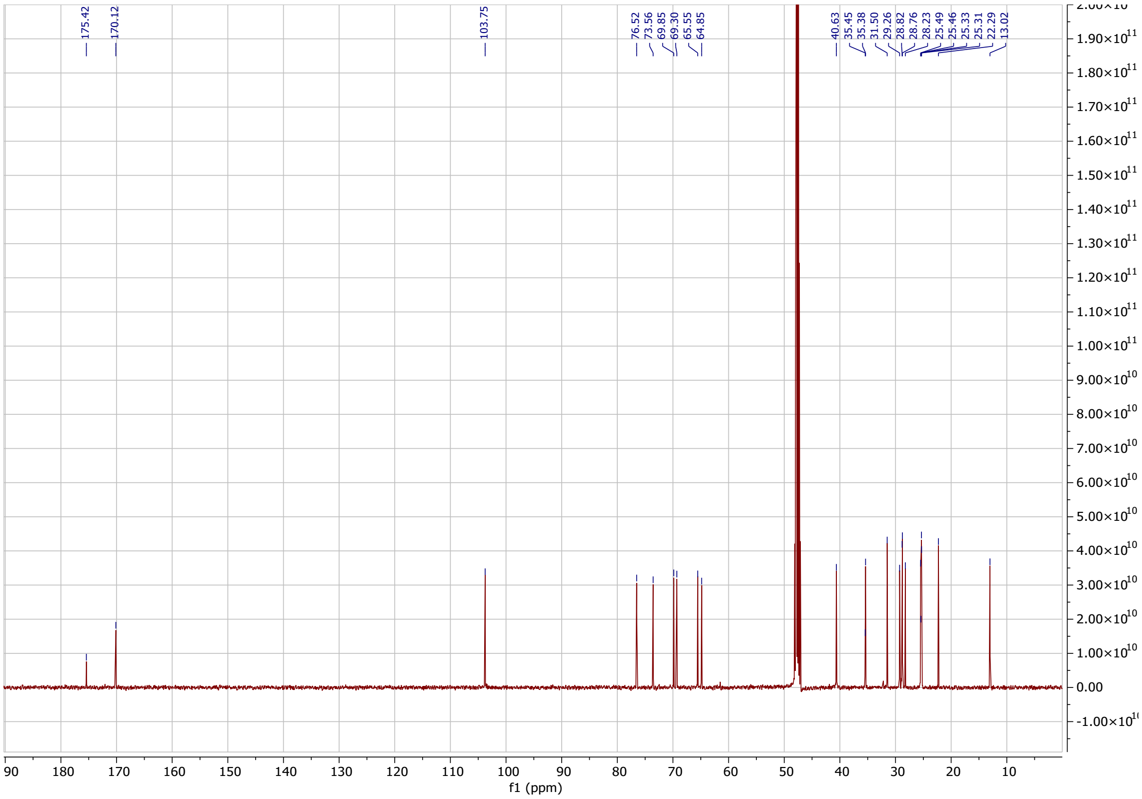
175.42
170.12
103.75
76.52
73.56
69.85
69.30
65.55
64.85
40.63
35.45
35.38
31.50
29.26
28.82
28.76
28.23
25.49
25.46
25.33
25.31
22.29
13.02
f1 (ppm)

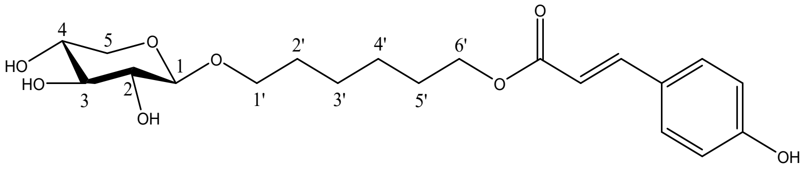

3f

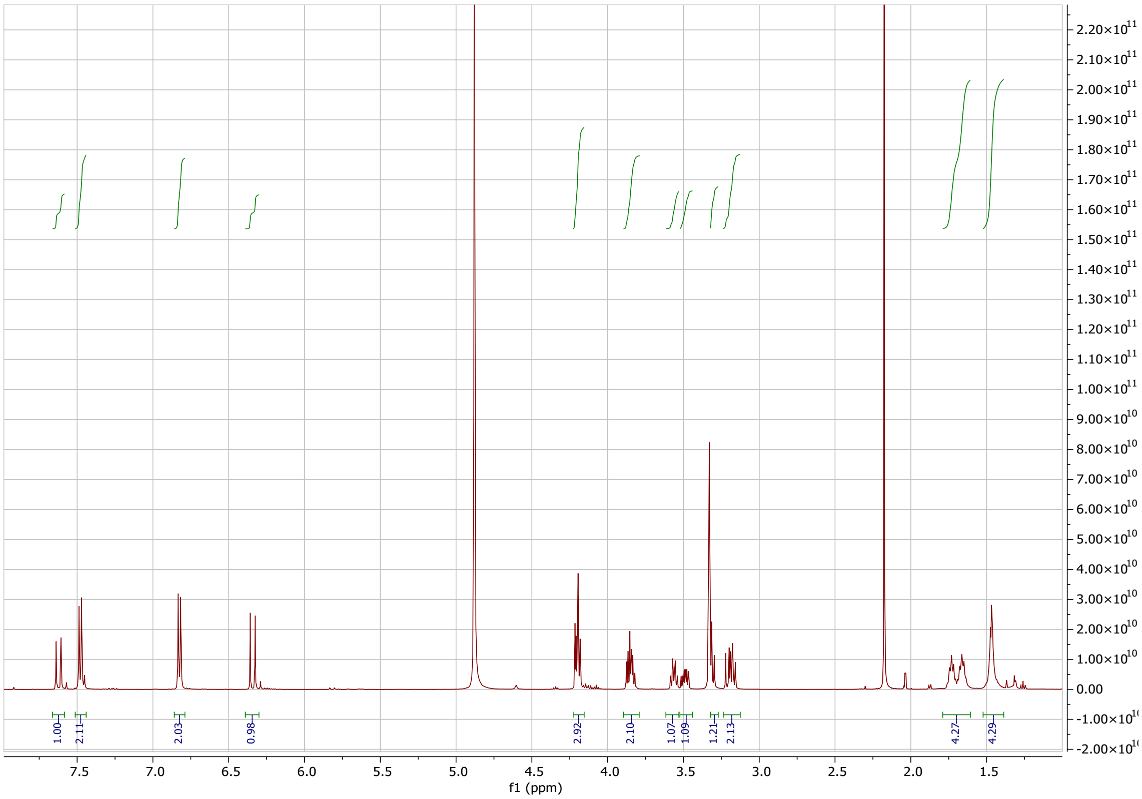

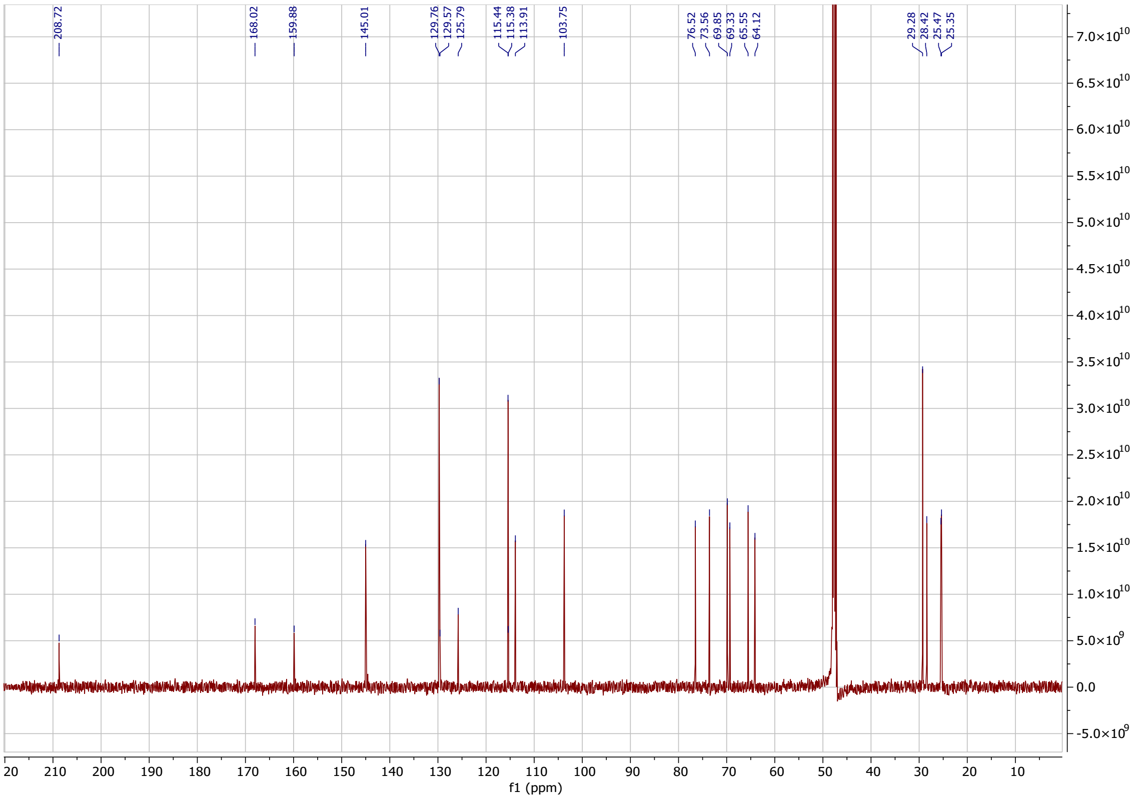

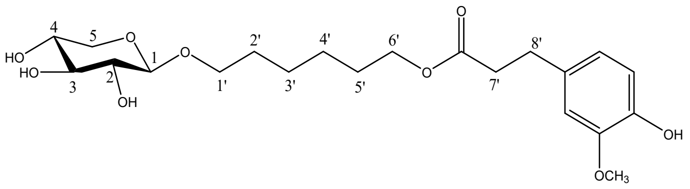
HO
HO
OH
OH
O
O
O
O
OCH3
1
2
3
4
5
1'
2'
3'
4'
5'
6'
7'
8'

3g

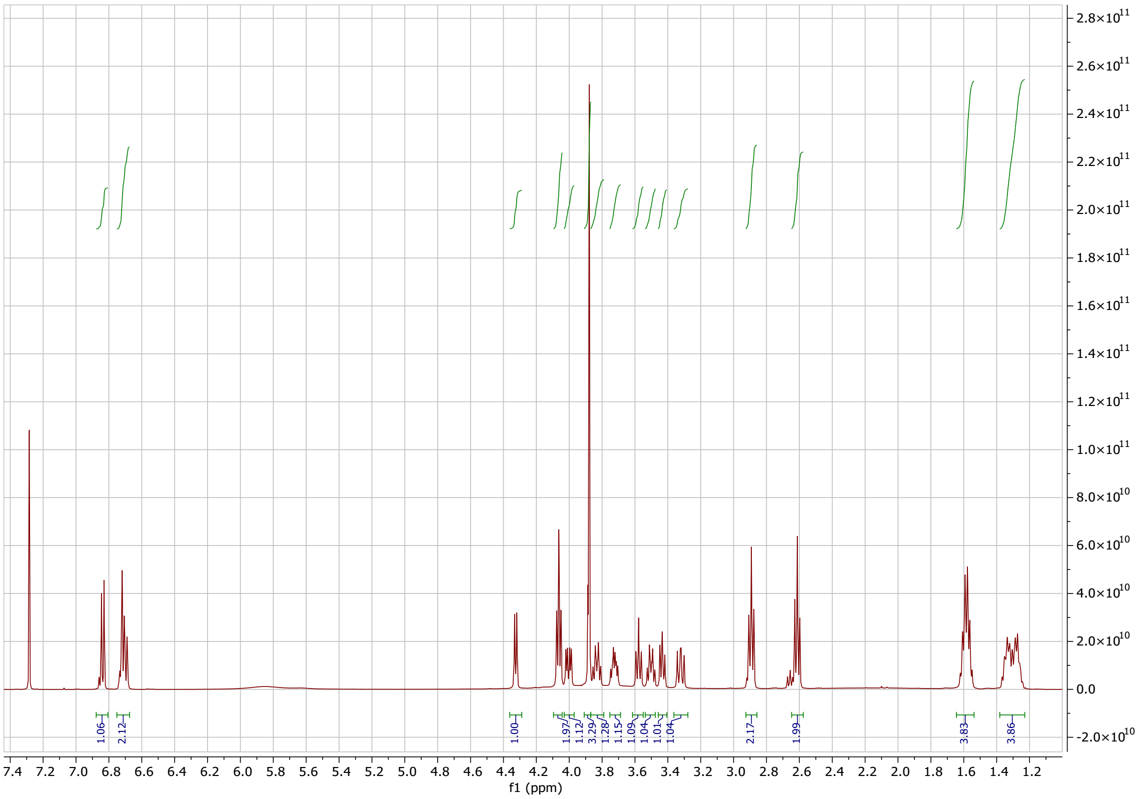
1.06
2.12
1.00
1.97
1.12
3.29
1.28
1.15
1.09
1.04
1.01
1.04
2.17
1.99
3.83
3.86
f1 (ppm)

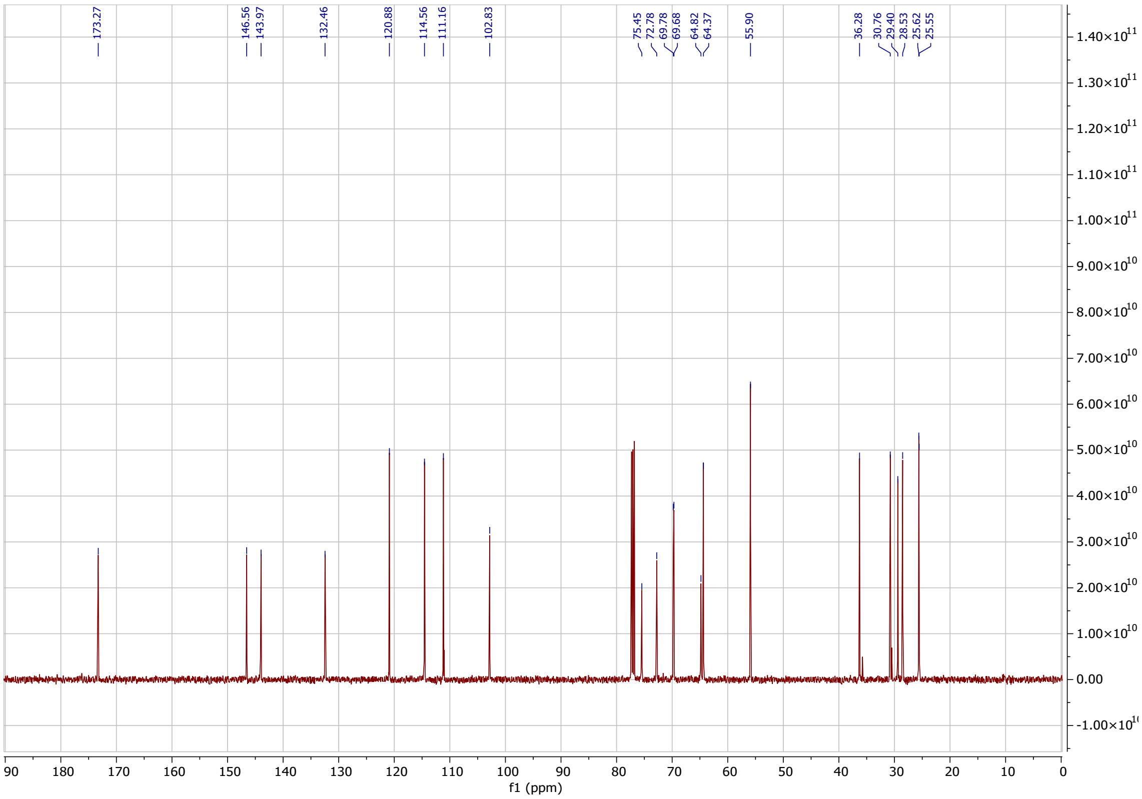
173.27
146.56
143.97
132.46
120.88
114.56
111.16
102.83
75.45
72.78
69.78
69.68
64.82
64.37
55.90
36.28
30.76
29.40
28.53
25.62
25.55
f1 (ppm)

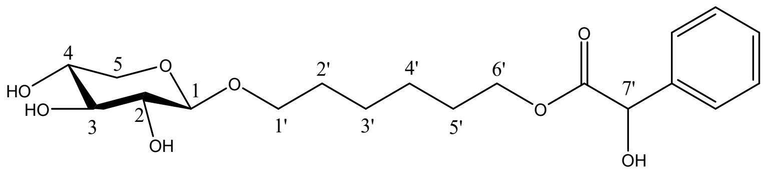
HO
HO
OH
O
O
O
O
OH
1
2
3
4
5
1'
2'
3'
4'
5'
6'
7'

3h

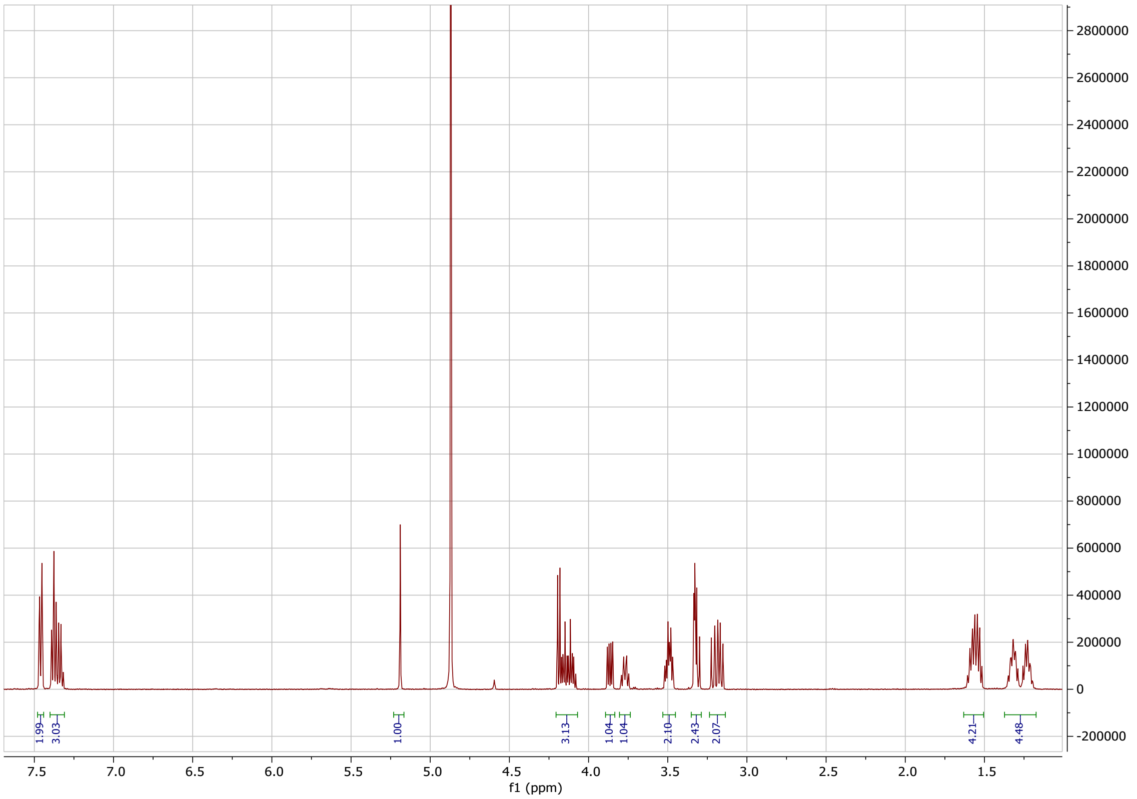
1.99
3.03
1.00
3.13
1.04
1.04
2.10
2.43
2.07
4.21
4.48
f1 (ppm)

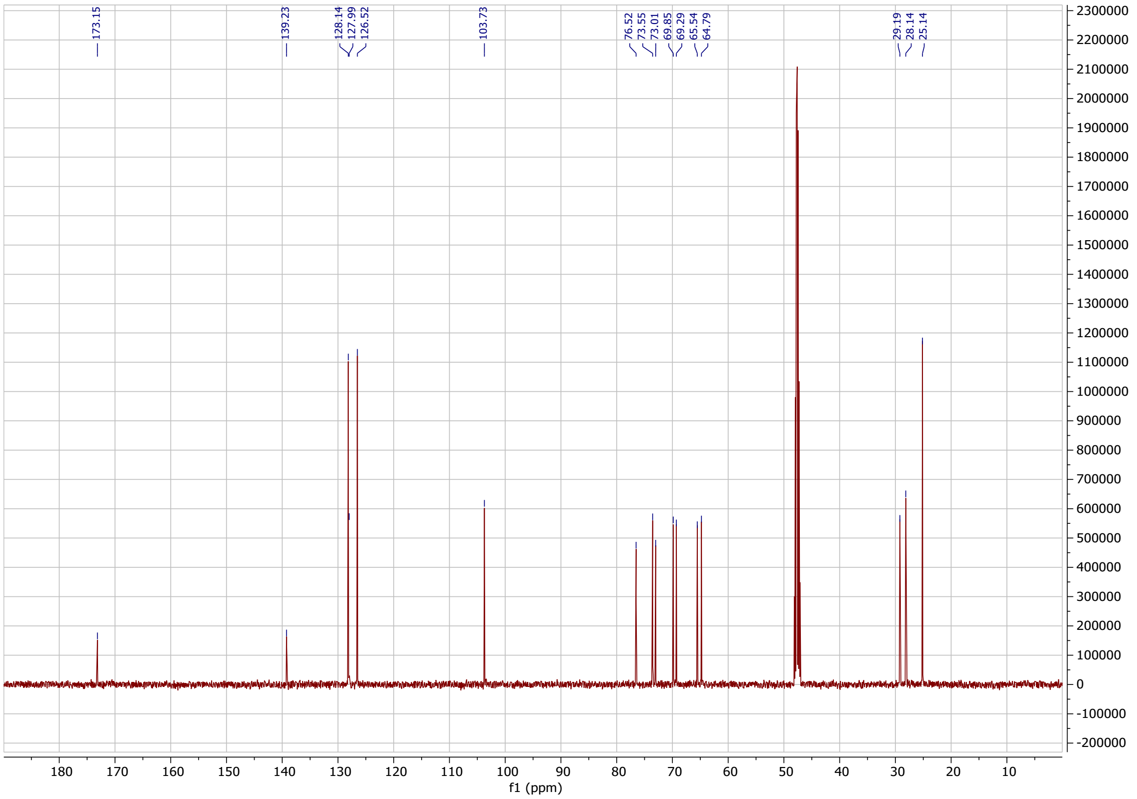
173.15
139.23
128.14
127.99
126.52
103.73
76.52
73.55
73.01
69.85
69.29
65.54
64.79
29.19
28.14
25.14
f1 (ppm)

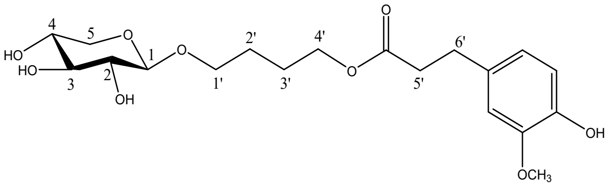
HO
HO
OH
O
1
2
3
4
5
1'
2'
3'
4'
5'
6'
OH
OCH3

3’g

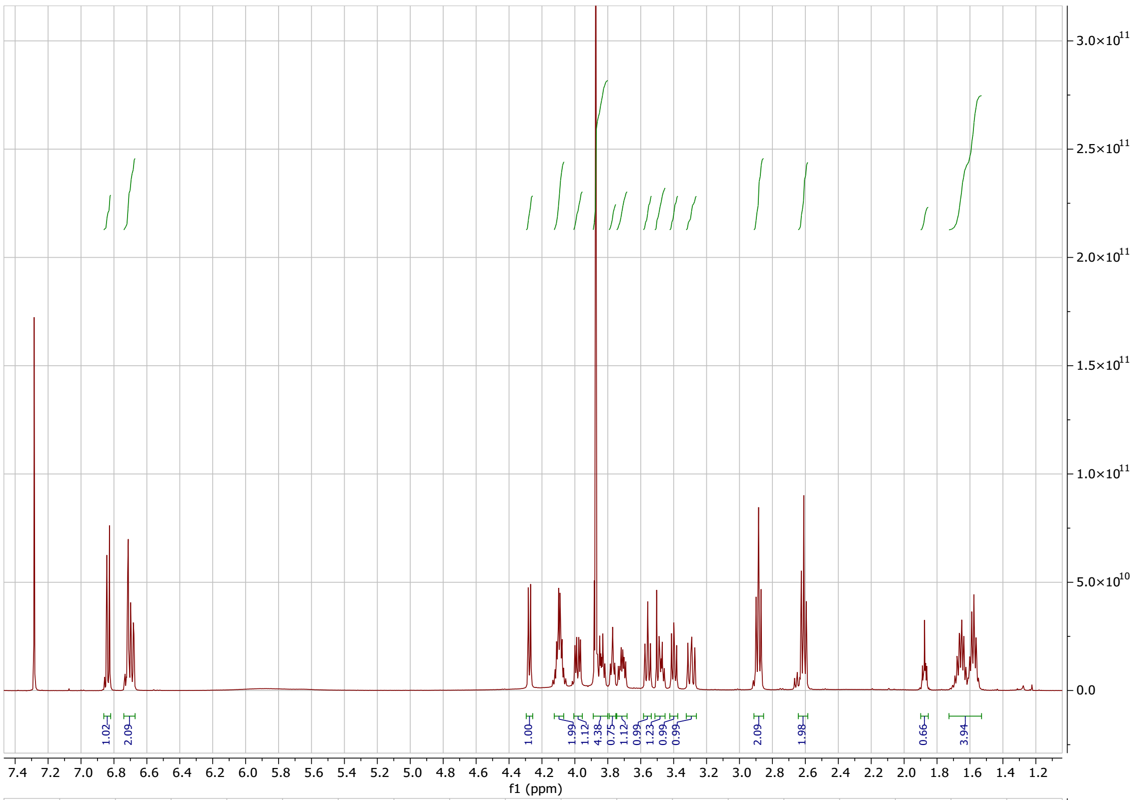
1.02
2.09
1.00
1.99
1.12
4.38
0.75
1.12
0.99
1.23
0.99
0.99
2.09
1.98
0.66
3.94
f1 (ppm)

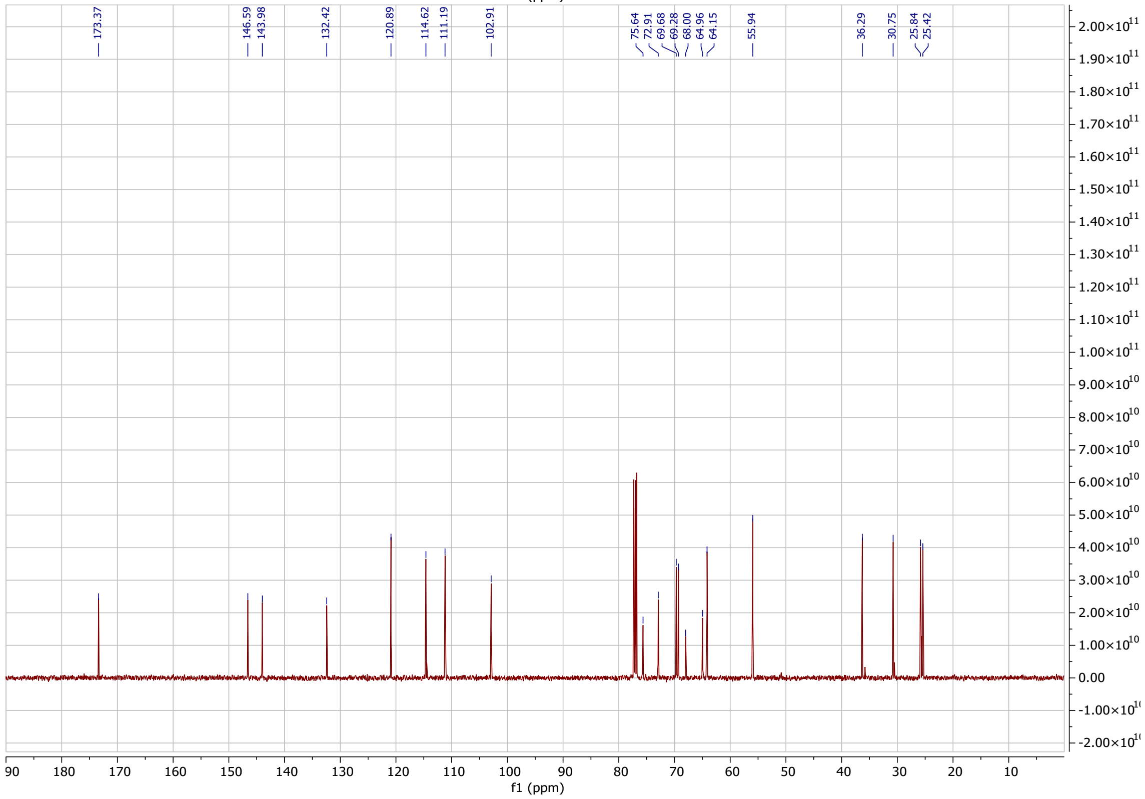
173.37
146.59
143.98
132.42
120.89
114.62
111.19
102.91
75.64
72.91
69.68
69.28
68.00
64.96
64.15
55.94
36.29
30.75
25.84
25.42
f1 (ppm)

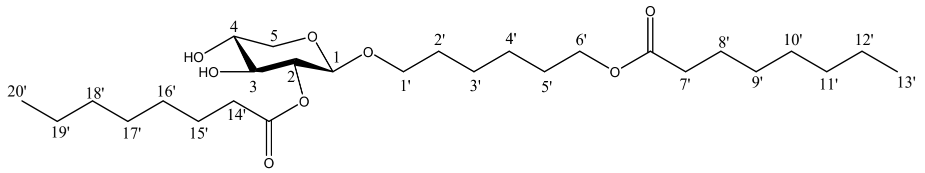

4a

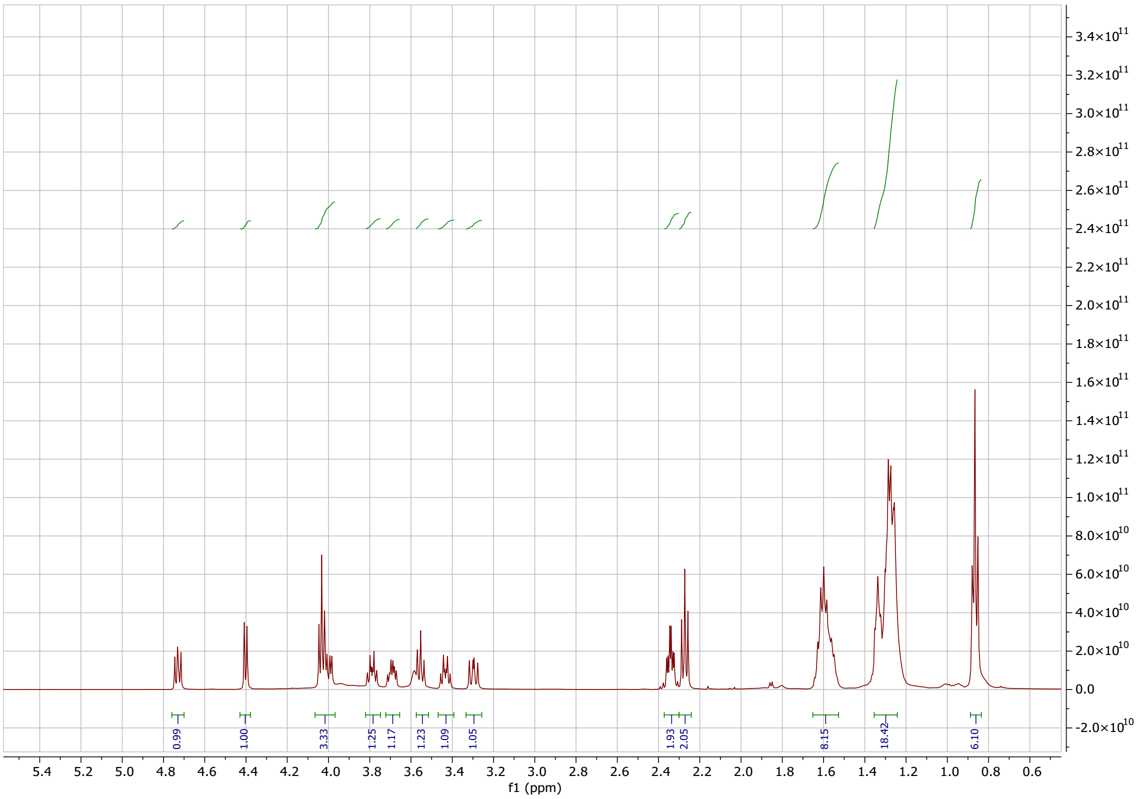
0.99
1.00
3.33
1.25
1.17
1.23
1.09
1.05
1.93
2.05
8.15
18.42
6.10
f1 (ppm)

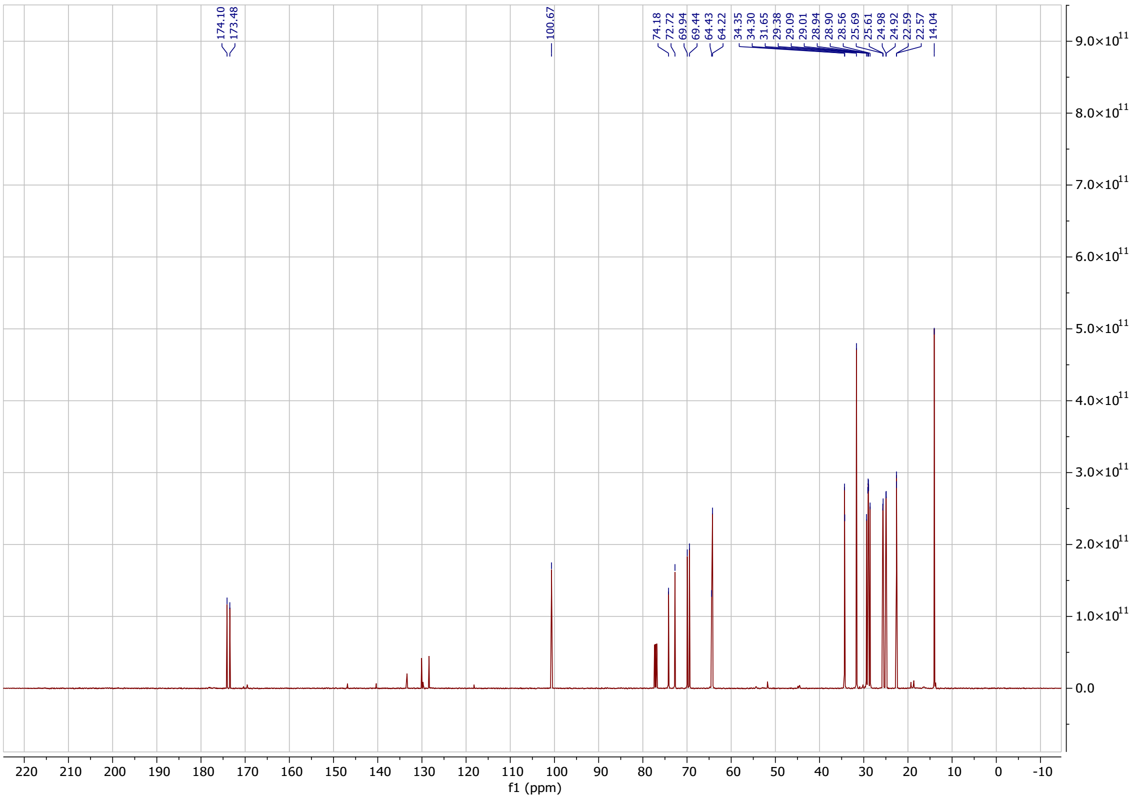
174.10
173.48
100.67
74.18
72.72
69.94
69.44
64.43
64.22
34.35
34.30
31.65
29.38
29.09
29.01
28.94
28.90
28.56
25.69
25.61
24.98
24.92
22.59
22.57
14.04
f1 (ppm)

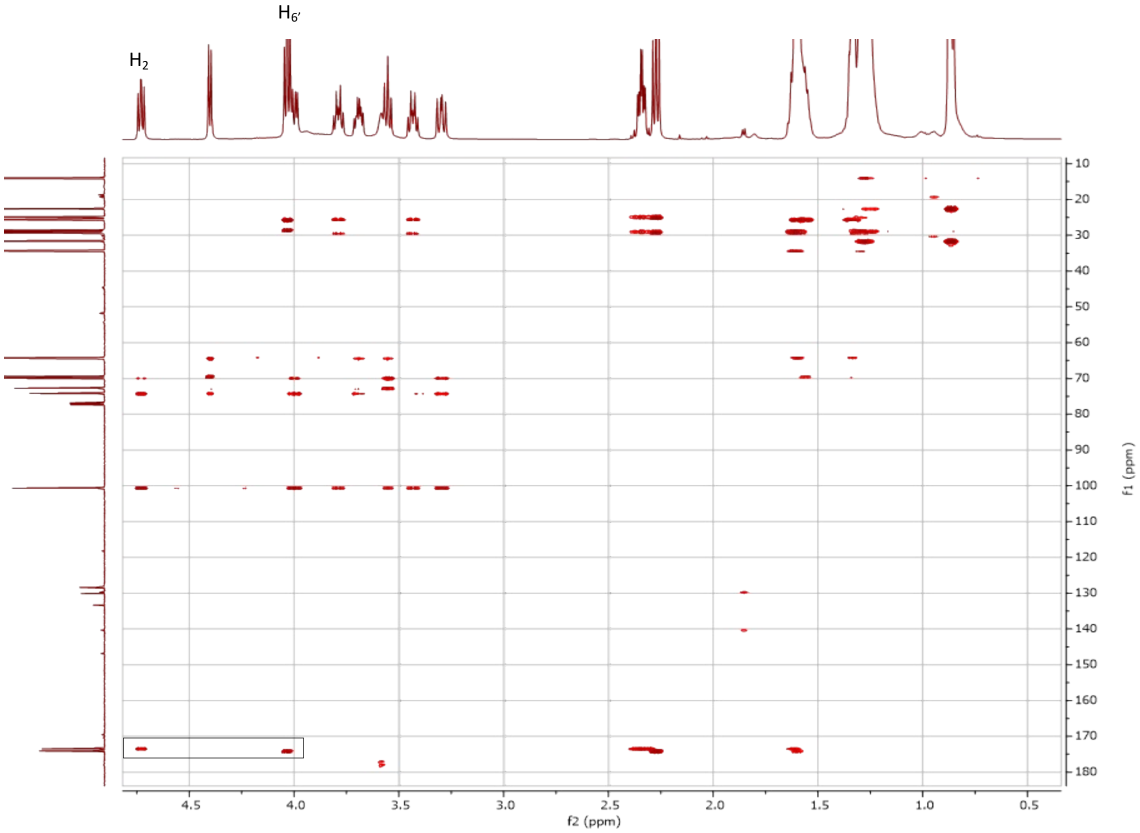

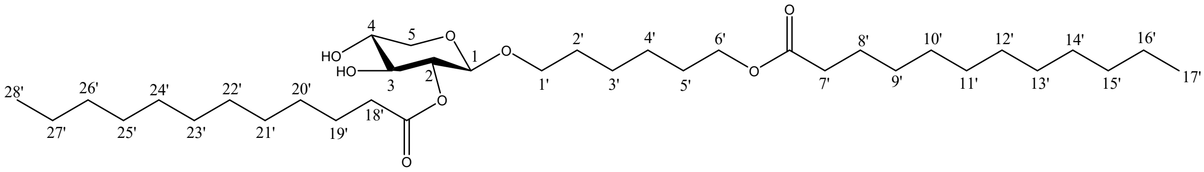

4b

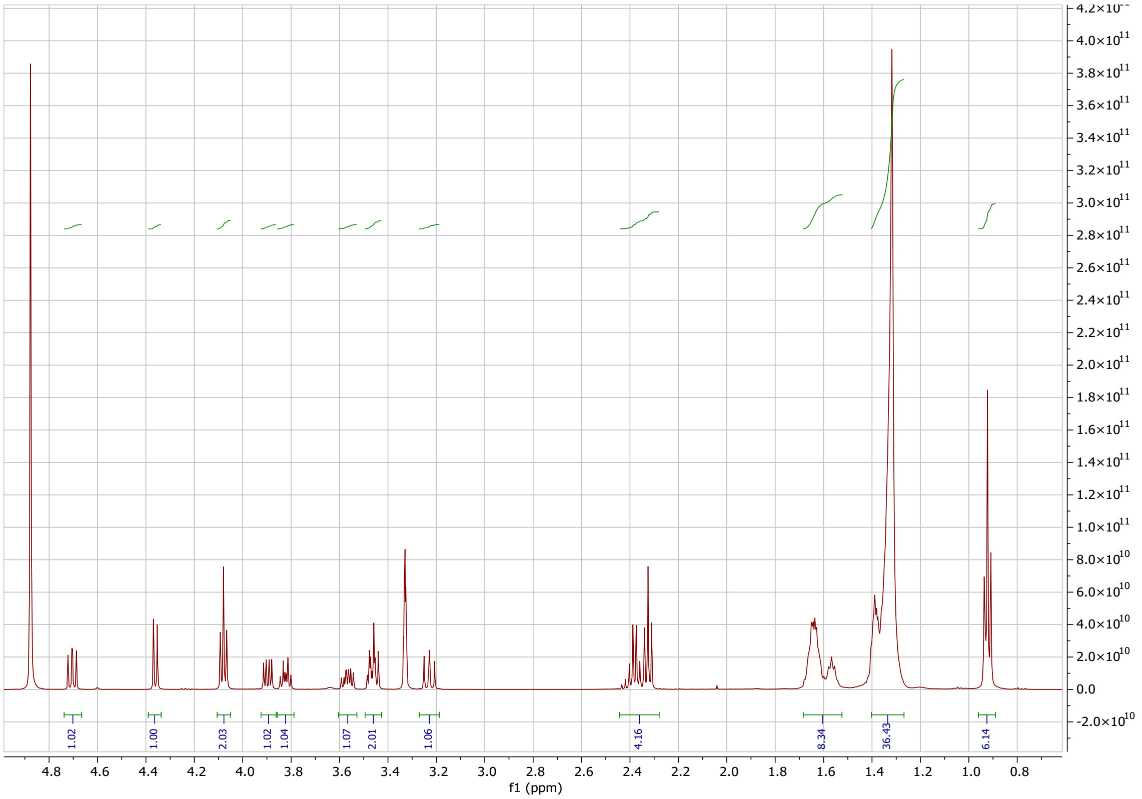

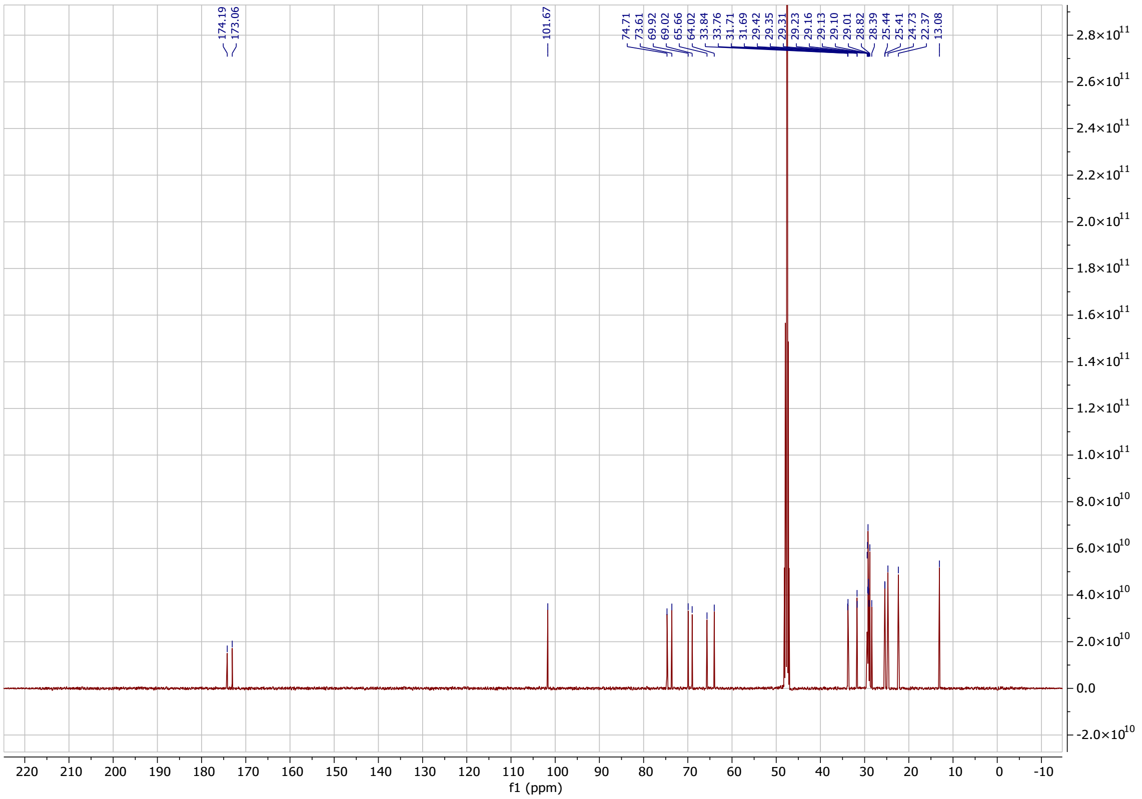
174.19
173.06
101.67
74.71
73.61
69.92
69.02
65.66
64.02
33.84
33.76
31.71
31.69
29.42
29.35
29.31
29.23
29.16
29.13
29.10
29.01
28.82
28.39
25.44
25.41
24.73
22.37
13.08
f1 (ppm)

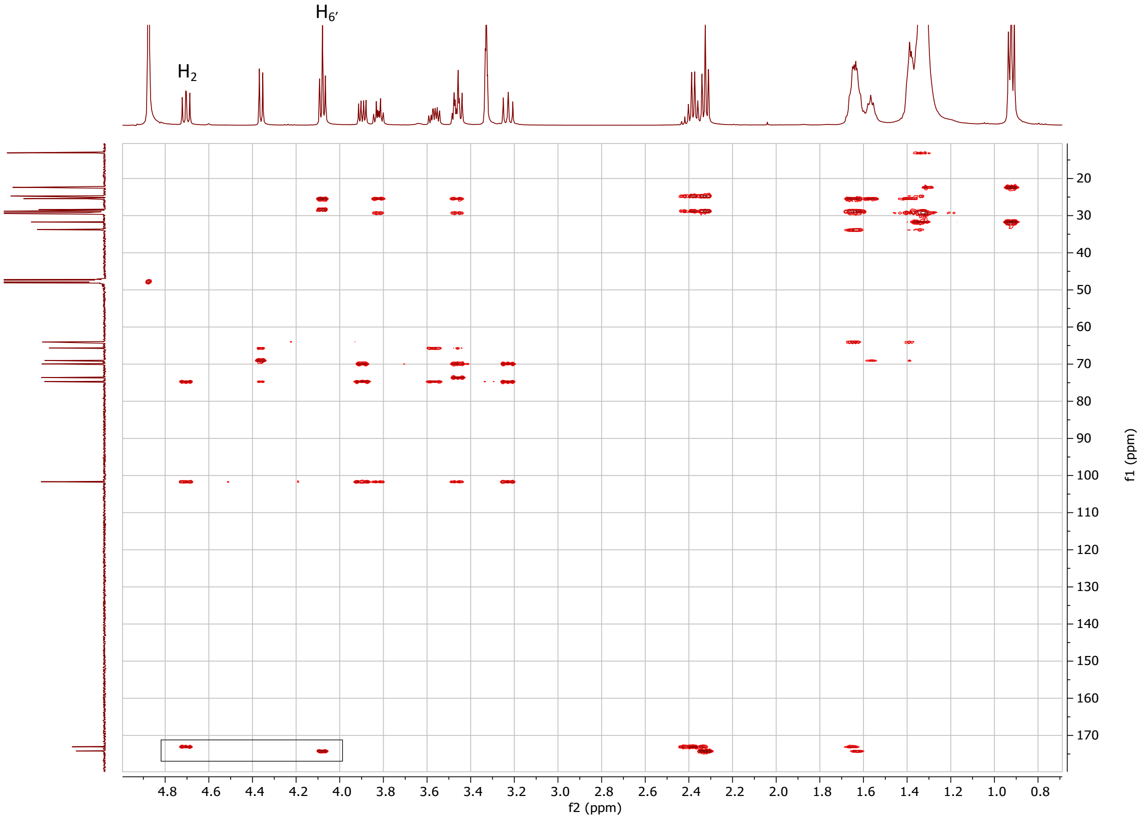
H2
H6′
f2 (ppm)
f1 (ppm)

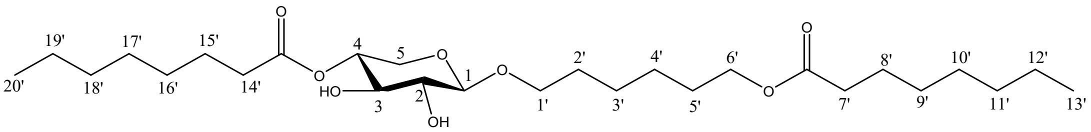
O
O
19'
17'
15'
4
5
O
1
O
2'
4'
6'
O
8'
10'
12'
20'
18'
16'
14'
O
HO
3
2
OH
1'
3'
5'
O
7'
9'
11'
13'

6a

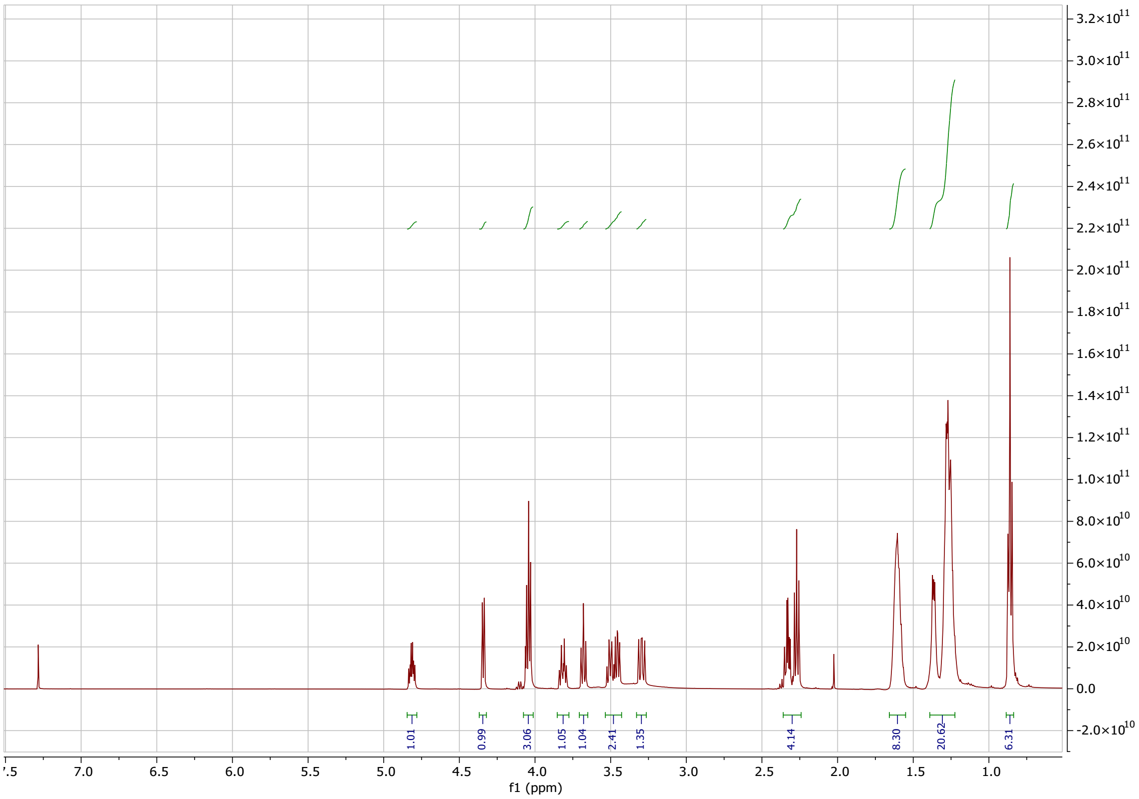
1.01
0.99
3.06
1.05
1.04
2.41
1.35
4.14
8.30
20.62
6.31
f1 (ppm)

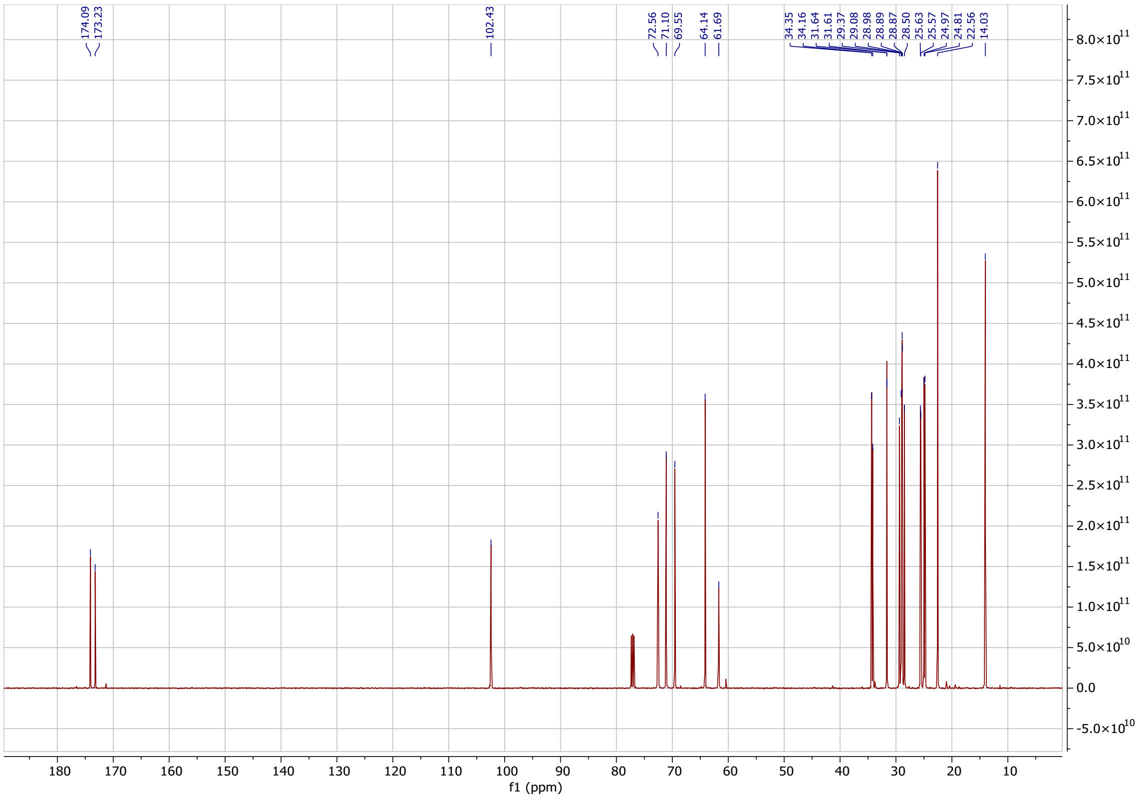
174.09
173.23
102.43
72.56
71.10
69.55
64.14
61.69
34.35
34.16
31.64
31.61
29.37
29.08
28.98
28.89
28.87
28.50
25.63
25.57
24.97
24.81
22.56
14.03
f1 (ppm)

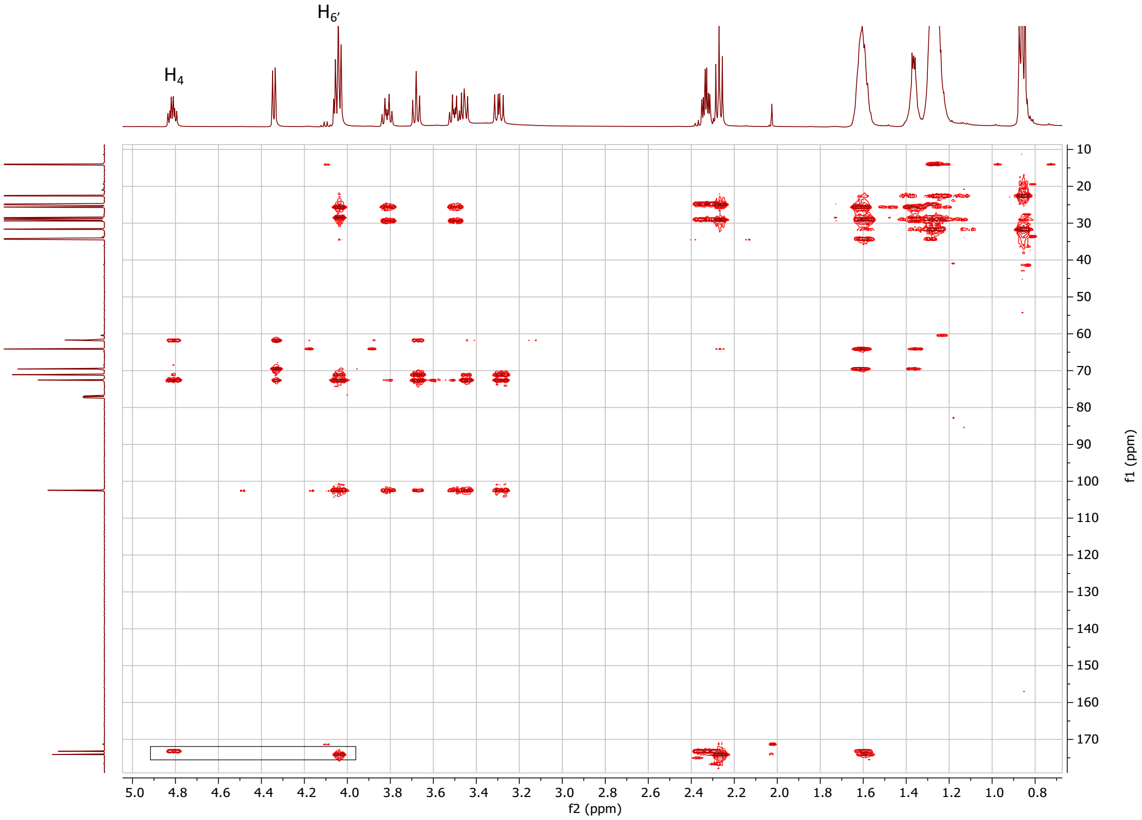
H4
H6′
f2 (ppm)
f1 (ppm)

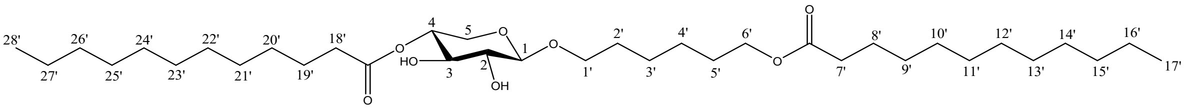
HO
OH
O

6b

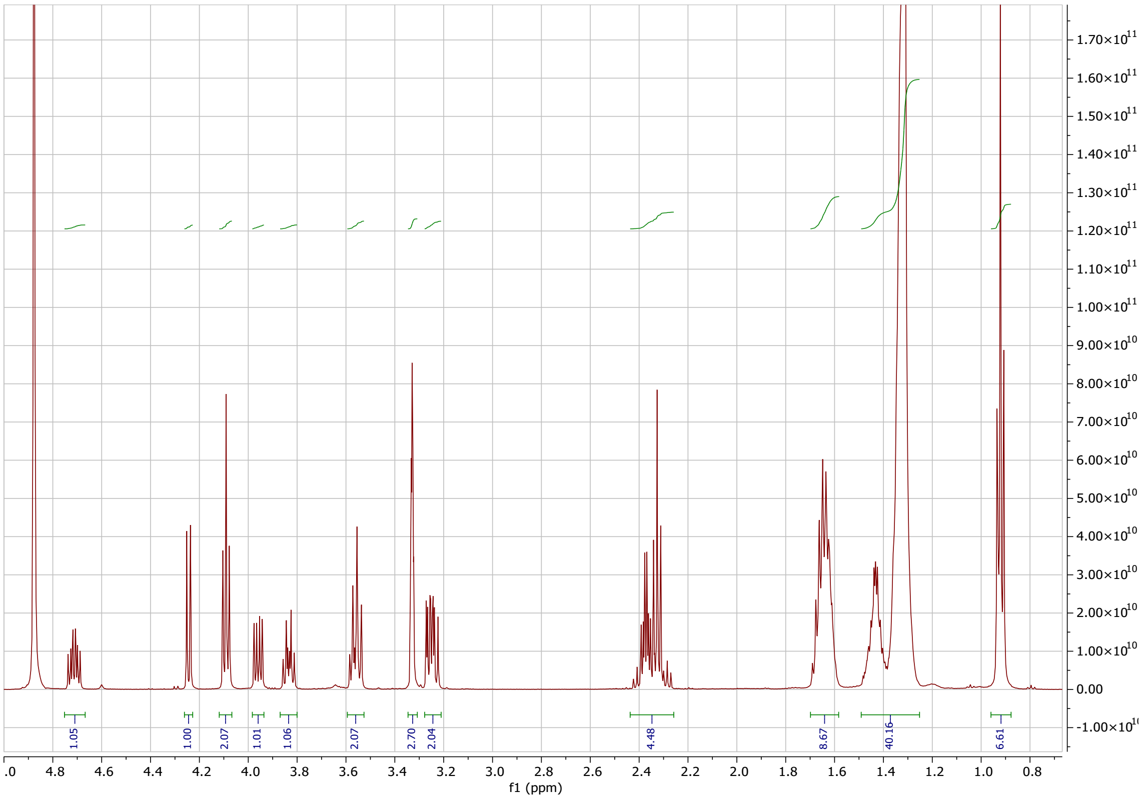
f1 (ppm)
1.05
1.00
2.07
1.01
1.06
2.07
2.70
2.04
4.48
8.67
40.16
6.61

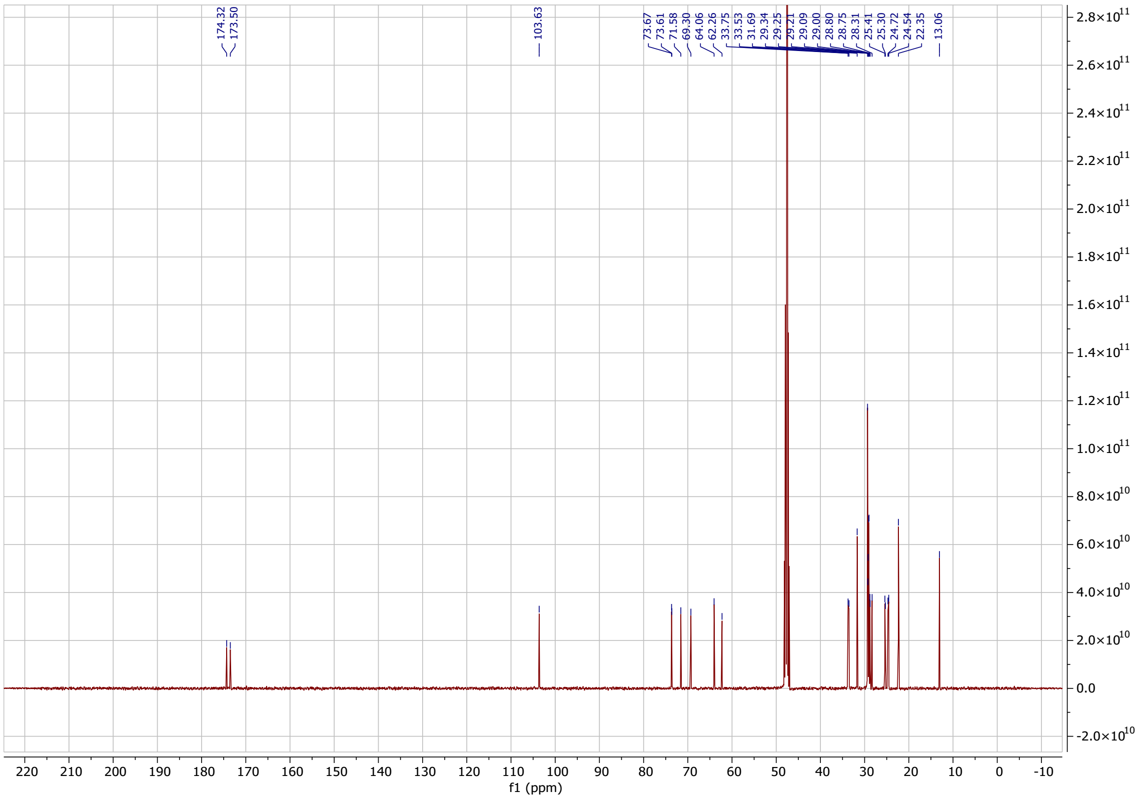
174.32
173.50
103.63
73.67
73.61
71.58
69.30
64.06
62.26
33.75
33.53
31.69
29.34
29.25
29.21
29.09
29.00
28.80
28.75
28.31
25.41
25.30
24.72
24.54
22.35
13.06
f1 (ppm)

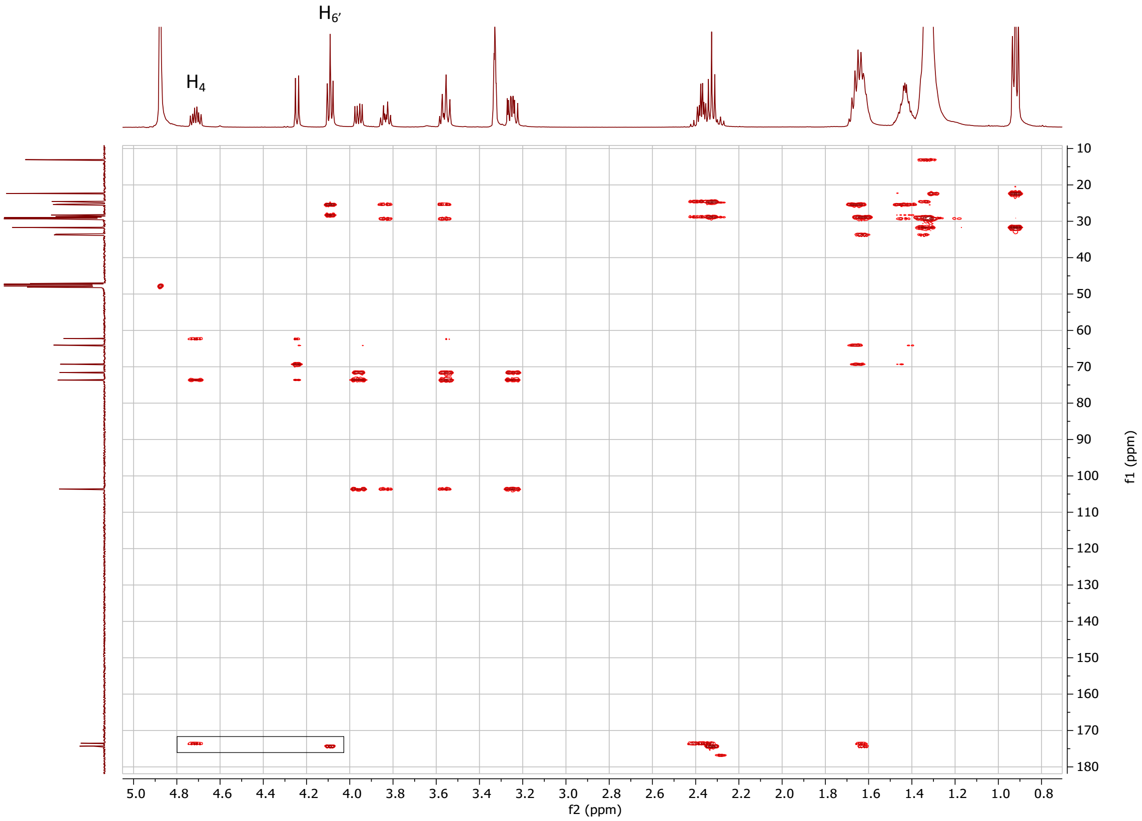
H$_{6'}$
H$_4$
f2 (ppm)
f1 (ppm)

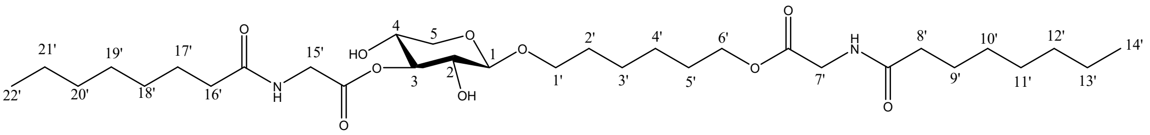

21'
19'
17'
O
15'
4
HO
5
O
1
O
2'
4'
6'
O
H
N
8'
10'
12'
14'
22'
20'
18'
16'
N
H
O
3
2
OH
1'
3'
5'
O
7'
9'
11'
13'
O
O

5e

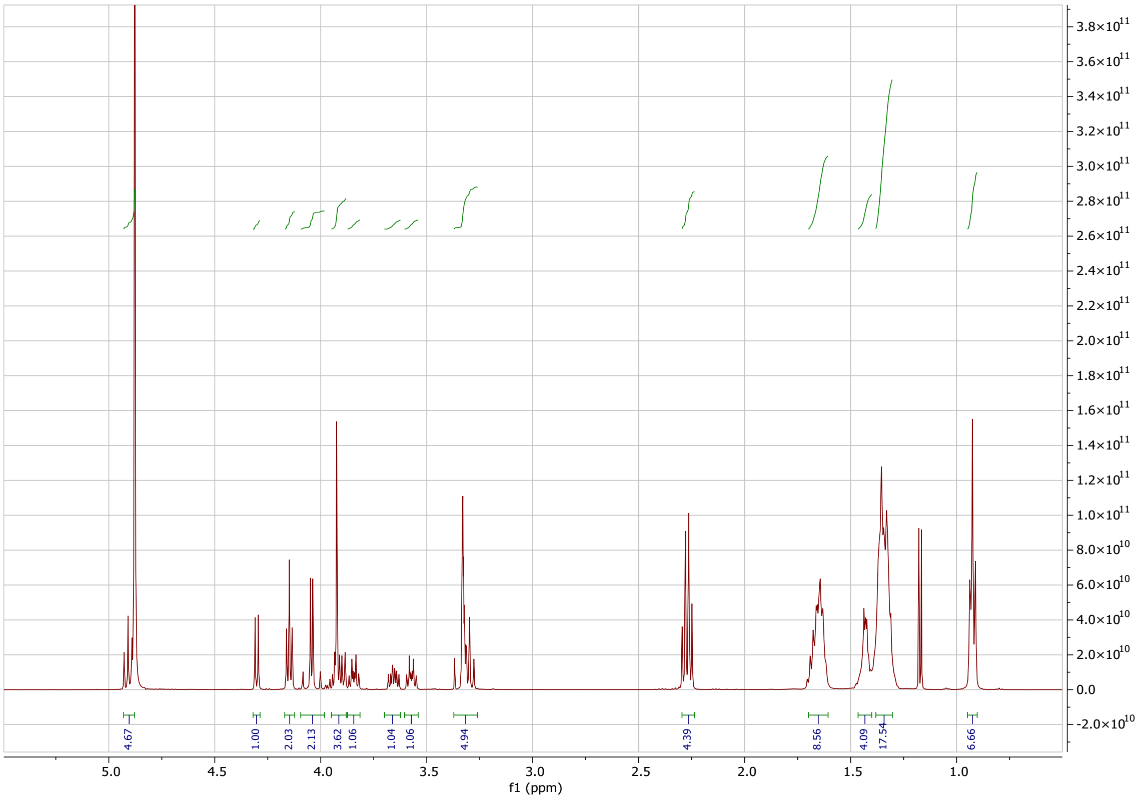

4.67
1.00
2.03
2.13
3.62
1.06
1.04
1.06
4.94
4.39
8.56
4.09
17.54
6.66
f1 (ppm)

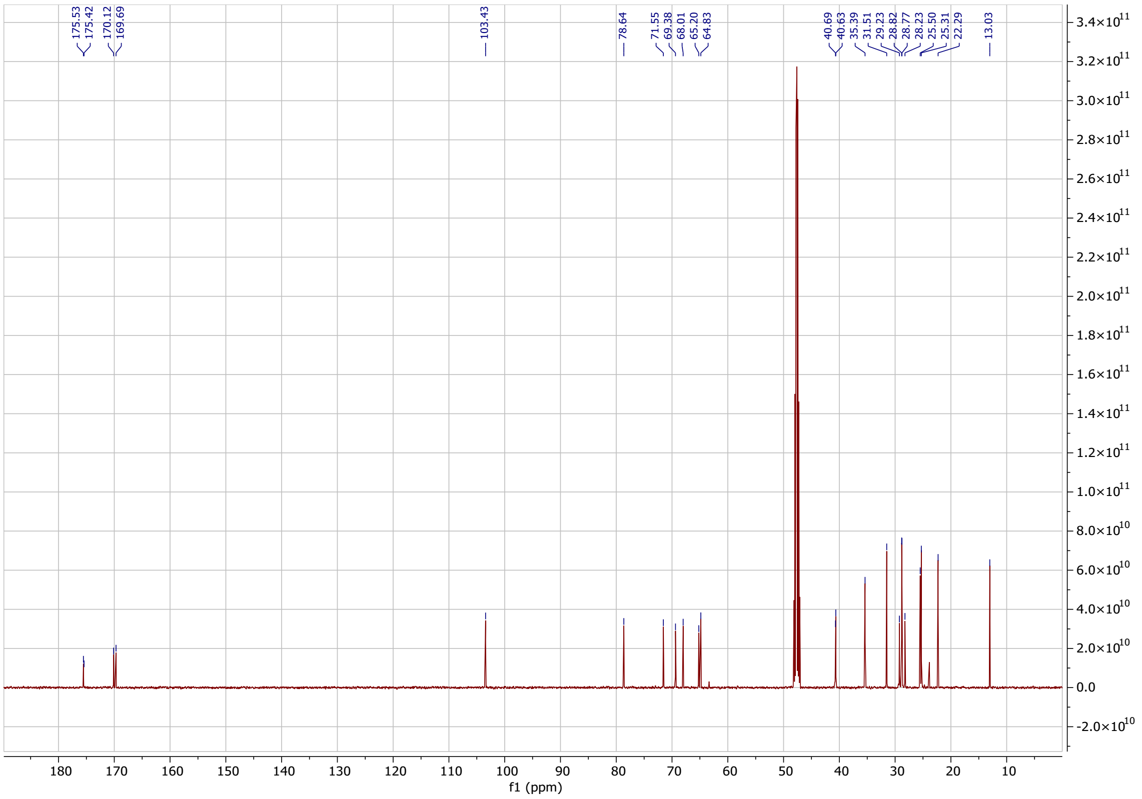

175.53
175.42
170.12
169.69
103.43
78.64
71.55
69.38
68.01
65.20
64.83
40.69
40.63
35.39
31.51
29.23
28.82
28.77
28.23
25.50
25.31
22.29
13.03
f1 (ppm)

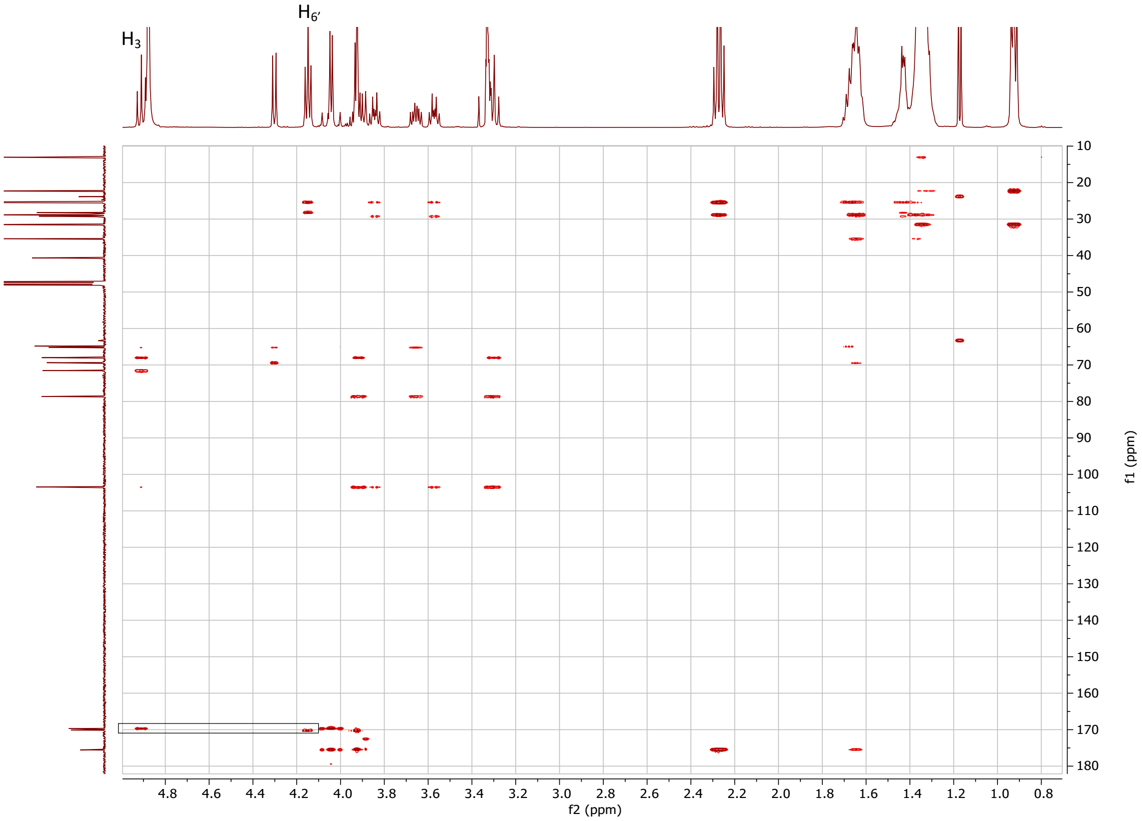

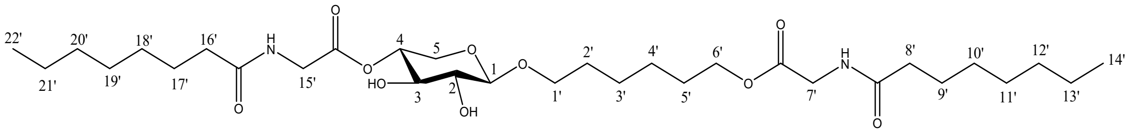

6e

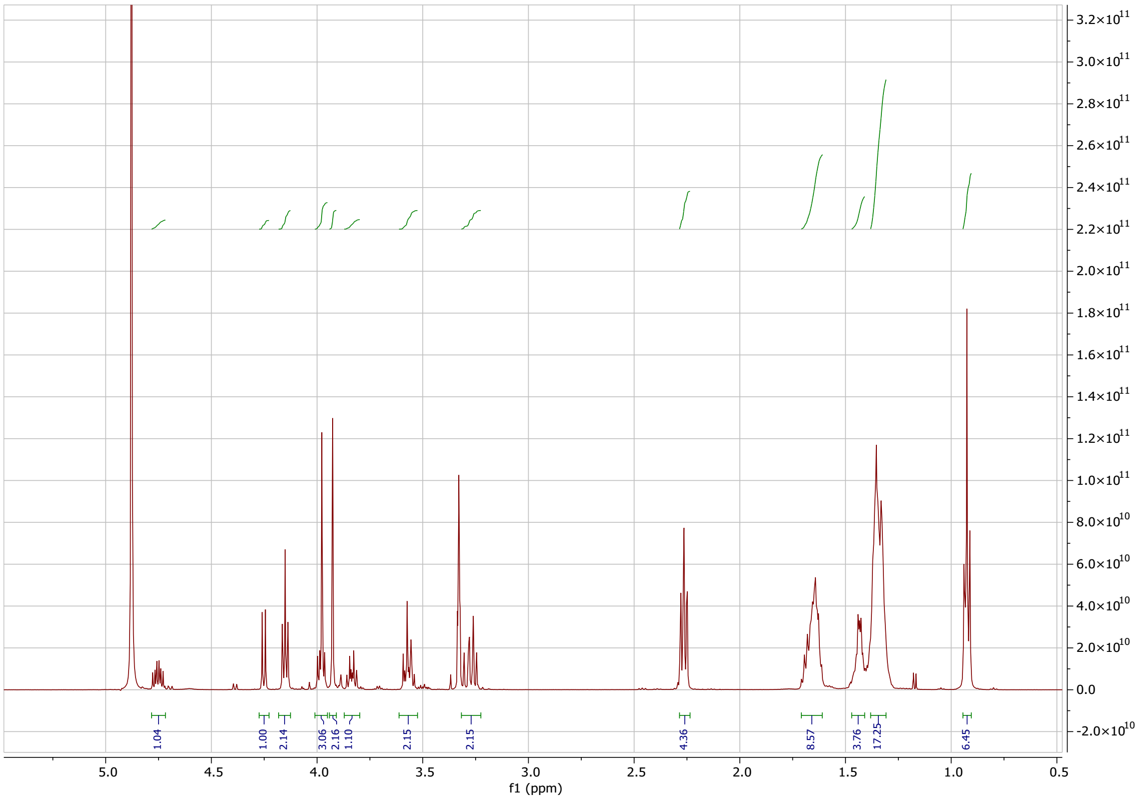

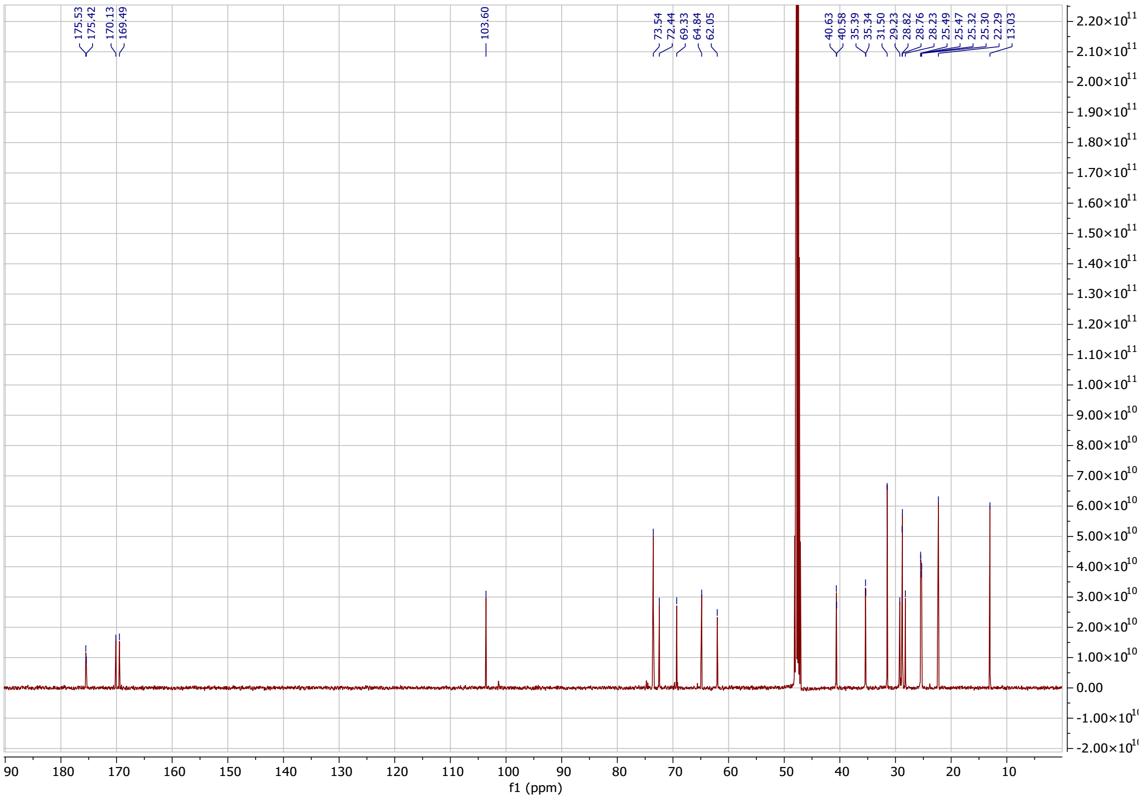
175.53
175.42
170.13
169.49
103.60
73.54
72.44
69.33
64.84
62.05
40.63
40.58
35.39
35.34
31.50
29.23
28.82
28.76
28.23
25.49
25.47
25.32
25.30
22.29
13.03
f1 (ppm)

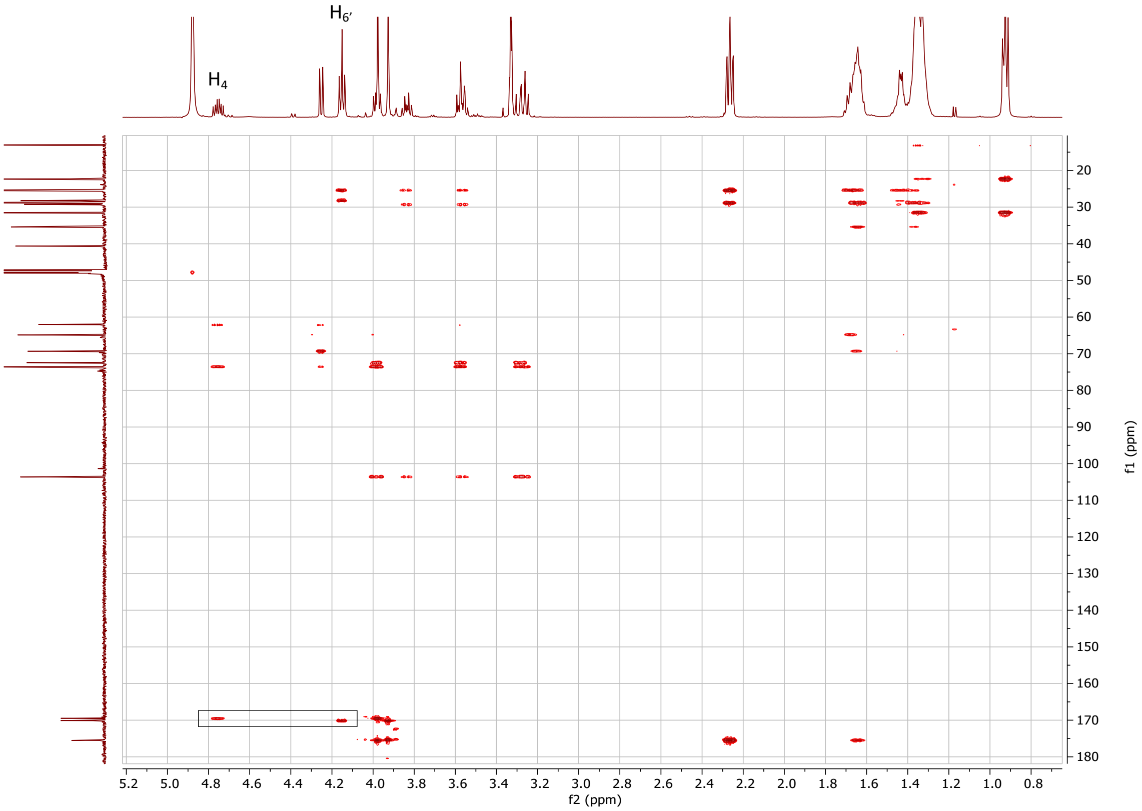
H$_{6'}$
H$_4$
f2 (ppm)
f1 (ppm)

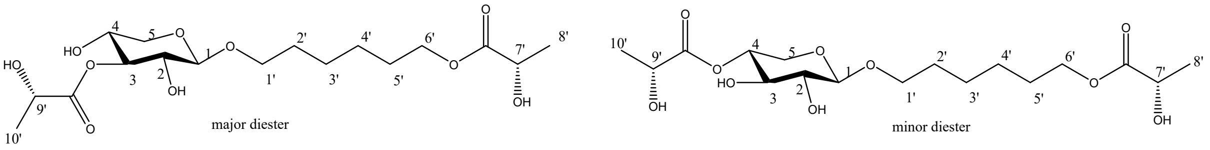
major diester
minor diester

5d and 6d

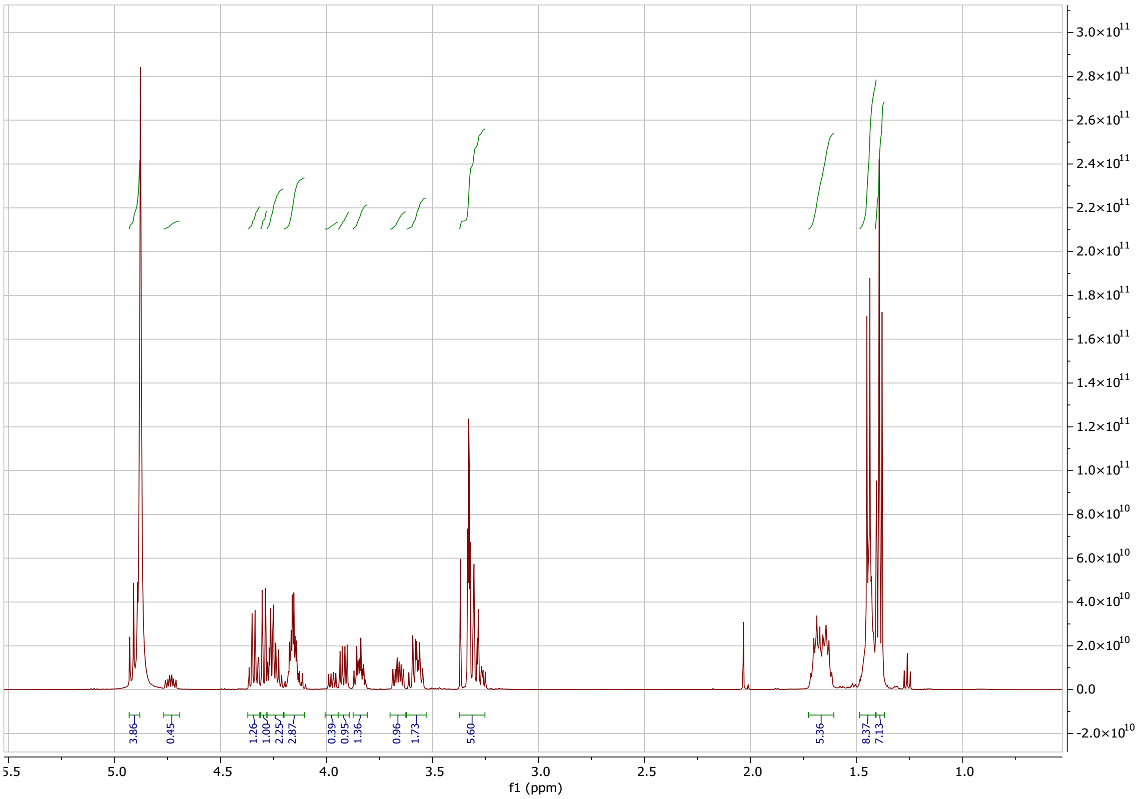
3.86
0.45
1.26
1.00
2.25
2.87
0.39
0.95
1.36
0.96
1.73
5.60
5.36
8.37
7.13
f1 (ppm)

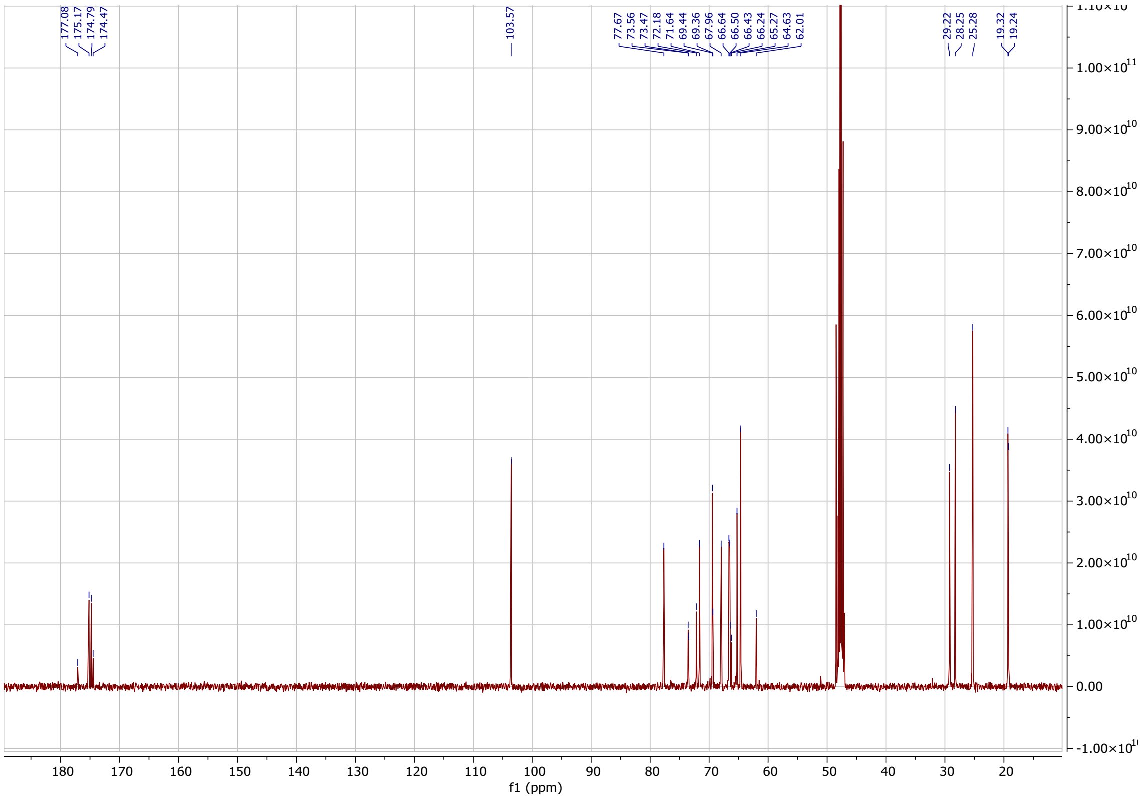
177.08
175.17
174.79
174.47
103.57
77.67
73.56
73.47
72.18
71.64
69.44
69.36
67.96
66.64
66.50
66.43
66.24
65.27
64.63
62.01
29.22
28.25
25.28
19.32
19.24
f1 (ppm)

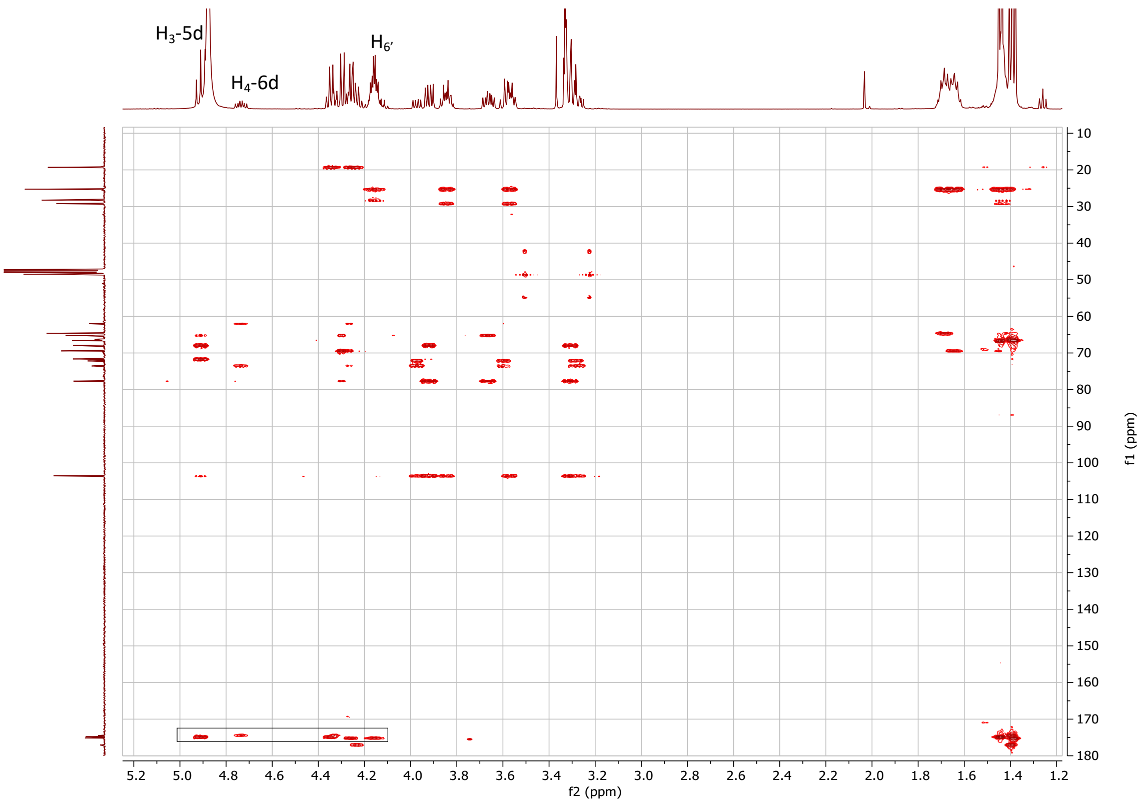

H3-5d
H4-6d
H6′
f1 (ppm)
f2 (ppm)

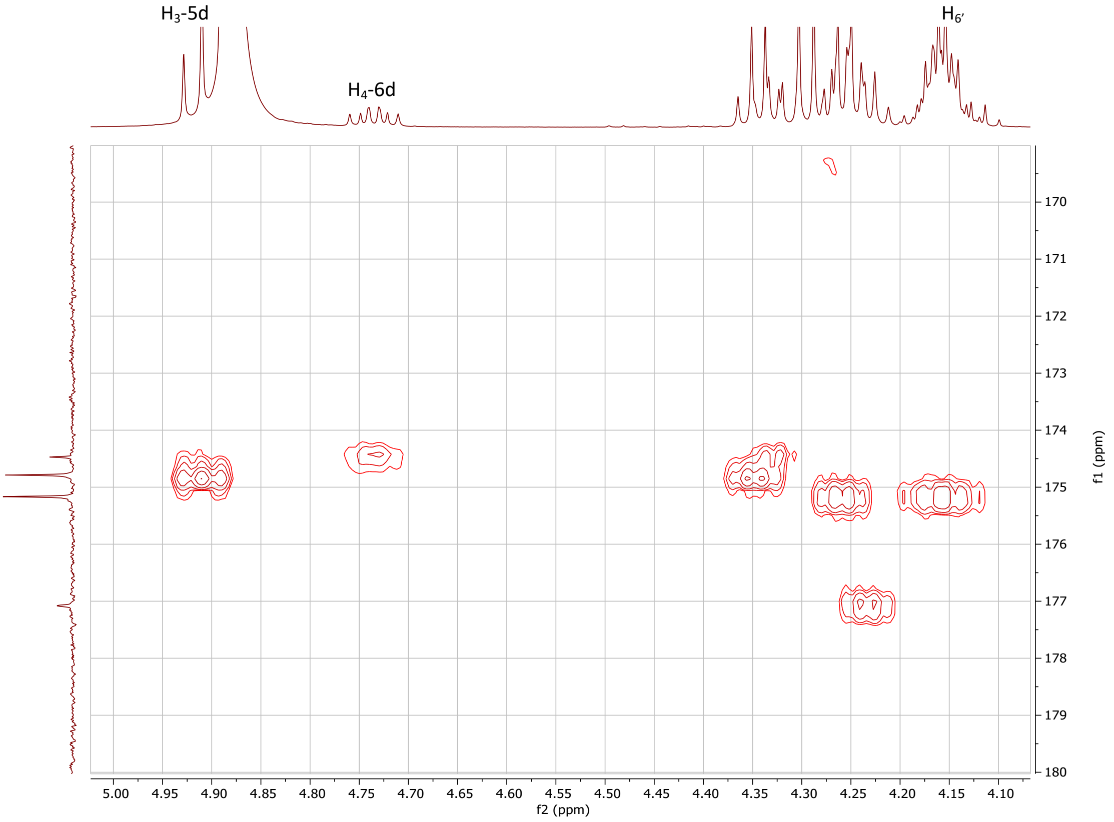

H3-5d
H4-6d
H6′
f1 (ppm)
f2 (ppm)

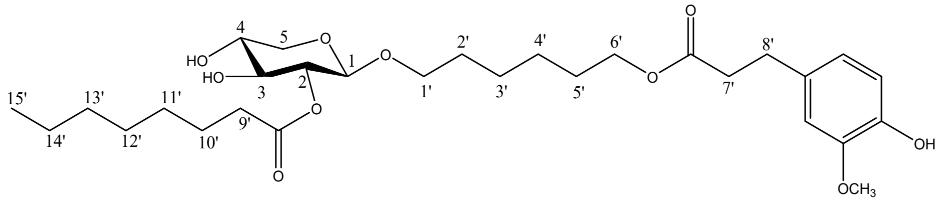

7 (+ ε octanoic acid)

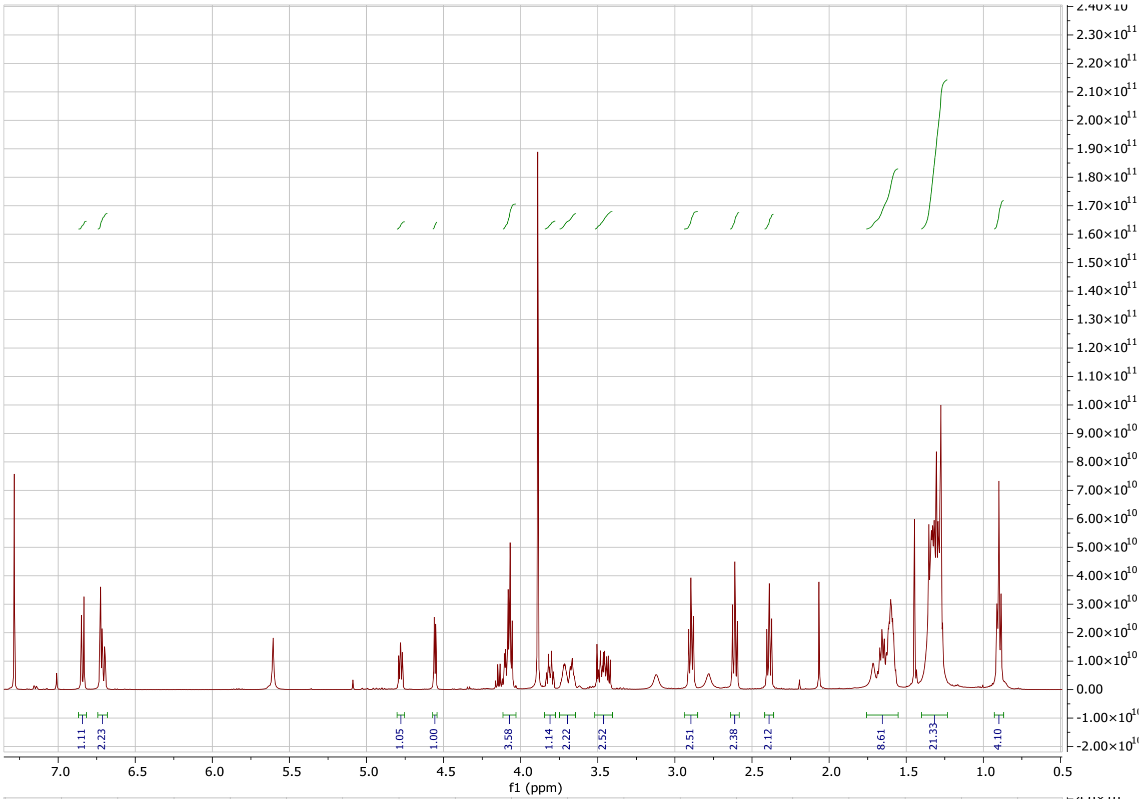

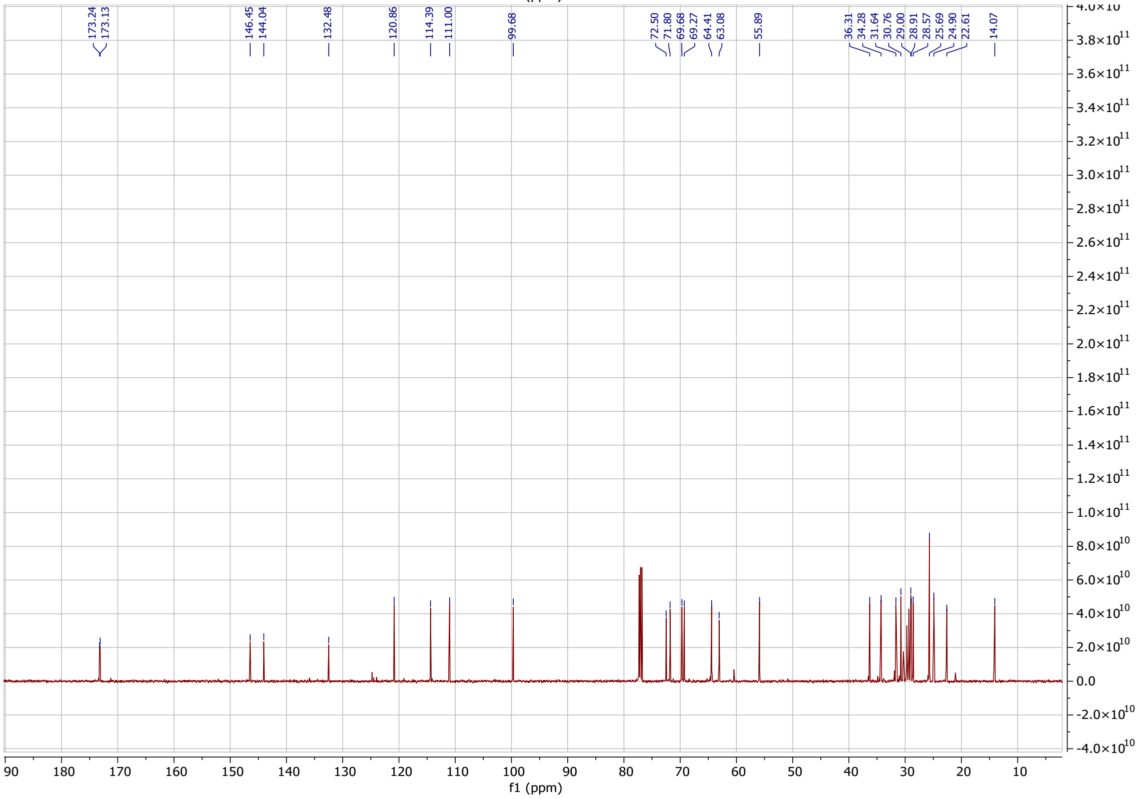

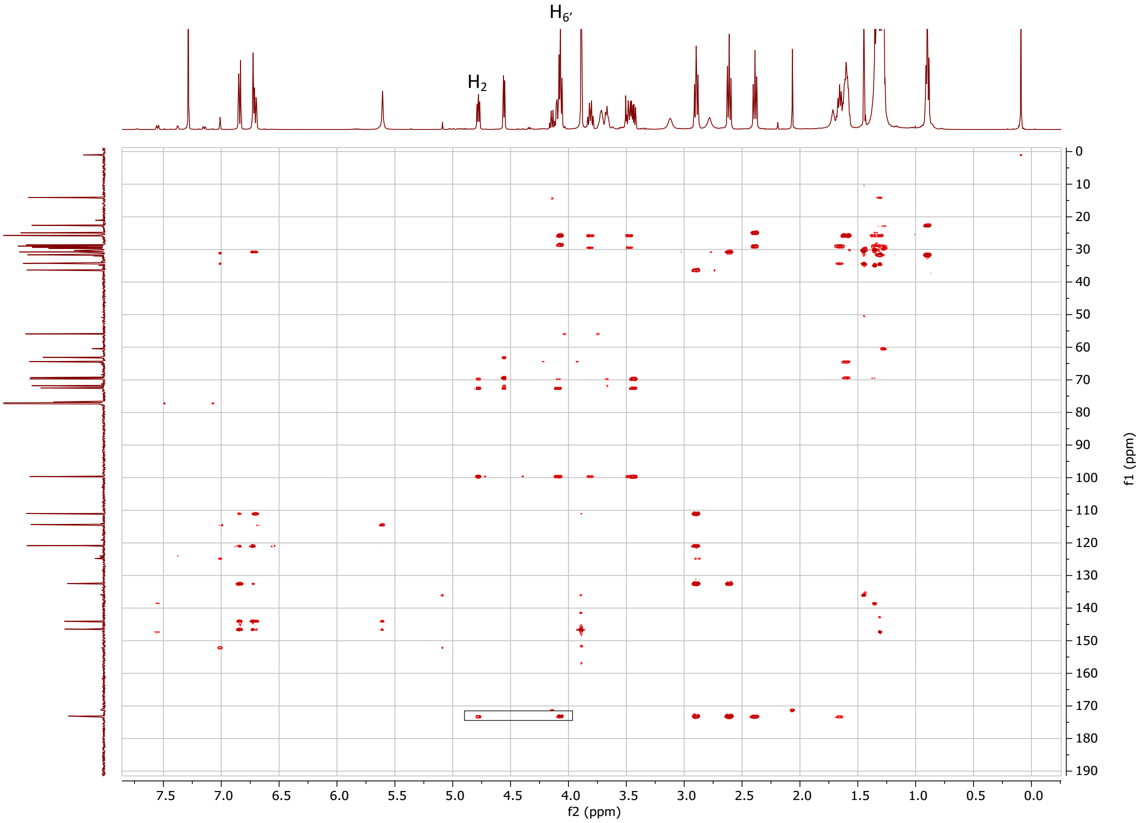
H6′
H2
f1 (ppm)
f2 (ppm)

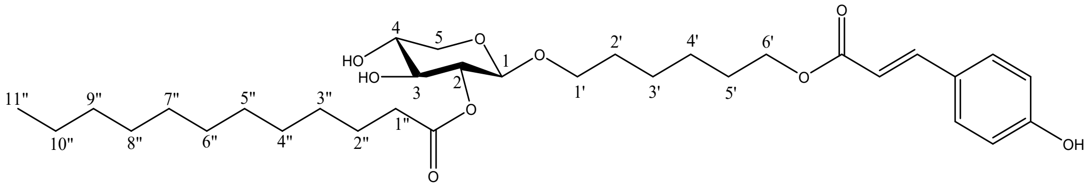
HO
HO
O
OH
1
2
3
4
5
1'
2'
3'
4'
5'
6'
1"
2"
3"
4"
5"
6"
7"
8"
9"
10"
11"

8f

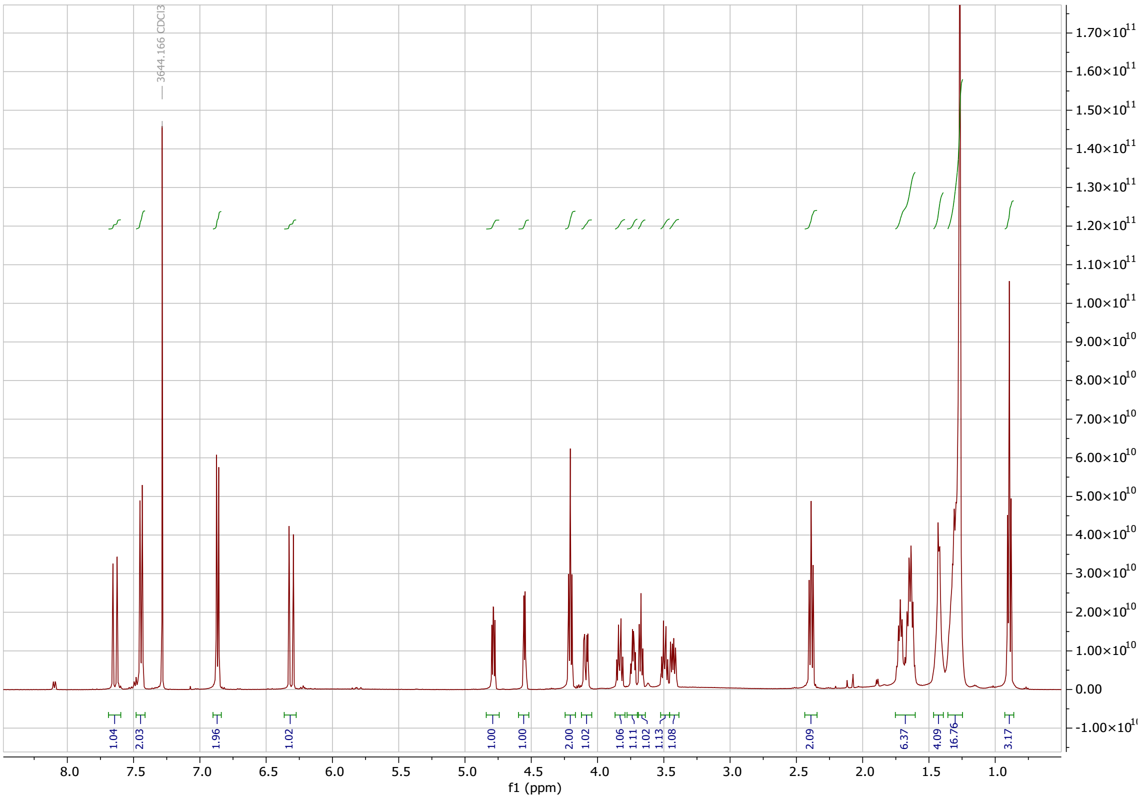
3644.166 CDCl3
f1 (ppm)

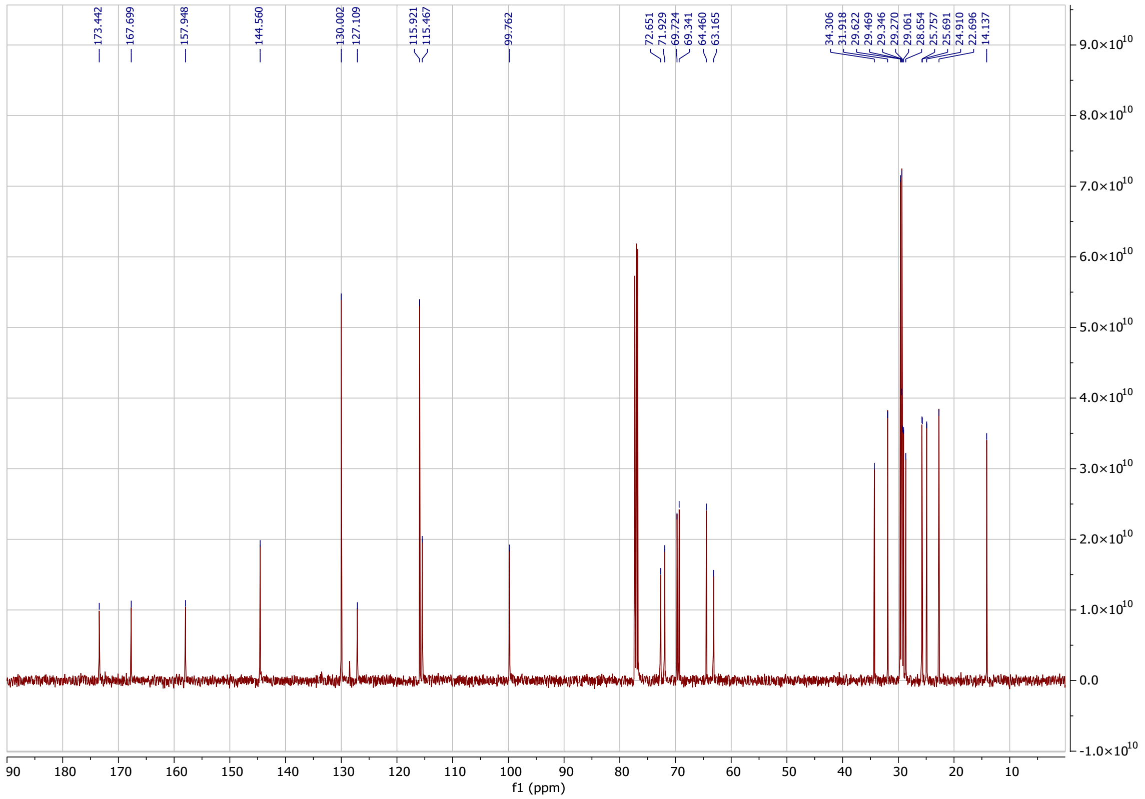
173.442
167.699
157.948
144.560
130.002
127.109
115.921
115.467
99.762
72.651
71.929
69.724
69.341
64.460
63.165
34.306
31.918
29.622
29.469
29.346
29.270
29.061
28.654
25.757
25.691
24.910
22.696
14.137
f1 (ppm)

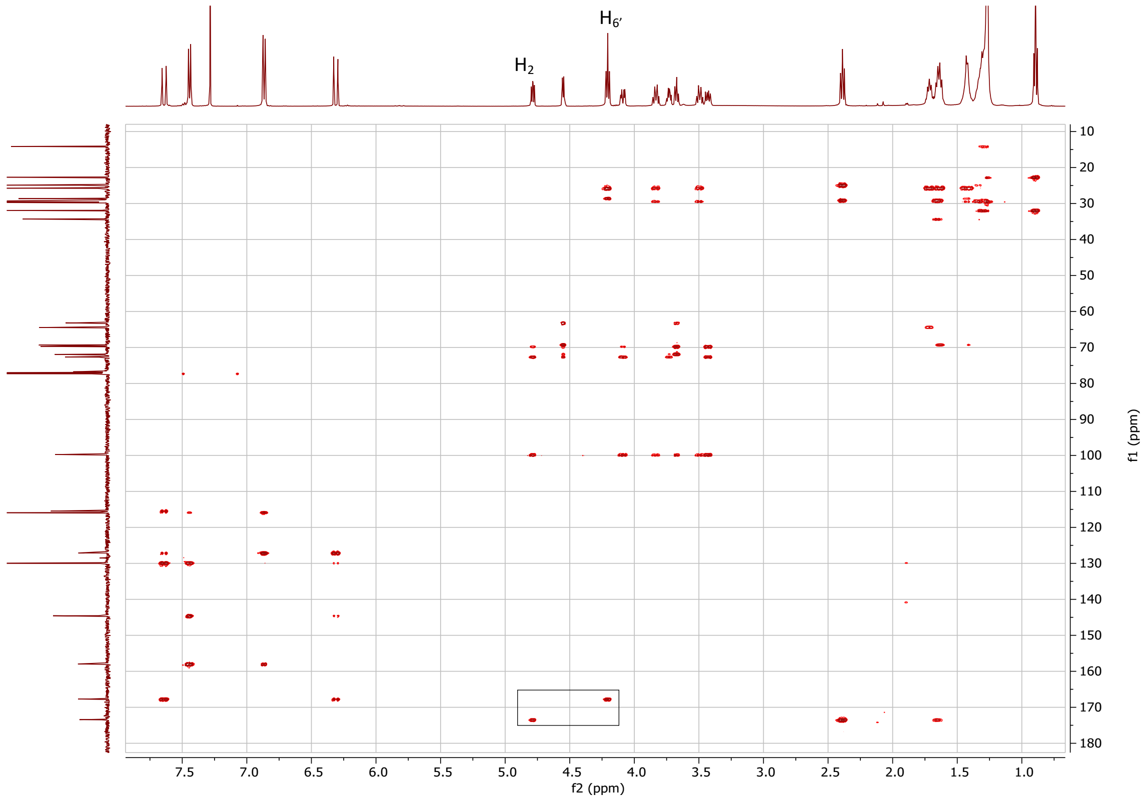
H6′
H2
f2 (ppm)
f1 (ppm)

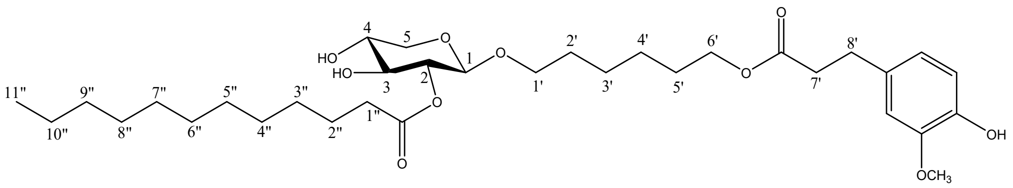

8g

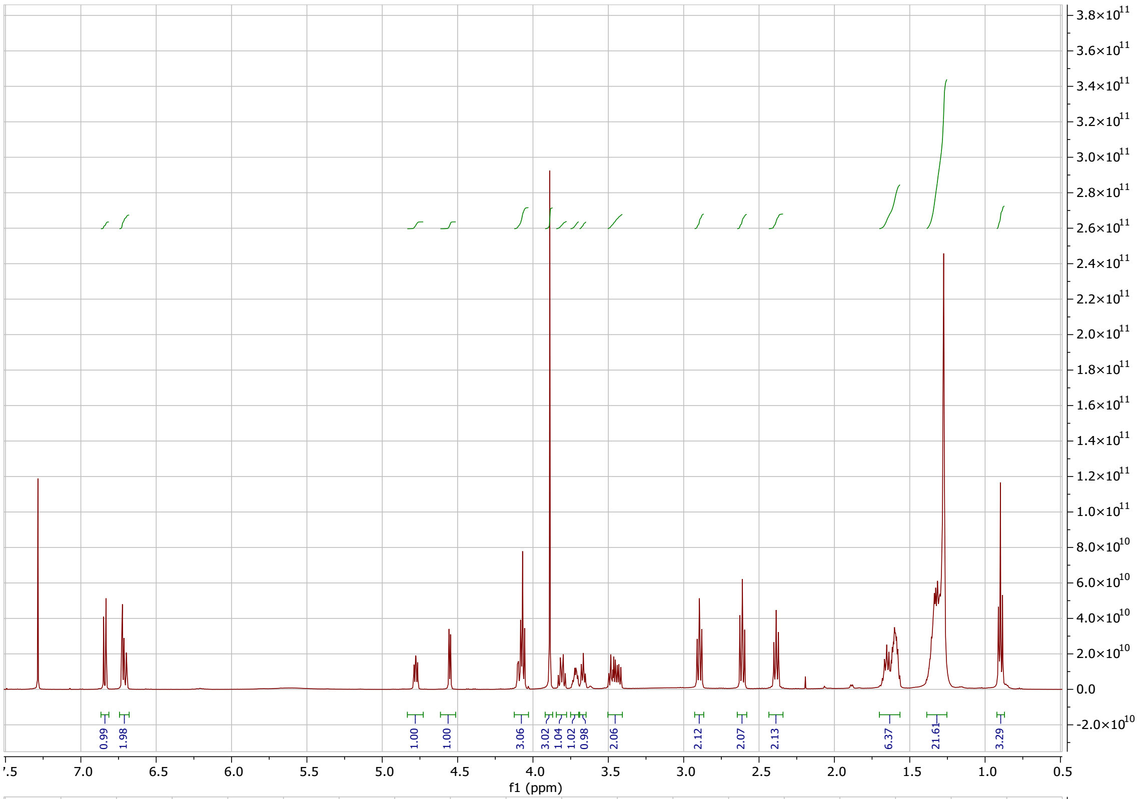
0.99
1.98
1.00
1.00
3.06
3.02
1.04
1.02
0.98
2.06
2.12
2.07
2.13
6.37
21.61
3.29
f1 (ppm)

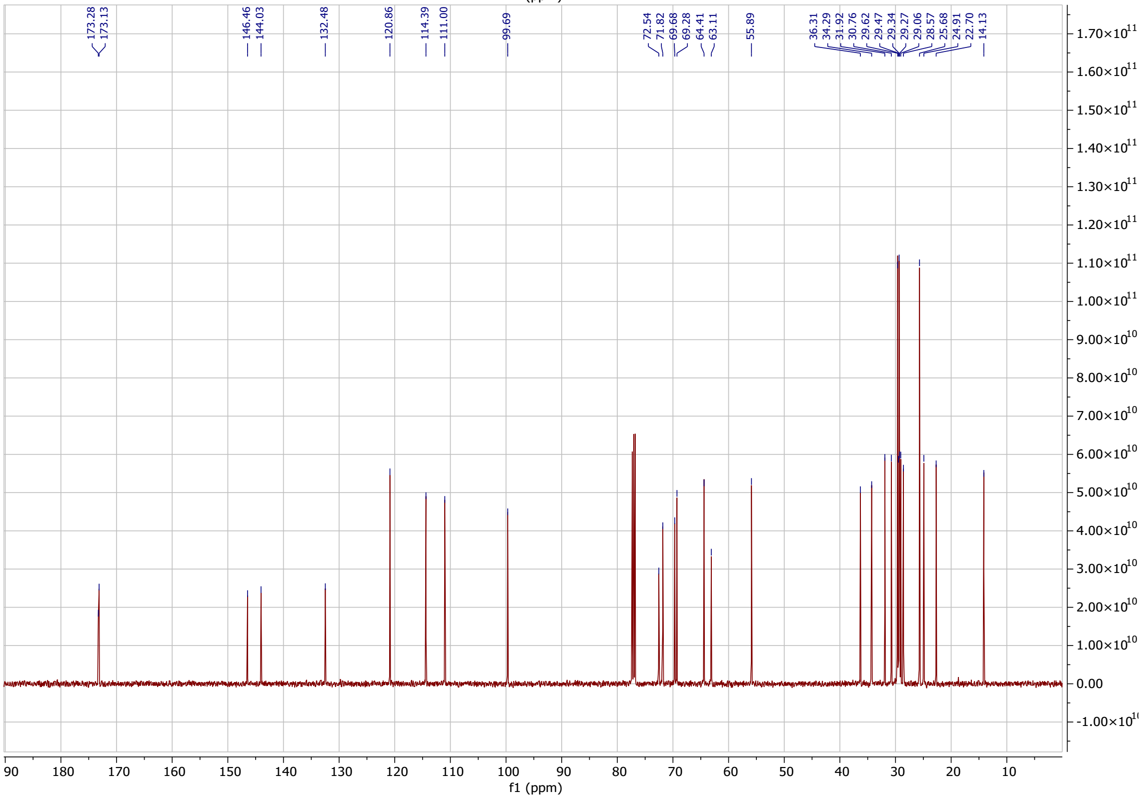
173.28
173.13
146.46
144.03
132.48
120.86
114.39
111.00
99.69
72.54
71.82
69.68
69.28
64.41
63.11
55.89
36.31
34.29
31.92
30.76
29.62
29.47
29.34
29.27
29.06
28.57
25.68
24.91
22.70
14.13
f1 (ppm)

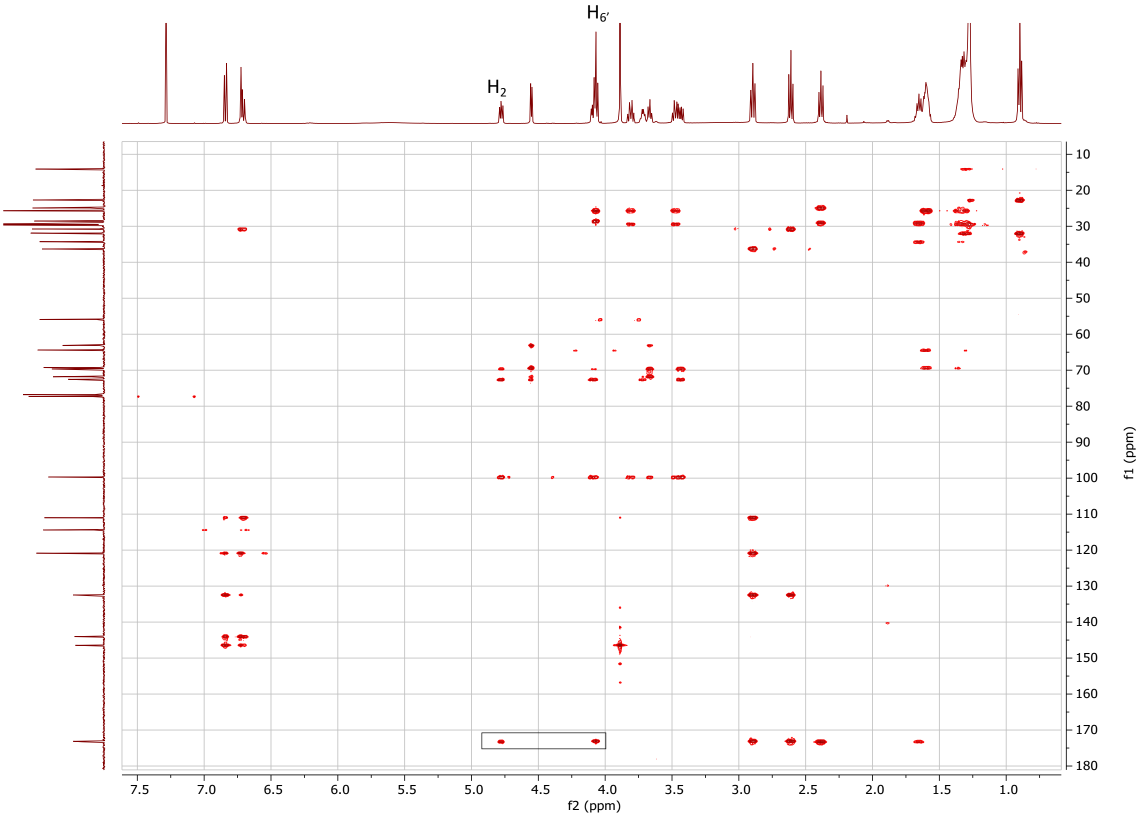

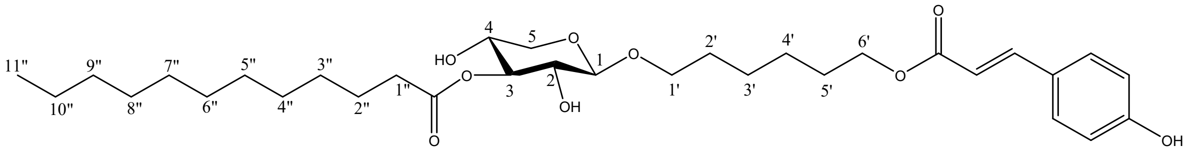

9f

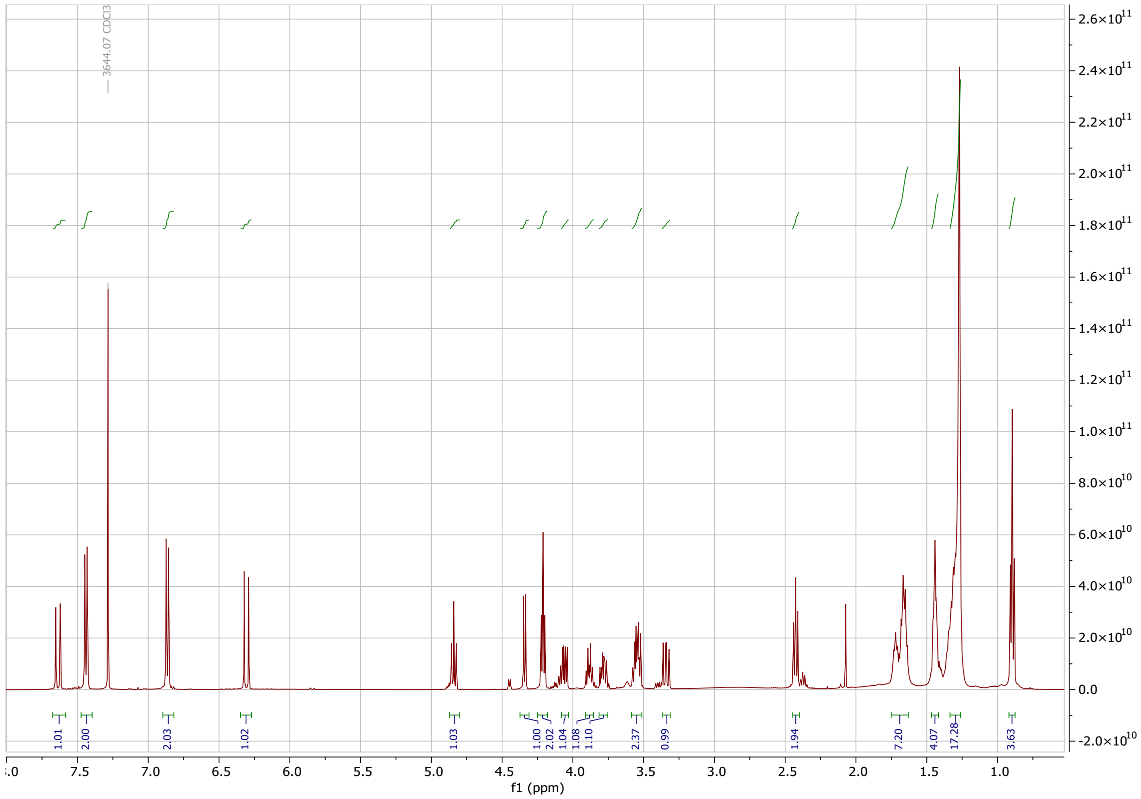

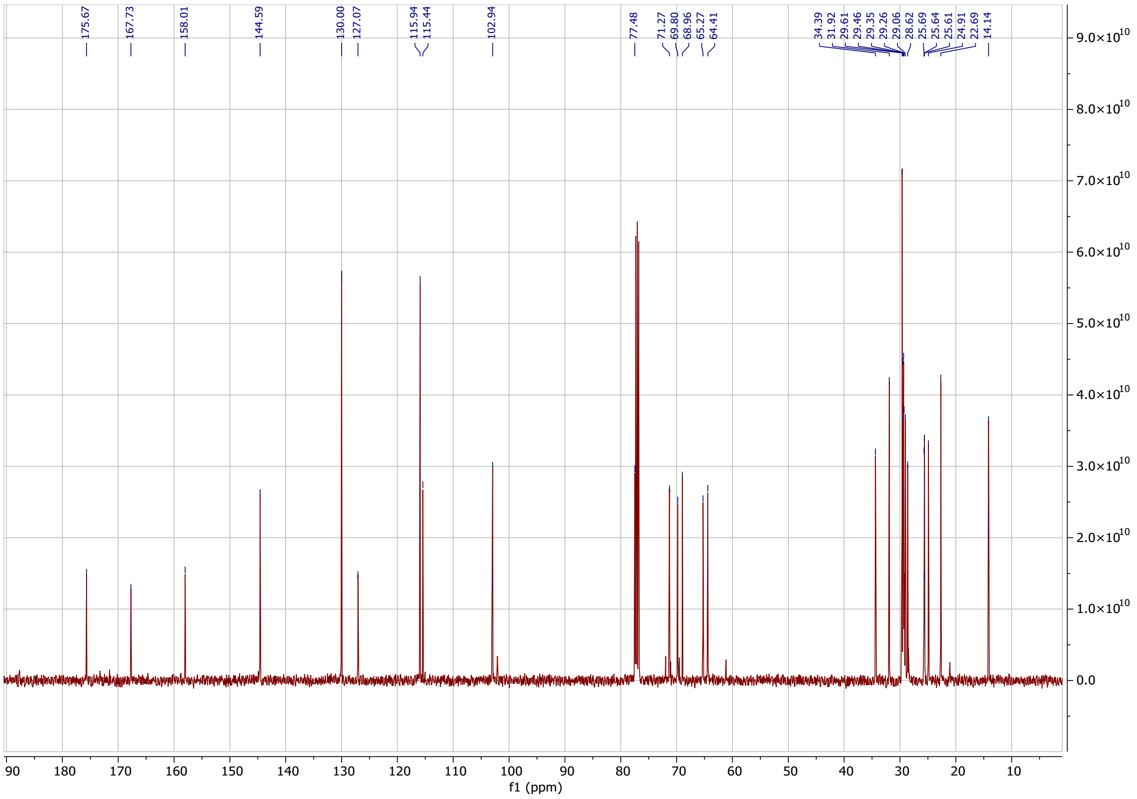
175.67
167.73
158.01
144.59
130.00
127.07
115.94
115.44
102.94
77.48
71.27
69.80
68.96
65.27
64.41
34.39
31.92
29.61
29.46
29.35
29.26
29.06
28.62
25.69
25.64
25.61
24.91
22.69
14.14
f1 (ppm)

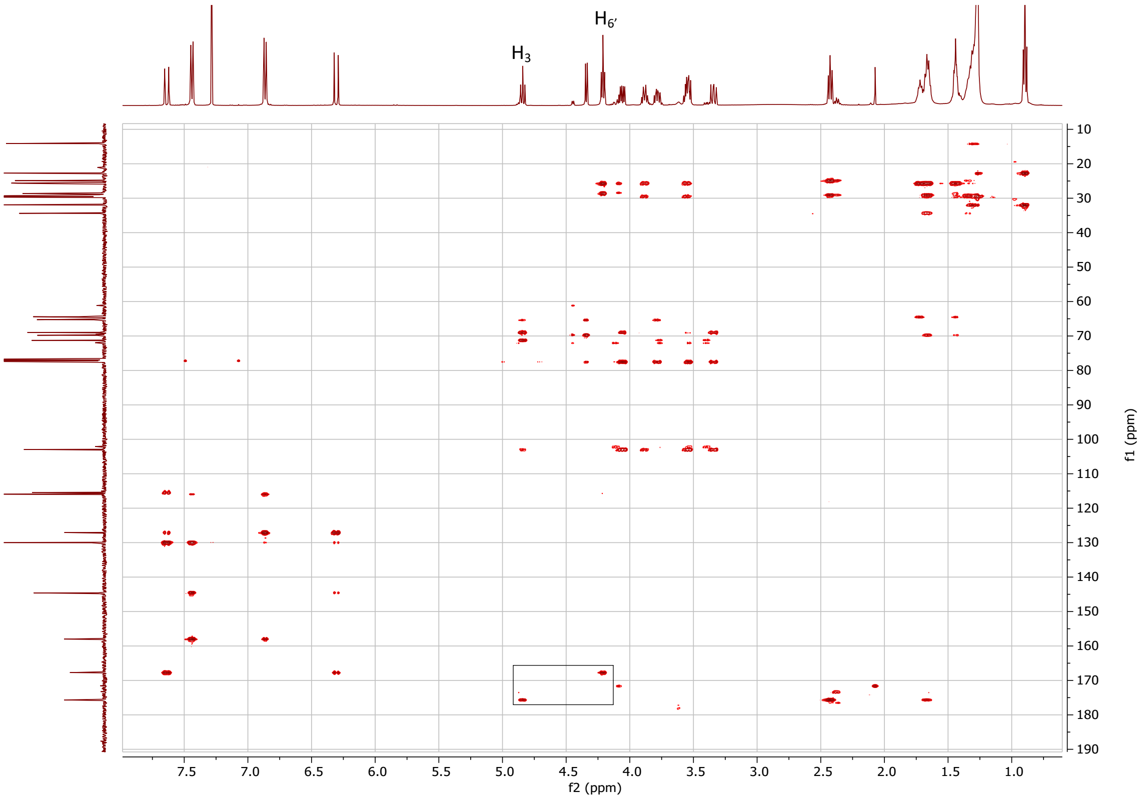
H3
H6′
f2 (ppm)
f1 (ppm)

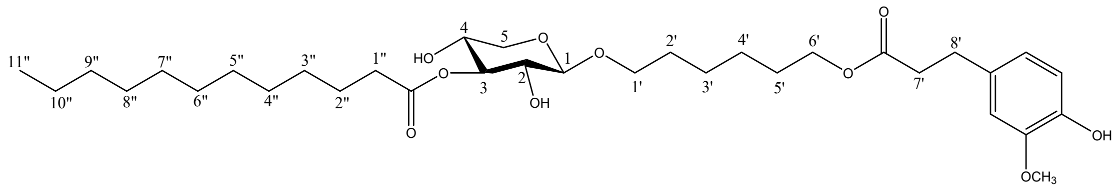

9g (+ ε 1,4 diester 8g)

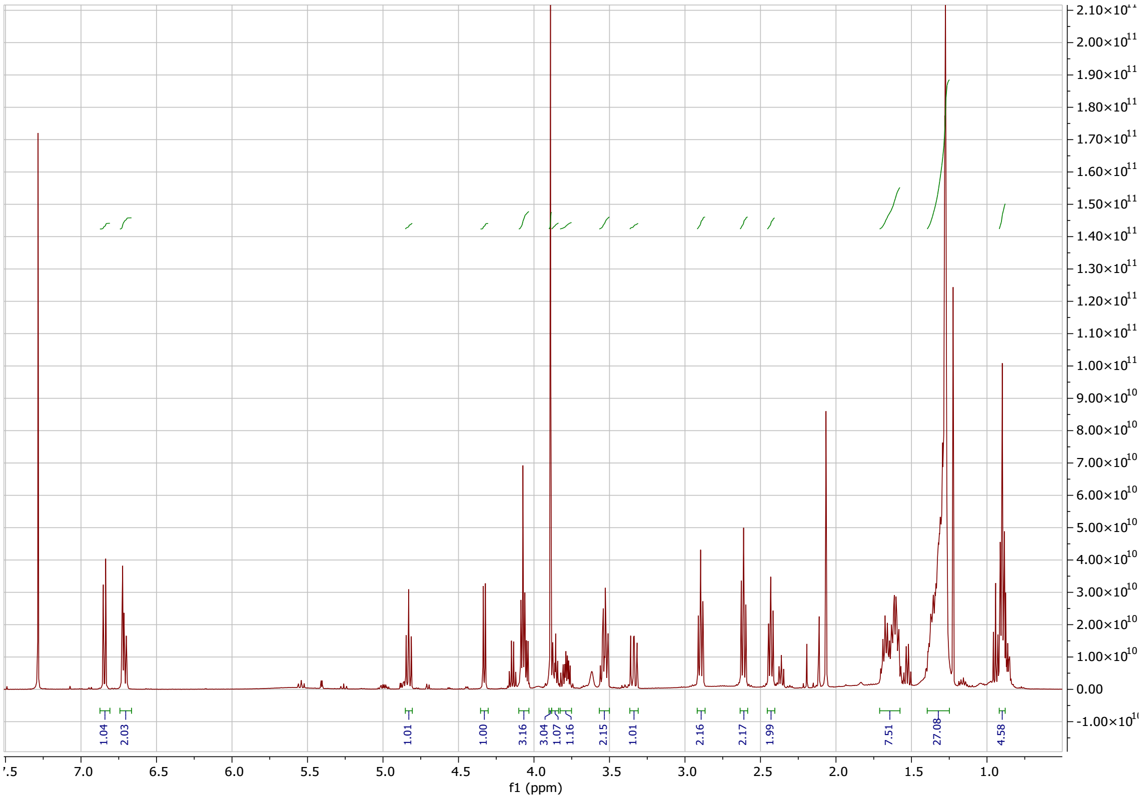

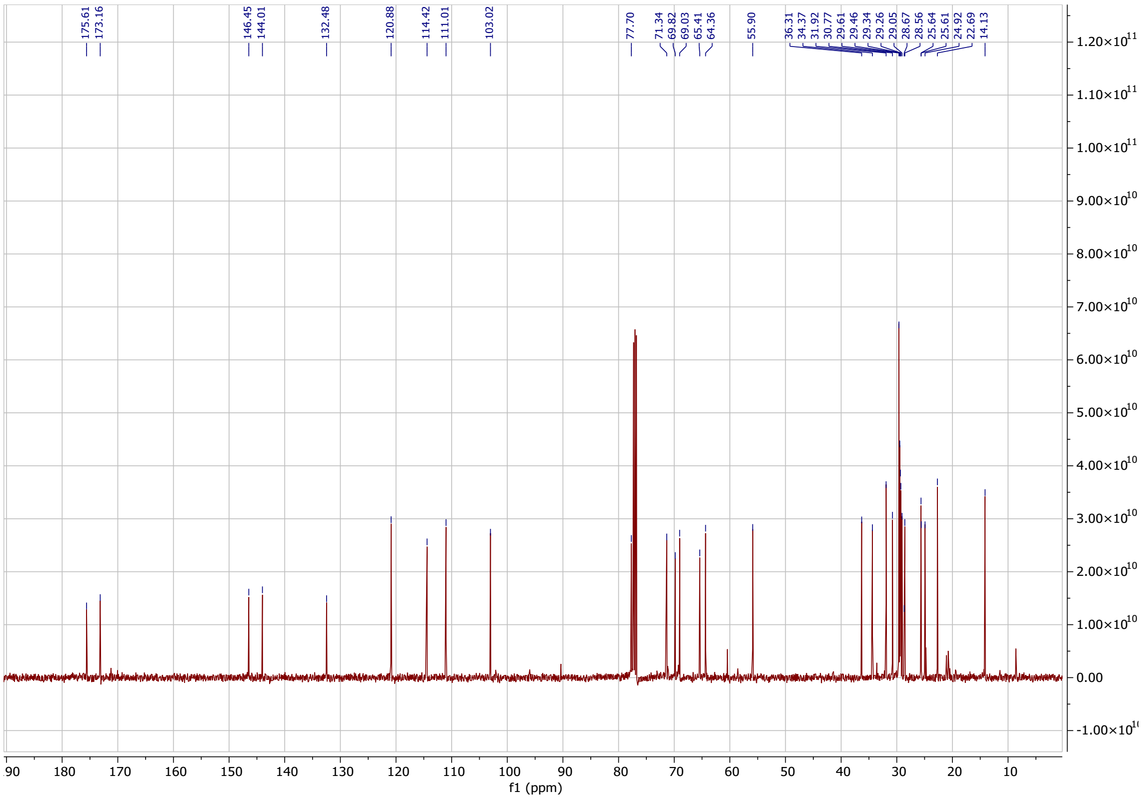

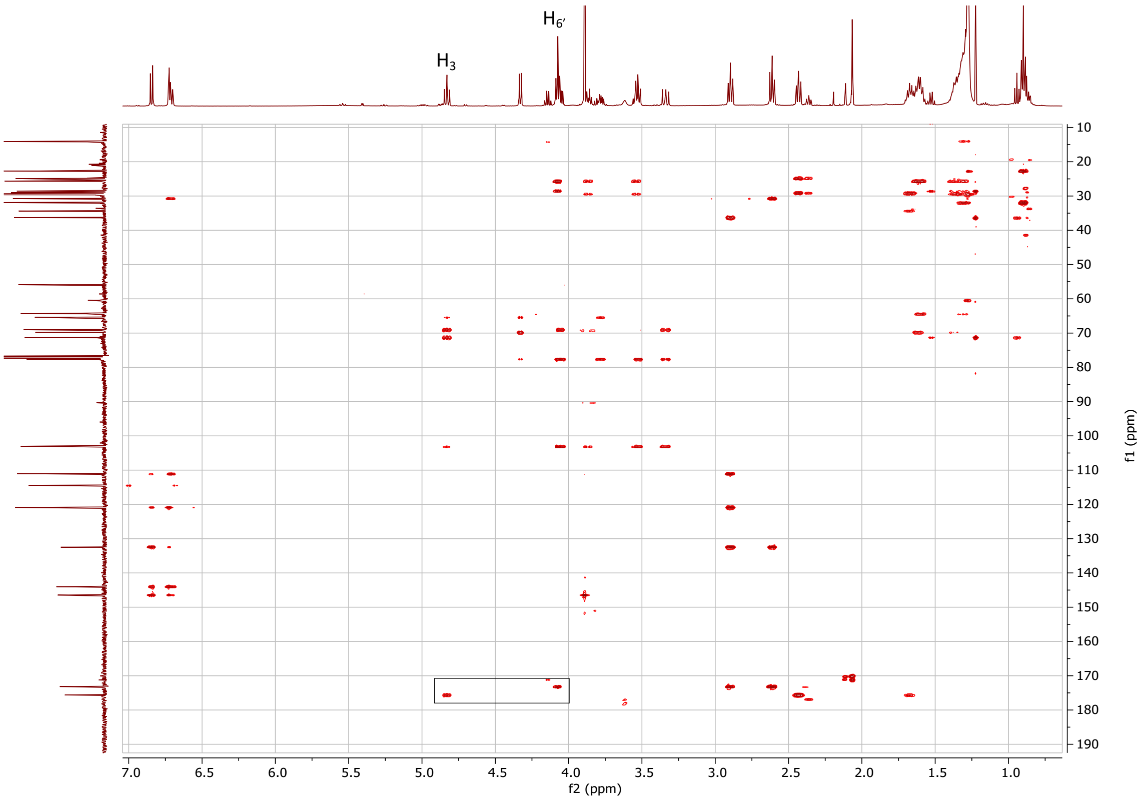

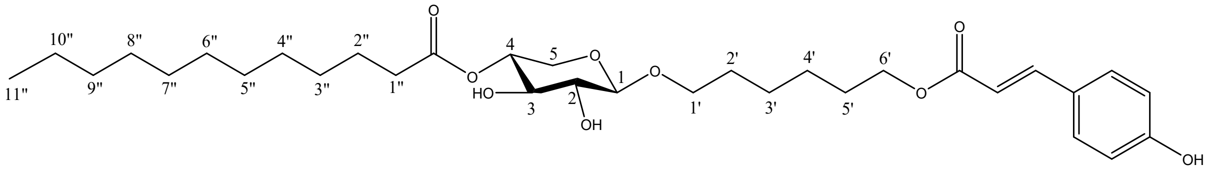

10f

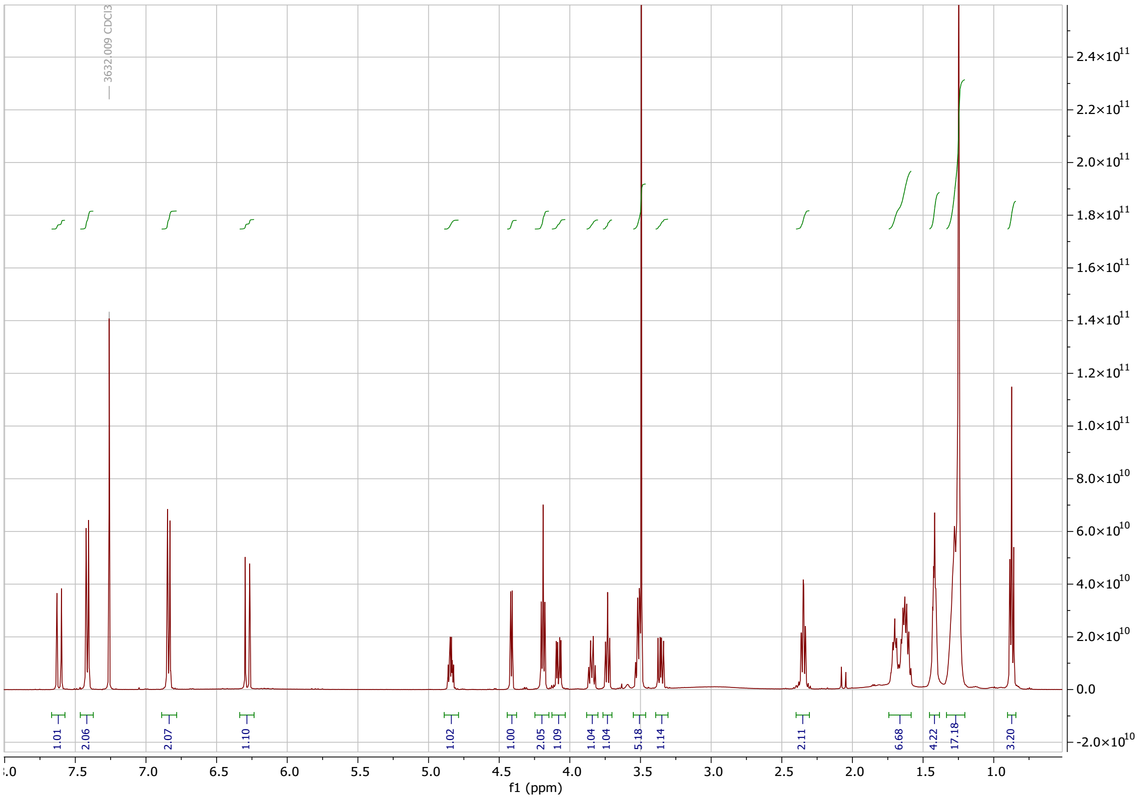
3632.009 CDCl3
1.01
2.06
2.07
1.10
1.02
1.00
2.05
1.09
1.04
1.04
5.18
1.14
2.11
6.68
4.22
17.18
3.20
f1 (ppm)

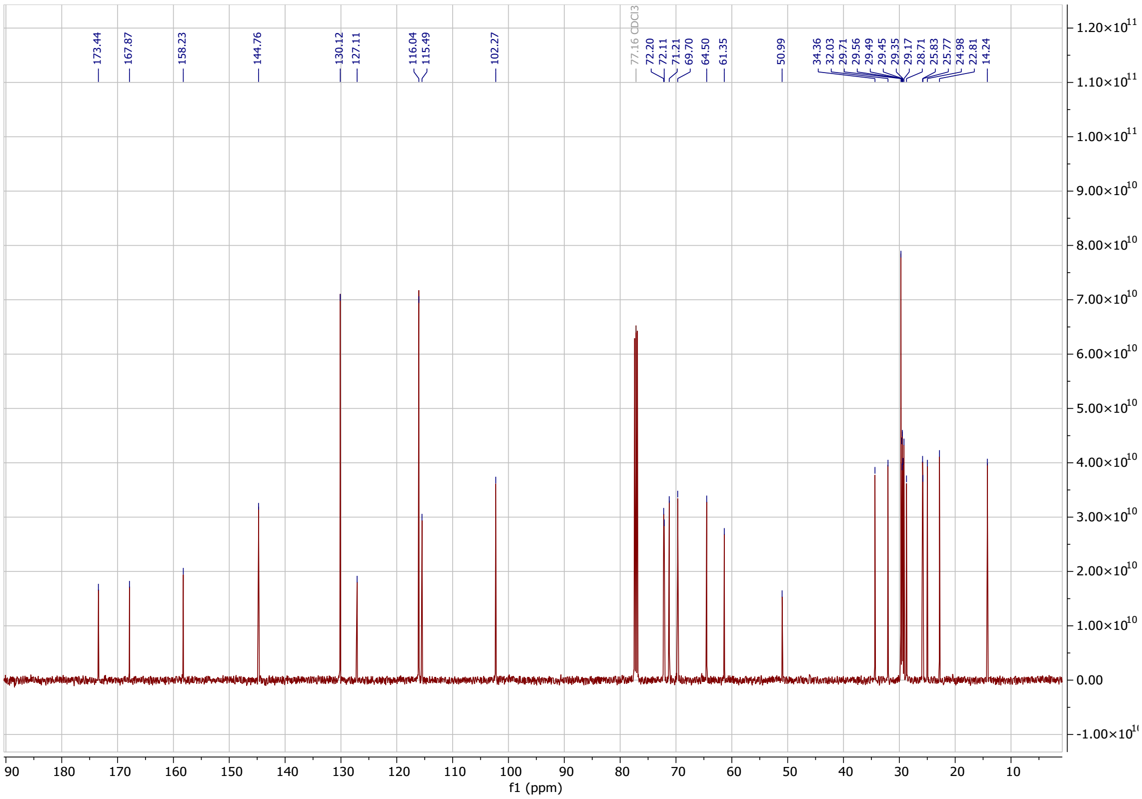
173.44
167.87
158.23
144.76
130.12
127.11
116.04
115.49
102.27
77.16 CDCl3
72.20
72.11
71.21
69.70
64.50
61.35
50.99
34.36
32.03
29.71
29.56
29.49
29.45
29.35
29.17
28.71
25.83
25.77
24.98
22.81
14.24
f1 (ppm)

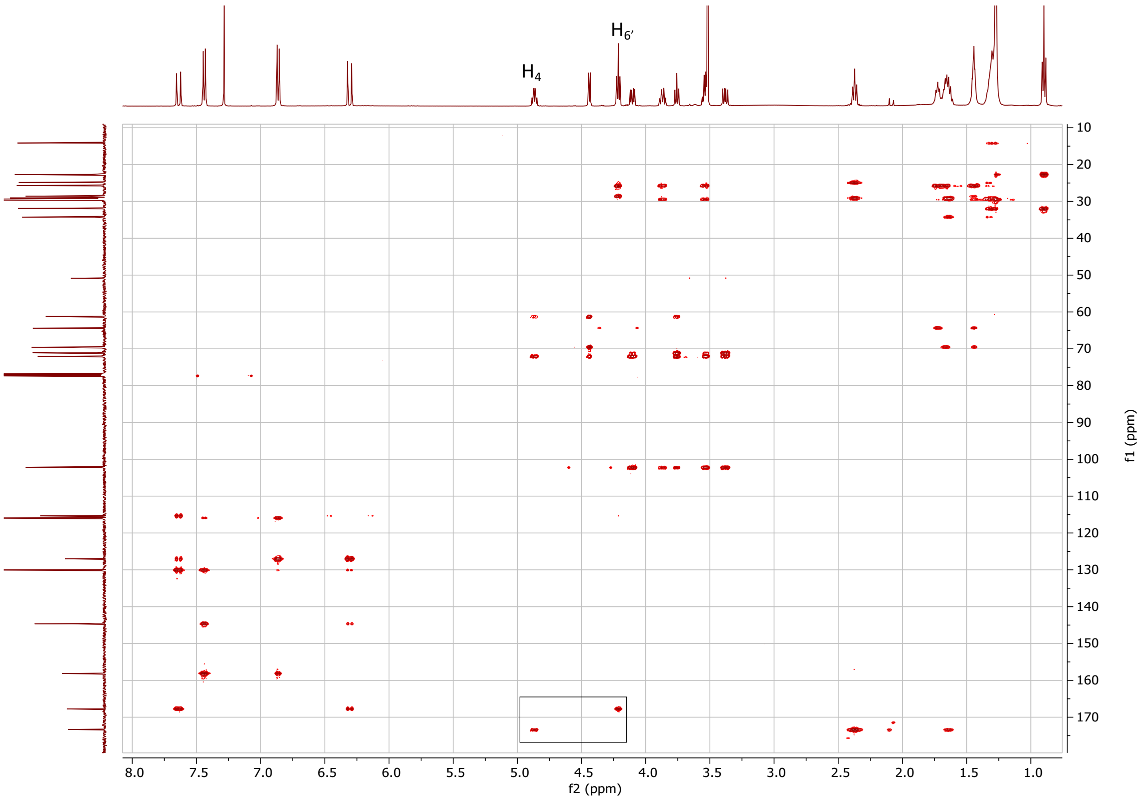

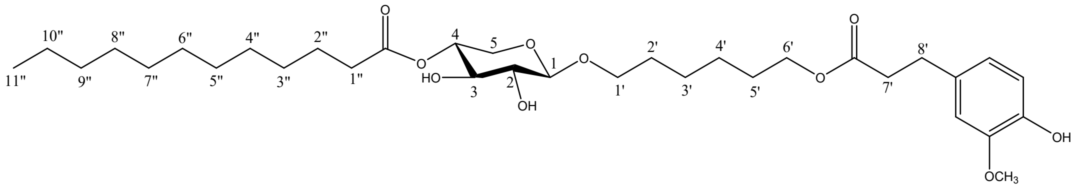

10g

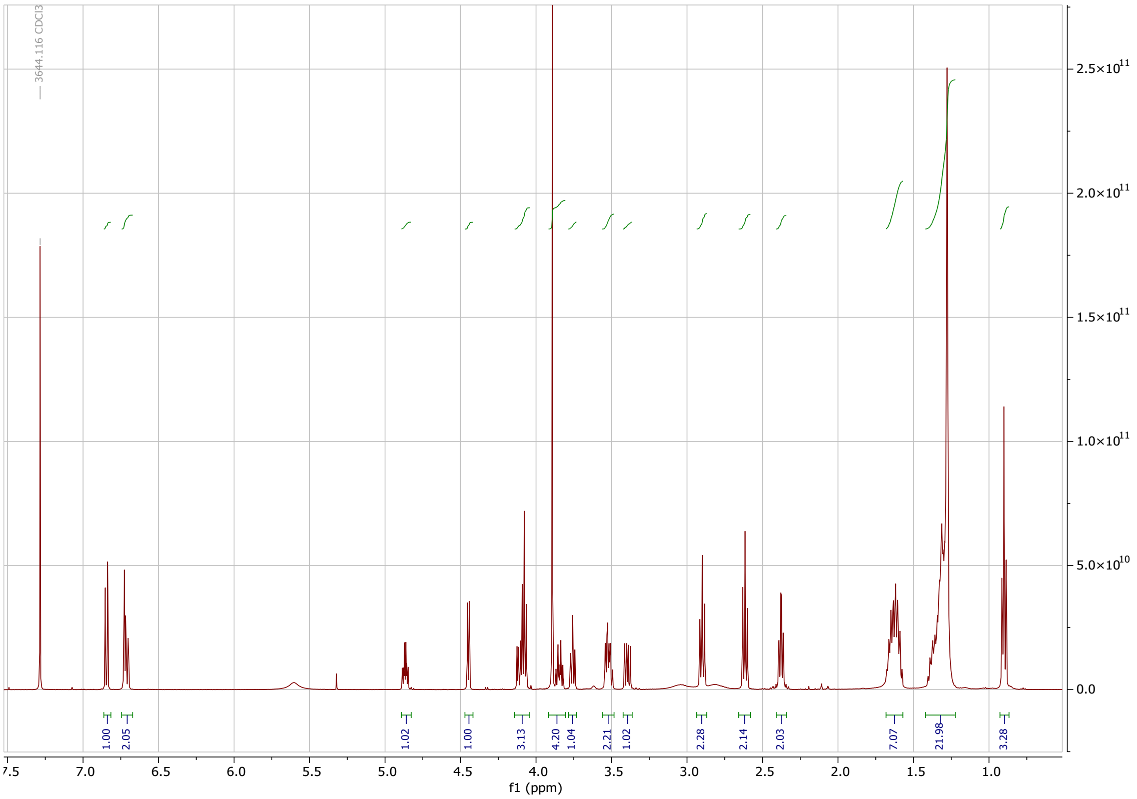
3644.116 CDCl3
1.00
2.05
1.02
1.00
3.13
4.20
1.04
2.21
1.02
2.28
2.14
2.03
7.07
21.98
3.28
f1 (ppm)

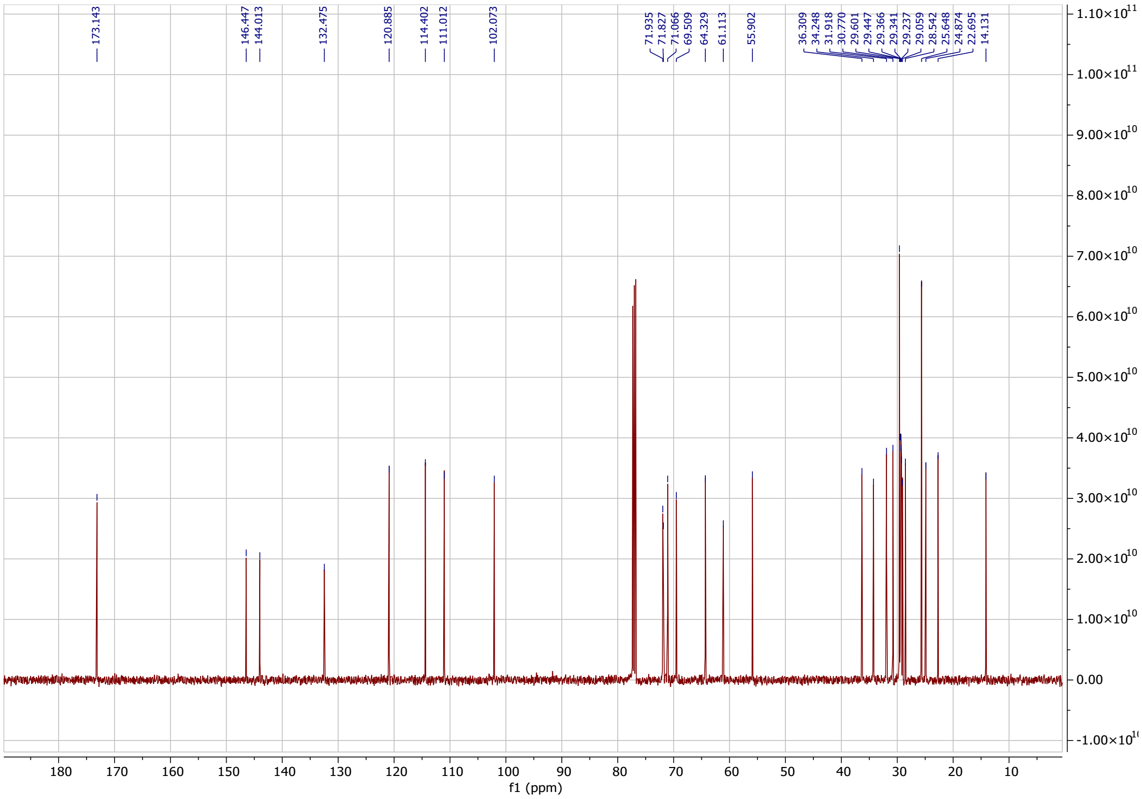
173.143
146.447
144.013
132.475
120.885
114.402
111.012
102.073
71.935
71.827
71.066
69.509
64.329
61.113
55.902
36.309
34.248
31.918
30.770
29.601
29.447
29.366
29.341
29.237
29.059
28.542
25.648
24.874
22.695
14.131
f1 (ppm)

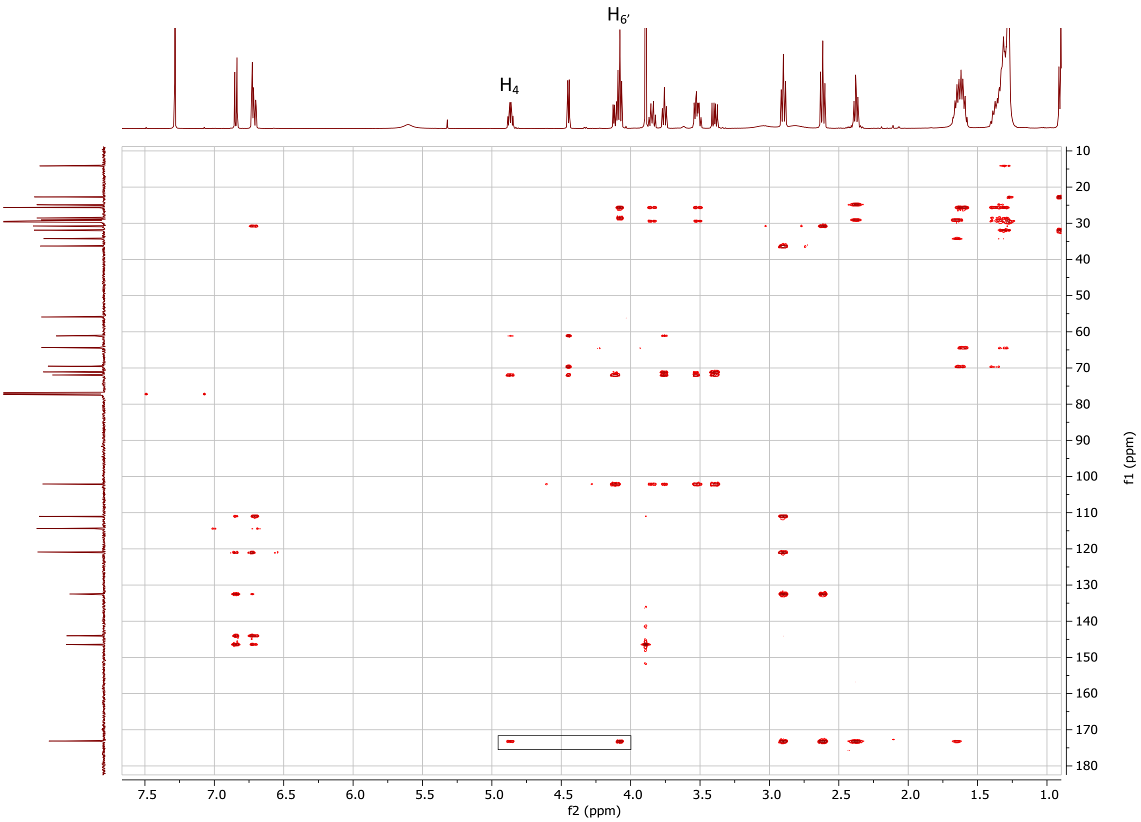
H6'
H4
f1 (ppm)
f2 (ppm)

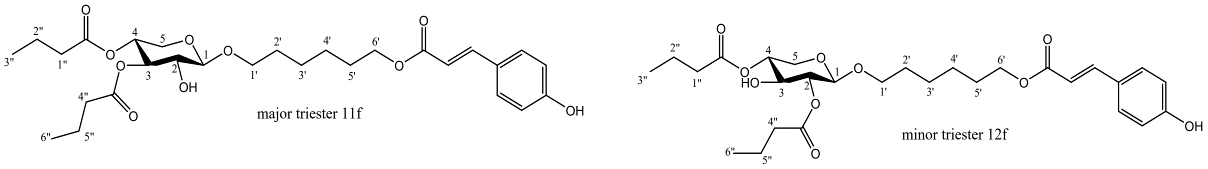
major triester 11f
minor triester 12f

11f-12f

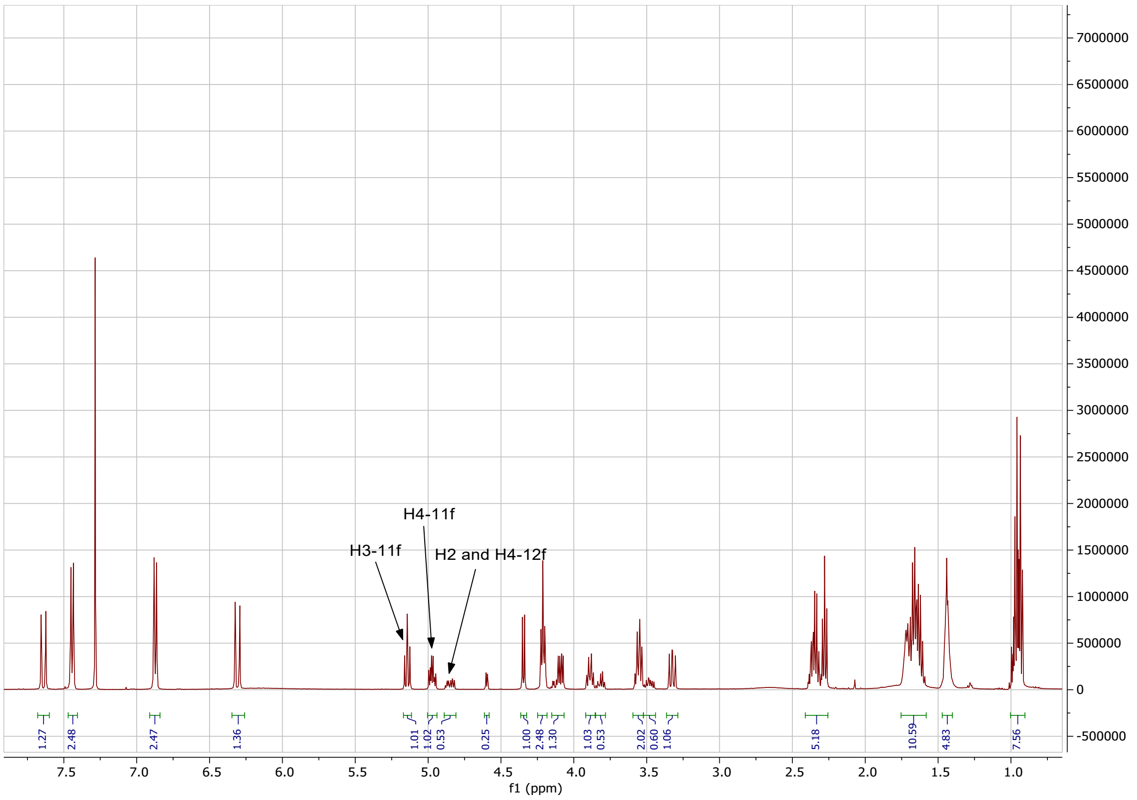

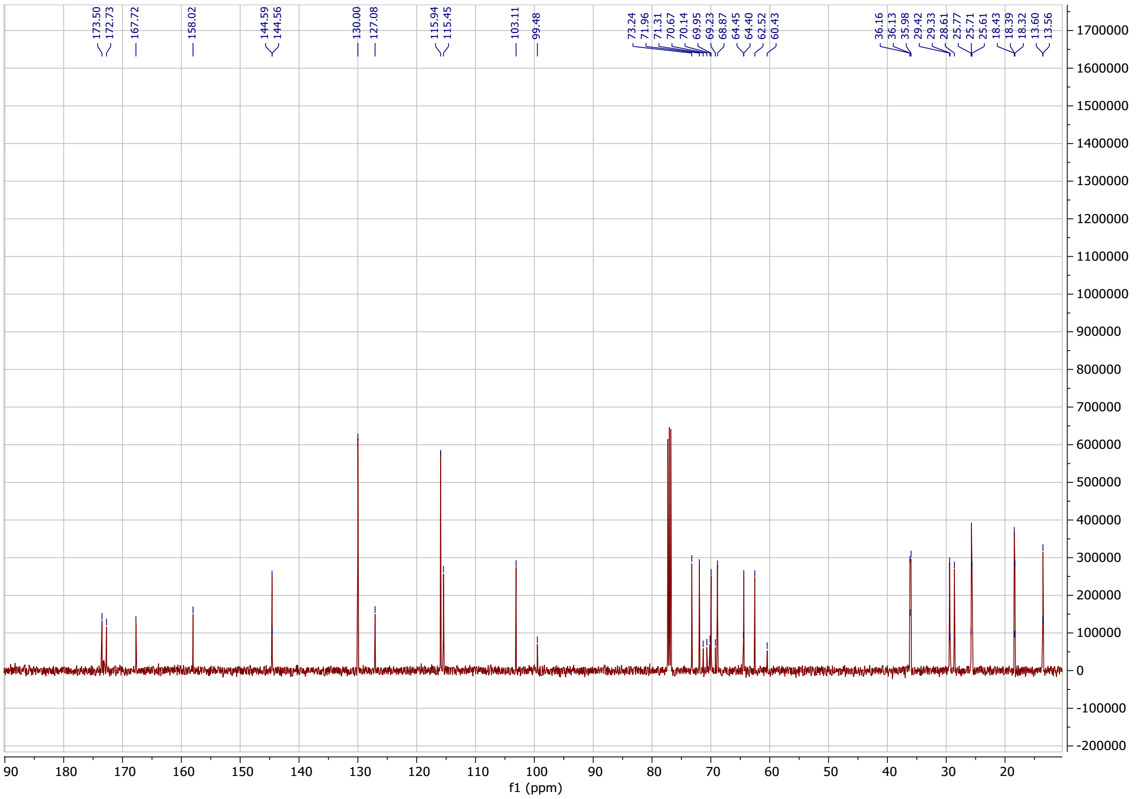

H_3 and H_4-12f

$H_{6'}$

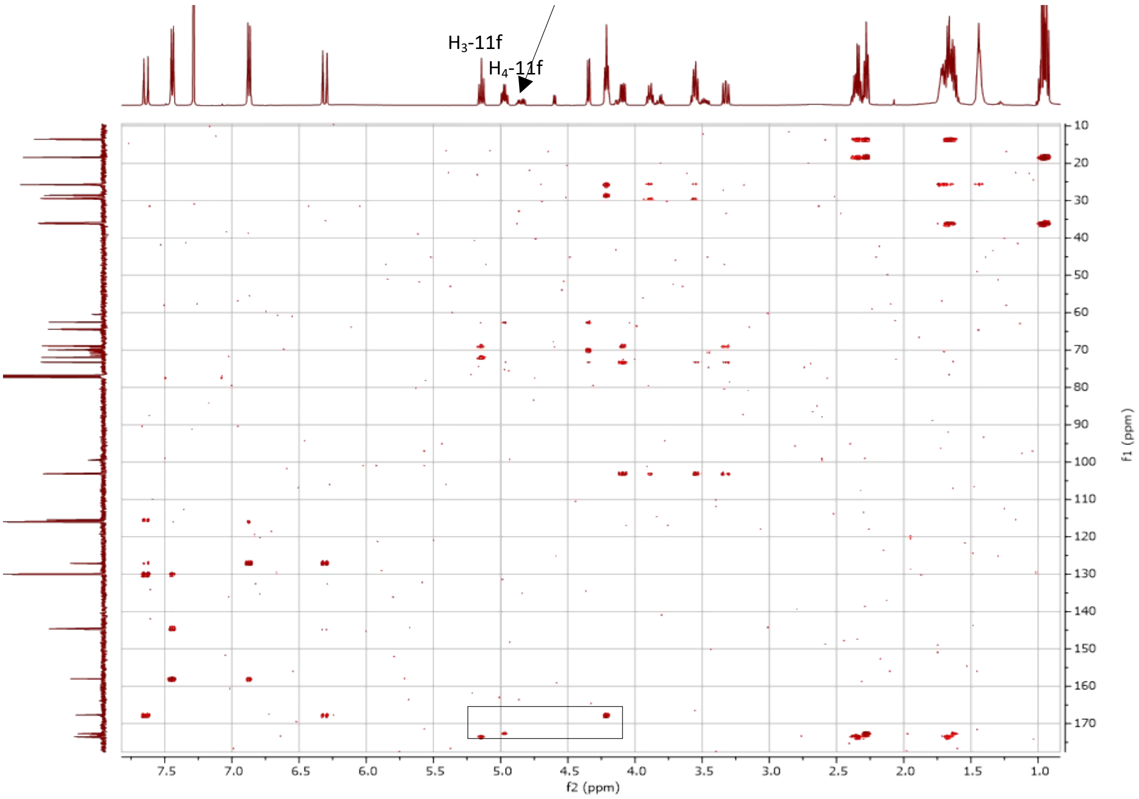
H3-11f
H4-11f
f2 (ppm)
f1 (ppm)

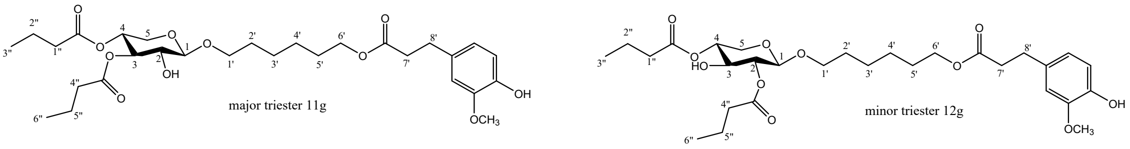
major triester 11g
minor triester 12g

11g-12g

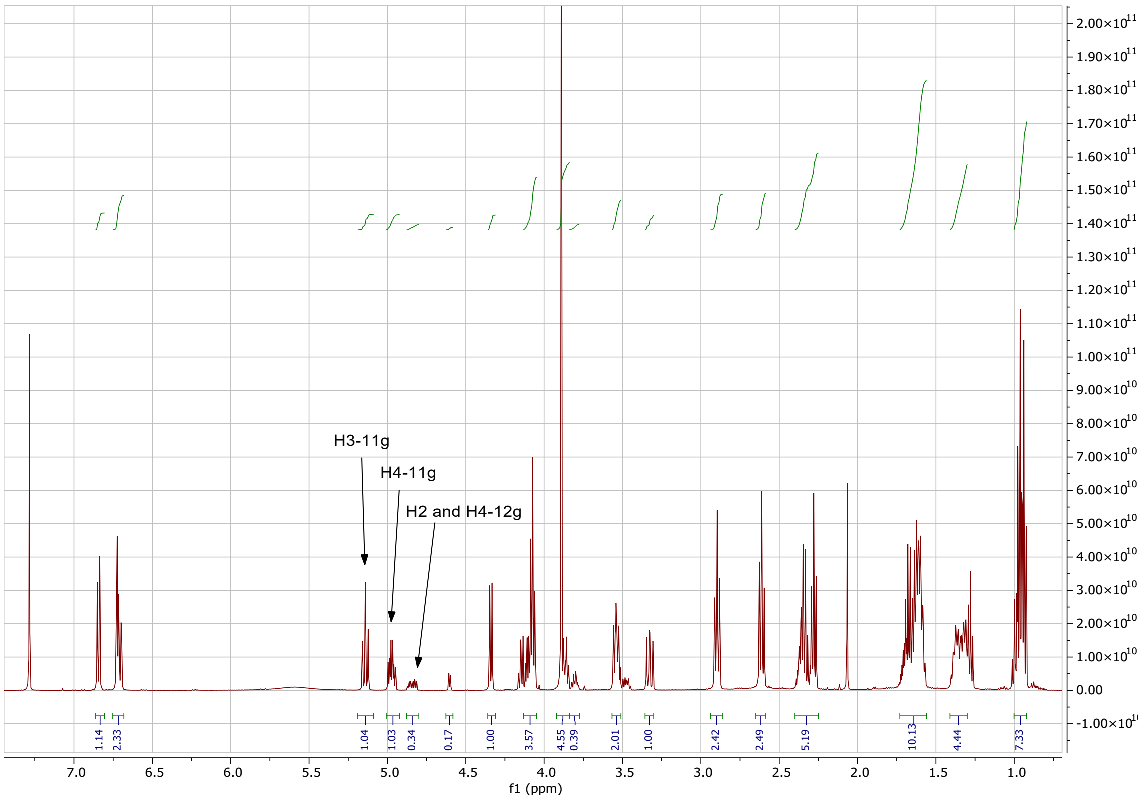
H3-11g
H4-11g
H2 and H4-12g
1.14
2.33
1.04
1.03
0.34
0.17
1.00
3.57
4.55
0.39
2.01
1.00
2.42
2.49
5.19
10.13
4.44
7.33
f1 (ppm)

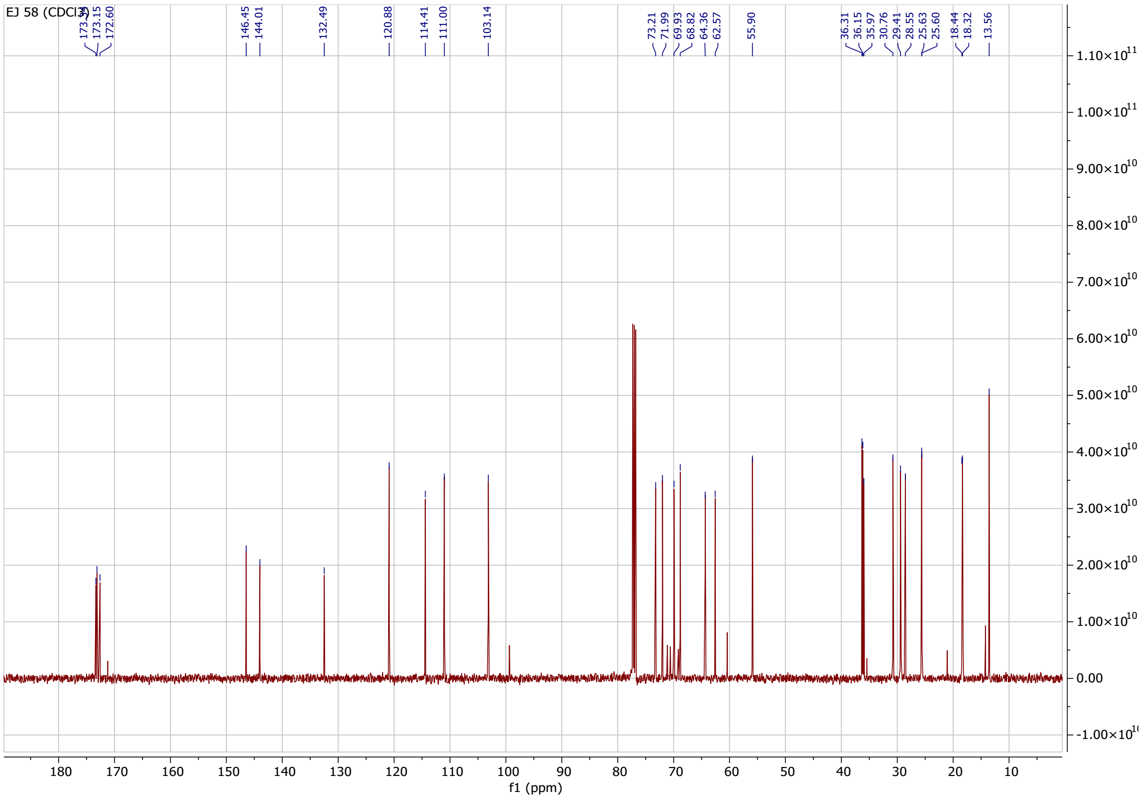
EJ 58 (CDCl3)
173.15
172.60
146.45
144.01
132.49
120.88
114.41
111.00
103.14
73.21
71.99
69.93
68.82
64.36
62.57
55.90
36.31
36.15
35.97
30.76
29.41
28.55
25.63
25.60
18.44
18.32
13.56
f1 (ppm)

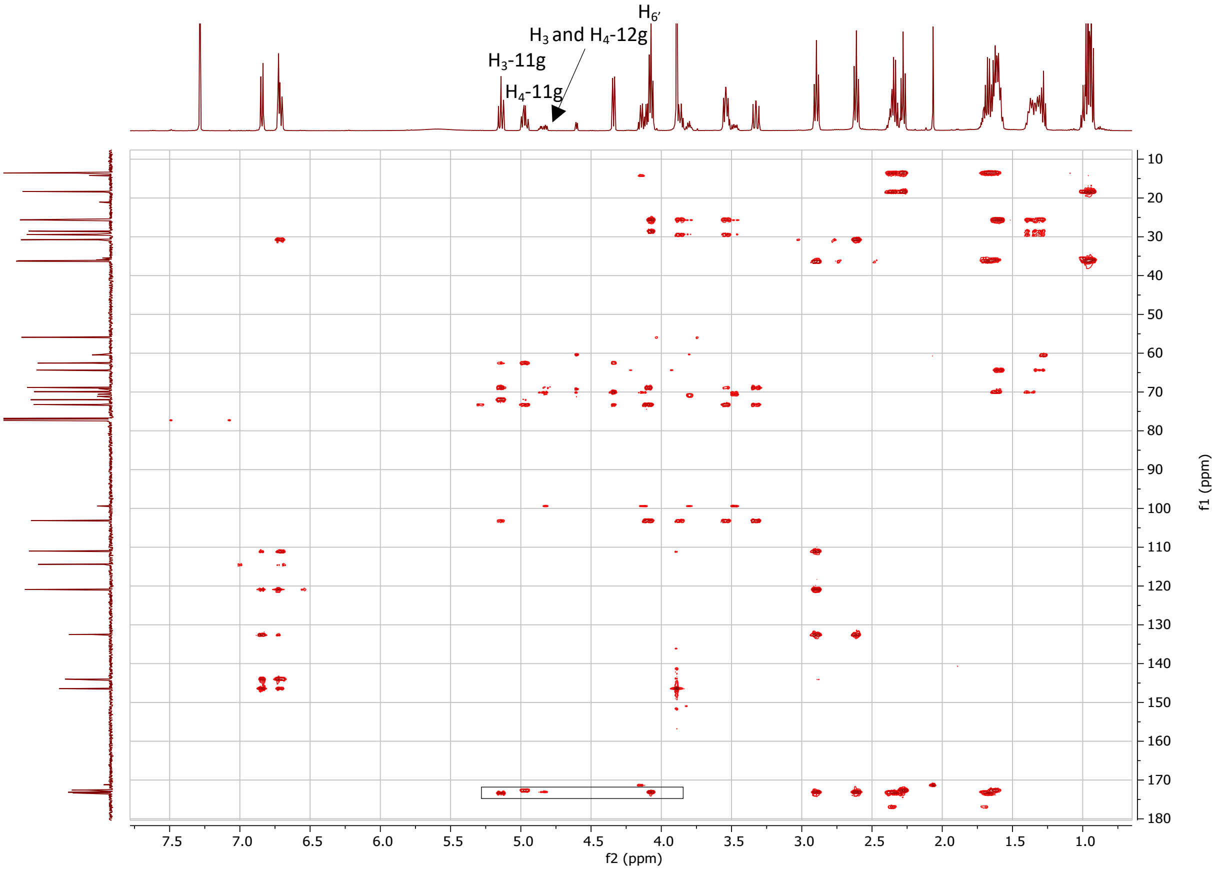

H6'
H3 and H4-12g
H3-11g
H4-11g
f2 (ppm)
f1 (ppm)
